# Homeostasis and metabolism of iron and other metal ions in neurodegenerative diseases

**DOI:** 10.1038/s41392-024-02071-0

**Published:** 2025-02-03

**Authors:** Leilei Chen, Qingqing Shen, Yingjuan Liu, Yunqi Zhang, Liping Sun, Xizhen Ma, Ning Song, Junxia Xie

**Affiliations:** 1 Institute of Brain Science and Disease, Qingdao University, Qingdao, 266071 Shandong China; 2 Shandong Provincial Collaborative Innovation Center for Neurodegenerative Disorders, Qingdao University, Qingdao, 266071 Shandong China; 3 Shandong Provincial Key Laboratory of Pathogenesis and Prevention of Neurological Disorders, Qingdao University, Qingdao, 266071 Shandong China

**Keywords:** Neurological disorders, Diseases of the nervous system

## Abstract

As essential micronutrients, metal ions such as iron, manganese, copper, and zinc, are required for a wide range of physiological processes in the brain. However, an imbalance in metal ions, whether excessive or insufficient, is detrimental and can contribute to neuronal death through oxidative stress, ferroptosis, cuproptosis, cell senescence, or neuroinflammation. These processes have been found to be involved in the pathological mechanisms of neurodegenerative diseases. In this review, the research history and milestone events of studying metal ions, including iron, manganese, copper, and zinc in neurodegenerative diseases such as Parkinson’s disease (PD), Alzheimer’s disease (AD), amyotrophic lateral sclerosis (ALS), and Huntington’s disease (HD), will be introduced. Then, the upstream regulators, downstream effector, and crosstalk of mental ions under both physiologic and pathologic conditions will be summarized. Finally, the therapeutic effects of metal ion chelators, such as clioquinol, quercetin, curcumin, coumarin, and their derivatives for the treatment of neurodegenerative diseases will be discussed. Additionally, the promising results and limitations observed in clinical trials of these metal ion chelators will also be addressed. This review will not only provide a comprehensive understanding of the role of metal ions in disease development but also offer perspectives on their modulation for the prevention or treatment of neurodegenerative diseases.

## Introduction

Neurodegenerative diseases are characterized by neuronal death and loss-of function, which typically result in a gradual decline in cognitive, motor, and sensory functions. Metal ions, such as iron, manganese, copper, zinc, etc., play crucial roles in various physiological processes in the central nervous system (CNS), including energy metabolism, protein synthesis, DNA replication, membrane protein construction, myelin and neurotransmitter synthesis, and so on. The homeostasis of metal ions in the brain is regulated by multiple proteins and molecular mechanisms, which work together to control the process of absorption, storage, and release, thereby maintaining the appropriate concentration and distribution among different brain regions, cells, and organelles. However, once the homeostasis of these metal ions is disrupted, either depletion or accumulation, they can affect the activity of enzymes involved in neurodegenerative diseases since they serve as important cofactors for enzymes. Additionally, an imbalance in metal ions can contribute to the development of neurodegenerative diseases through a variety of mechanisms, including promoting the production and aggregation of pathological proteins, inducing oxidative stress, ferroptosis, cuproptosis, cell senescence, or neuroinflammation. Since iron deposits in the brains of patients with PD and AD were first observed in 1924 and 1953, respectively, the relationship between iron dyshomeostasis and neurodegenerative diseases has attracted more and more attention. Abnormal iron deposition in special brain regions has been proven to be positively correlated with progress development and disease severity in neurodegenerative diseases, such as Parkinson’s disease (PD), Alzheimer’s disease (AD), amyotrophic lateral sclerosis (ALS), and Huntington’s disease (HD). Additionally, other metal ions, such as manganese, copper, and zinc, are also found to participate in the development of neurodegenerative diseases by increasing the risk of neurodegenerative diseases or promoting aggregation of pathological proteins. Notably, the identification of metal ions dependent cell death forms, such as ferroptosis and cuproptosis, have provided new pathological mechanisms in neurodegenerative disease. Although there are many challenges in the development of new drugs for neurodegenerative diseases, such as complex pathogenesis, irreversibility of the disease course, difficulty in penetrating the blood–brain barrier (BBB) and clinical trials, therapeutic strategies targeted metal ions have achieved promising results and offers valuable insights into the prevention and treatment of neurodegenerative diseases.

This review will introduce the research history and milestone events of the study on mental ions (including iron, manganese, copper, and zinc) in neurodegenerative diseases (including PD, AD, ALS, and HD). It will also discuss upstream regulators, downstream effector, and crosstalk of mental ions homeostasis in physiology and neurodegenerative diseases. Given their ability to selectively capture metal ions and dissociate them from target sites implicated in disease progression, chelators offer the potential to minimize side effects associated with broad-spectrum treatments. In this review, we provide a comprehensive summary of the therapeutic effects of various chelating compounds, including clioquinol (CQ), quercetin, curcumin, coumarin, and their derivatives, in the pathology of neurodegenerative diseases. Additionally, we discuss the promising results and limitations observed in clinical trials involving deferiprone (DFP), Cu^2+^-diacetylbis (4-methylthiosemicarbazone)--CuII(atsm), and PBT. This review provides a comprehensive overview of the critical roles redox-active metal ions play in the emergence and progression of neurodegenerative diseases. It emphasizes the need for further research into their mechanisms and the development of effective interventions targeting metal homeostasis as a promising approach for the prevention and treatment of neurodegenerative diseases. Through understanding and modulation of these processes, future strategies could open new avenues for therapeutic intervention.

## Research history of metal ions in neurodegenerative diseases

**Fig. 1:**
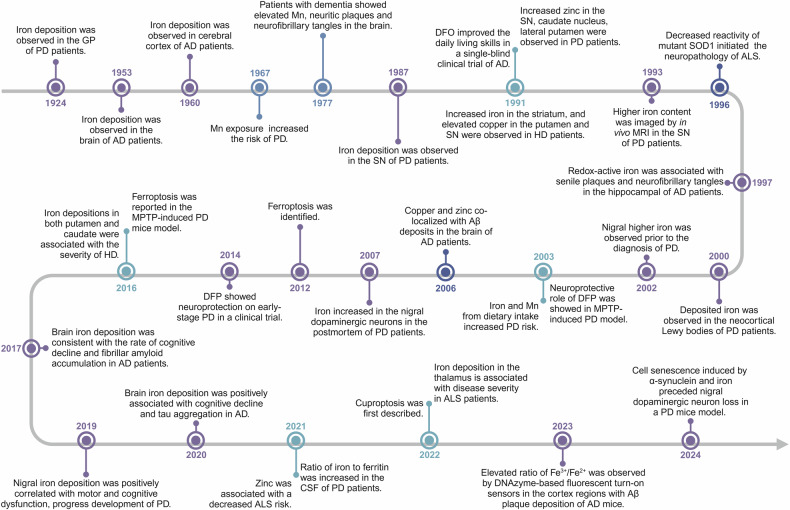
Timeline and milestone events of study on iron and other metal ions in neurodegenerative diseases. The timeline begins at 1924 and expands to 2024. Milestone events of studying metal ions, including iron, manganese, copper, and zinc in neurodegenerative diseases, such as PD, AD, ALS, and HD, are listed in the figure. This figure was created with BioRender.com/d84k316

As early as 1924, iron deposition was first observed in the globus pallidus (GP) of PD patients through Perls’ and Turnbull staining (Fig. 1).^1^ In 1987, Dexter et al. reported significant iron deposition in the substantia nigra (SN) of postmortem PD patient brains,^2^ and subsequent research confirmed increased total iron content in the SN of postmortem PD patients using inductively coupled plasma spectroscopy (ICP-MS).^3–5^ Besides observing higher total iron levels, there was also an increase in ferric iron in the SN of PD patients.^6–8^ In 1993, in vivo magnetic resonance imaging (MRI) revealed a higher iron content in the SN of patients with PD.^9^ In 2000, the application of detecting redox-active iron in situ demonstrated that iron aggregated in the neocortical Lewy bodies of PD patients.^10^ As the main pathological feature of PD, Lewy bodies are composed of a large amount of misfolded α-synuclein, and the toxic couple between iron deposition and α-synuclein aggregation accelerates the progression of PD.^11^ Nigral iron deposition and hyperechogenicity were found in the 6-OHDA-induced PD rat model in 1999.^12^ Subsequently, nigral hyperechogenicity showed higher iron levels prior to the diagnosis of PD in 2002.^13^ In 2003, genetic or pharmacological methods demonstrated that iron chelator presented the neuroprotection in 1-methyl-4-phenyl-1, 2, 3, 6-tetrapyridine (MPTP)-induced PD model.^14^ Furthermore, a clinical trial conducted until 2014 revealed that oral administration of DFP exhibited neuroprotection on early-stage PD patients through chelation of labile iron.^15^ In 2007, a new finding revealed that the iron levels were increased in individual nigral dopaminergic neurons in postmortem PD patients using sensitive and specific wavelength dispersive electron probe x-ray microanalysis coupled with cathodoluminescence spectroscopy.^16^ Idiopathic rapid eye movement sleep behavior disorder (iRBD) is considered a prodromal stage of α-synucleinopathies, such as PD. Within 5 years, 41% of iRBD cases will convent to neurodegenerative diseases, and this rate increases to 73.4% within 10 years.^17^ In 2019, elevated iron was observed in the bilateral substantia nigra of iRBD patients compared to healthy controls by quantitative susceptibility mapping (QSM), and the level of iron in the substantia nigra PD is even higher than that in iRBD, indicating that abnormal nigral iron deposition may be an important factor for accelerating the conversion from prodromal to clinical stage of neurodegenerative diseases.^18^ Furthermore, T2*-weighted magnetic resonance imaging confirmed a positive correlation between nigral iron deposition and the progression of disease, as well as motor and cognitive dysfunction in PD patients.^19^ Although nigral iron deposition has been proven to be positively correlated with the progression of PD, decreased ferritin has been found in several brain regions in PD patients.^3^ In 2021, the ratio of iron to ferritin was first reported to be increased in the cerebrospinal fluid (CSF) of PD patients, which may serve as a potential progression marker for disease progression.^20^ Ferroptosis, a newly named form of programmed cell death in 2012 characterized by iron accumulation and lipid peroxidation, was first observed in an MPTP-induced PD mouse model in 2016.^21^ Acyl-CoA synthetase long-chain family member 4 (ACSL4) can esterify polyunsaturated fatty acids (PUFAs) and trigger ferroptosis.^22^ Increased levels of ACSL4 have been observed in the SN of both MPTP-induced PD mouse model and PD patients, while genetic or pharmacologic inhibition of ACSL4 can specifically prevent the elevation of lipid ROS and ameliorate Parkinsonism phenotypes,^22^ suggesting that interventions in the ferroptosis pathway may become a treatment strategy for PD. Recently, we reported that the toxic interaction between α-synuclein and iron induces cell senescence in a PD mouse model, preceding the loss of nigral dopaminergic neurons.^23^

Iron deposition was first observed in the brain of AD patients with the method of Prussian blue reaction in 1953,^24^ and then subsequently identified in the cerebral cortex of AD patients by the Turnbull blue method in 1960.^25^ A single-blind clinical trial in 1991 demonstrated that desferrioxamine (DFO) treatment could significantly reduce the decline of daily living skills, indicating a positive slowing effect on dementia associated with AD by the iron chelator.^26^ After that, studies on regional brain trace elements received increasing attention. In 1994, in vivo evaluation of brain iron by MRI revealed a higher iron content in the caudate and GP of AD patients.^27^ Additionally, increased ferritin iron accompanied by decreased tissue integrity was identified in the hippocampus of in vivo AD patients by MRI.^28^ In 1997, the associations between redox-active iron and senile plaques and neurofibrillary tangles in the hippocampal tissue of AD patients were reported, and redox-active iron not only participated in the in situ oxidation, but also catalyzed H_2_O_2_-dependent oxidation, implicating iron accumulation as a source of free radicals in AD.^29^ And then redox-iron, which participates in lipid peroxidation and oxidative stress, was reported to mediate the toxicity of Aβ.^30^ According to the neuroimaging using QSM in 2017, brain iron deposition was found to be consistent with the rate of cognitive decline and fibrillar amyloid accumulation, suggesting that brain iron may interact with Aβ to accelerate the clinical development of AD.^31^ Additionally, a study conducted in 2020 reported an association between brain iron and accelerated cognitive decline in AD patients.^32^ Meanwhile, a positive association between iron deposition and insoluble tau aggregates in the inferior temporal gyrus of AD patients was identified by MR-based QSM and tau-PET in 2020.^33^ Recently, DNAzyme-based fluorescent turn-on sensors have been developed, which are selective for either ferrous iron or ferric iron and enable the monitoring of different redox states of iron in living cells.^34^ Using these sensors, increased levels of both ferric and ferrous iron were observed in ferroptotic cells, while a decreased ratio of ferric iron to ferrous iron was observed. Additionally, an elevated ratio of ferric iron to ferrous iron was observed in the cortex regions with Aβ plaque deposition of AD mice.^34^ With a label-free and nanoscale chemical imaging using synchrotron X-ray spectromicroscopy, higher iron level, which presented as chemically reduced and low-oxidation-state phases, was observed in the amyloid plaques of human AD brain tissue,^35^ providing an approach for analyzing the chemical composition of amyloid plaques.

In early 1967, it was first observed that exposure to excessive manganese increased the risk of PD among miners.^36^ In 2003, a study reported a combined effect of iron and manganese intake from diet on increasing the risk of PD.^37^ In 1977, a case report was published on a patient with elevated level of manganese, dementia and an extrapyramidal syndrome.^38^ Both neurotic plaques and neurofibrillary tangles were observed in the brain of this patient. Another case in 1986 reported chronic manganese poisoning causing neuropsychiatric symptoms and neurodegeneration in the basal ganglia, particularly in the pallidum.^39^ In non-human primates exposed to manganese, although the nigrostriatal dopamine system remained intact, subtle motor function deficits were observed, which were associated with decreased dopamine release.^40^ In 2017, exposure to welding fumes containing manganese was reported to induce dose-dependent progression of Parkinsonism.^41^ Recently, it has been proposed that manganese diffuses along white matter tracts. Following manganese exposure, manganese deposition was observed in the cerebellum and frontal cortex as well as the hippocampus using whole-brain MRI relaxometry methods.^42^ After imaging the in situ secondary structure of the amyloid plaques using synchrotron fourier transform infrared micro-spectroscopy (FTIRM) and detecting the metal ions accumulation using synchrotron X-ray fluorescence (SXRF) microprobe in the same brain tissue of AD patients, co-localization of accumulated copper and zinc with Aβ deposits was first observed in 2006.^43^ It wasn’t until 2022 that a copper-dependent form of cell death called cuproptosis was first described, which is mitochondrial respiration- dependent and distinct from known cell death mechanisms.^44,45^ This mechanism has also been found to be involved in neurodegenerative diseases.

In 1991, after analyzing frozen postmortem brain tissue using inductively coupled plasma spectroscopy, researchers observed increased total iron levels in the striatum (putamen and/or caudate nucleus) and elevated copper levels in the putamen and SN of HD patients.^3^ Additionally, increased zinc levels were observed in the SN, caudate nucleus, and lateral putamen of PD patients. In 2016, QSM results revealed significant iron accumulation in the basal ganglia (including pallidum, putamen and caudate) of both premanifest and symptomatic HD patients.^46^ Furthermore, iron accumulation in both putamen and caudate was significantly associated with the disease severity. Notably, significant iron deposition was also observed in the left precentral gyrus and the thalamus, and iron deposition in the thalamus is associated with disease severity in ALS patients in 2022.^47^ Copper/zinc superoxide dismutase, also known as SOD1, exhibits altered reactivity in catalyzing oxidative reactions, which has been suggested to initiate the neuropathologic changes in ALS in 1996.^48^ Additionally, the enzyme activity of mutant SOD1 was found to be reduced by approximately 50% in patients in 1997.^49^ In a recent study conducted in 2021, analysis of metal levels in erythrocytes using ICP-MS revealed an association between decreased ALS risk and zinc, while cadmium and lead were associated with an increased risk of developing ALS.^50^

## Brain metal ions homeostasis in physiological state

### Iron

As an essential trace element, iron functions as a cofactor for many physiological processes, including oxygen transport, DNA synthesis, mitochondrial respiration, and phospholipid synthesis in the brain. One of the main functions of iron in living organisms is to participate in oxygen transport. In red blood cells, iron is a component of hemoglobin, which binds to oxygen and forms oxygenated hemoglobin that transports oxygen from the lungs to the rest of the body. This process is essential for cellular energy metabolism. Iron is an essential component of various crucial enzymes in the enzyme complex of the mitochondrial respiratory chain, particularly the iron-sulfur clusters and cytochromes present in Complex I and Complex II. Iron-sulfur clusters are nonheme cofactors composed of iron and sulfur atoms that play a vital role in electron transport within the electron transport chain. Inadequate levels of iron directly impact the synthesis and stability of these iron-sulfur clusters, thereby hindering the activity of the respiratory streptase complex. Iron is also a component of certain enzymes that are directly involved in key steps in the DNA replication and repair process. For example, iron-sulfur clusters are active centers for enzymes involved in electron transport chains that provide the energy necessary for DNA synthesis. In addition, iron is involved in redox reactions within cells, which are essential for maintaining the reducing environment within cells and preventing oxidative stress damage, which can damage DNA and affect its normal synthesis. In the process of DNA synthesis, iron’s role is not limited to energy supply, but also involves direct participation in the formation of nucleic acid chains. Iron-dependent enzymes such as DNA polymerase play a catalytic role in the synthesis of new DNA strands, helping to link nucleotides together into long strands. These functions of iron are essential for cell growth, division, and the transmission of genetic information. Iron deficiency or excess can affect DNA synthesis and normal cell function. Iron deficiency may lead to a decrease in the rate of DNA synthesis, affecting cell proliferation and differentiation, while iron excess may lead to DNA damage through the production of free radicals. Therefore, maintaining a balance of iron is essential for normal brain function and neurodegenerative diseases.

#### Iron influx into the brain

BBB is one of the major barriers preventing peripheral iron from entering the brain.^51^ During the process of crossing the BBB, endothelial cells, which are the core anatomical structure of BBB, have been suggested to function as gatekeepers. Iron is initially imported by the microvascular endothelial cells at the luminal membrane. In this process, transferrin (Tf) bound ferric iron binds with transferrin receptor 1(TfR1) and enters the cell through endocytosis, while non-transferrin bound iron (known as NTBI) or ferrous iron enters the cell through divalent metal transporter 1 (DMT1).^51,52^ Furthermore, both H-ferritin and Tf could also serve as iron source cross the BBB.^53^ The iPSC-derived brain endothelial cells have been found to uptake H-ferritin through T-cell immunoglobulin and mucin receptor 1, and then secrete H-ferritin into the brain, which process could be affected by DMT1.^53^ In addition to H-ferritin, brain endothelial cells also secrete Tf.^53^ Inhibition of DMT1 by XEN602 could alter the transport of both Tf and iron across the endothelial cells.^54^ With aging, the level of serum ferritin has shown an age-related tendency to rise, and it is higher in males than in females.^55^ Recently, serum ferritin has been suggested to be associated with cognitive performance in aging.^56^ In subjects aged 65 years or older, the level of serum ferritin was found to be positively associated with executive function and language function in males, with evidence of increased cognitive scores of total digits span (TDS), phonemic verbal fluency (PVF), and semantic verbal fluency (SVF).^56^ However, there were no significant associations found between serum ferritin and cognitive scores in subjects aged 50-64 years or in female subjects.^56^ Furthermore, the association of serum ferritin with cognition was found to be regulated by the gut microbiota through microbial-derived metabolites.^57^ Although in a bi-chamber cell culture model of BBB, Tf-mediated transport of radiolabeled iron (^59^Fe) was found to correlate positively with the concentration of plasma hemoglobin but not serum ferritin level,^58^ however, considering that H-ferritin could cross the BBB,^53^ increased serum ferritin may contribute to an increased iron level in some brain regions with aging. In a Belgrade rat model with brain iron deficiency, altered distribution of Tf receptors in the microvasculature was observed in luminal, intracellular, and abluminal membranes which depended on brain iron status.^59^ Ferroportin 1 (FPN1) functions as a gateway for iron release into the brain interstitial spaces at the abluminal membrane of brain microvascular endothelial cells. In mice with deletion of FPN1 from the brain vascular endothelial cells, although the level of iron in the serum was lower, elevated iron levels were observed in the brain vascular endothelial cells, along with evidence of l-ferritin accumulation, implicating the important role of FPN1 in exporting iron from vascular endothelial cells in the brain.^60^ In an in vitro BBB model, iron release from endothelial cells has been found to be stimulated by the iron chelator deferoxamine (DFO) and apo-transferrin (apo-Tf, iron-poor Tf).^54^ The level of FPN in the brain microvascular endothelial cells has been found to be controlled by hepcidin (Hp), which was secreted by astrocytes.^61^ In cultured microvascular endothelial cells, hepcidin peptide significantly reduced Tf-Fe and NTBI uptake and iron release accompanied by downregulation of TfR1, DMT1, and FPN1, whereas knockdown of hepcidin generated opposite results.^62^ In addition, it has been confirmed that human brain microvasculature endothelial cells can express Hp protein and soluble ceruloplasmin (Cp) transcript, and FPN-mediated iron efflux from human brain microvasculature endothelial cells requires endogenous Hp or extracellular Cp, which act as exocytoplasmic ferroxidase.^63^ Recently, it was discovered that holo-Tf (iron-bound) directly interacts with FPN and induces its internalization, while apo-Tf (iron-free) directly interacts with hephaestin in iPSC-derived endothelial cells or HEK293 cells.^64^ Moreover, pathophysiological levels of Hp only disrupt the interaction between holo-Tf and FPN by causing the internalization of FPN, indicating a potential mechanism for apo-Tf and holo-Tf in regulating iron release from endothelial cells.

CSF is another major barrier that prevents peripheral iron from entering the brain.^51^ The blood-CSF barrier is located within the choroid plexus and established by the tight junctions between choroidal epithelial cells, which serve as an important interface to separate the blood from the CSF. Since there is no structural impediment between CSF and interstitial fluid, their components can freely exchange and reach equilibrium. The choroid plexus plays a crucial role in regulating iron homeostasis due to its large surface area and high velocity of blood flow.^65^ Additionally, choroid plexus cells have the ability to synthesize various types of iron transport proteins such as Tf, TfR1, DMT1, FPN1, Cp, and hephaestin.^66–68^ There are two distinct isoforms of Tf, namely the brain-specific Tf and the serum-derived Tf, which demonstrate variations in their glycan configurations.^69^ FPN1 is widely distributed in the cytoplasm but less polarized, whereas TfR is predominantly concentrated in the vicinity of nuclei in the form of clusters and bilateral distributed in choroidal cells.^70^ With the cooperation of DMT1, FPN1/Cp, and FPN1/hephaestin, iron can cross the blood-CSF barrier mediated by choroidal epithelial cells and bind to apo-Tf after entering the CSF.^71^ Besides, due to the presence of fenestrated capillaries on the choroid plexus, Tf-Fe readily combines with TfR1 and enters the choroidal epithelium via endocytosis. Subsequently, it is dissociated in the acidic endosome and transformed into a divalent state through the activity of Steap3, ultimately being transported into CSF via DMT1 and FPN1. However, an alternative perspective on the blood-CSF barrier is that it is also responsible for the clearance and detoxification of iron from the brain. DMT1 is predominantly localized at the apical plasma membrane, exhibiting a polarized pattern that determines the tendency of free iron to flow from CSF to the blood. Experiments have also demonstrated that the total iron content flowing from the CSF to the blood is 128% higher than its influx.^72^ Iron, either bound to Tf or in its free state, is presented in the CSF. Subsequently, the apical microvilli on the choroidal epithelia respectively absorb them, utilizing DMT1 for free iron and TfR1 for Tf-bound Fe, transporting it into the bloodstream. Therefore, it has been established that iron crosses the BBB for entry and exits from the brain through the blood-CSF barrier.^73^

After peripheral iron enters the brain, axonal iron transport may contribute to the distribution of iron among different brain regions. To date, two pathways of axonal iron transport has been reported: one from the ventral hippocampus (vHip) to the medial prefrontal cortex (mPFC) to the SN, and another from the thalamus (Tha) to the AMG to the mPFC.^74^ Notably, the axonal iron transport pathway of vHip-mPFC modulated anxiety-related behaviors in the brain, while the Tha-AMG-mPFC pathway did not. All these pieces of evidence support the hypothesis that dysregulated axonal iron transport can lead to abnormal distribution of iron among different brain regions, thereby causing disease-related symptoms.^75^

#### Iron metabolism in glial cells

Physiological iron levels are not uniform among different types of cells in the brain. In the neocortex, the iron concentration, which was analyzed using a nuclear microprobe and scanning proton-induced X-ray emission spectrometry, is fivefold higher in the oligodendrocytes, threefold higher in the microglia, and twofold higher in the astrocyte than that in the neurons, indicating that glial cells are the most iron-rich cells in the brain.^76^ Therefore, glial cells are considered to be responsible for maintaining iron homeostasis and providing buffering and protection in the brain.^76–78^

As the most abundant glial cells in the CNS, astrocytes perform various crucial functions, including structural support, neurotransmitter transport, injury repair, and inflammation response modulation.^79^ Astrocytes express almost all proteins related to iron metabolism; however, they appear to have a lower metabolic demand for iron but can efficiently store it in ferritin.^80^ Most importantly, once iron crosses the BBB, astrocytes are able to absorb it and then mediate its distribution directly to neurons. Therefore, astrocytes are ideally positioned for iron absorption, distribution, and metabolism.^81^ Hepcidin is an iron-regulatory hormone with widespread distribution in the brain, and its expression in astrocytes has been indicated to regulate iron transport across the BBB and play an essential role in controlling the overall brain iron level. Overexpression of hepcidin in the astrocytes was found to decrease brain iron load, possibly by regulating the FPN1 on the brain microvascular endothelial cells.^61,82^ In the primary cultured astrocytes, hepcidin was also found to regulate iron-related proteins, including TfR1, DMT1, and FPN1, and control iron import. Importantly, hepcidin directly inhibited the expression of TfR1 through a cyclic AMP-protein kinase Α dependent manner.^83^ All these evidences implicate a potential neuroprotective role of astrocyte hepcidin in maintaining iron homeostasis.^84^ Astrocytes can release ferritin. In our recent studies, the process of ferritin release by astrocytes was found to be enhanced by iron overload to buffer extracellular iron.^85^ Notably, the ferritin released by astrocytes can enter and protect dopaminergic neurons by inhibiting the increase of the labile iron pool (LIP) and reducing reactive oxygen species (ROS).^85^ Furthermore, we have reported that ferritin is secreted through transient receptor potential channel 1(TRPML1, mucolipin subfamily)-mediated exocytosis in primary cultured astrocytes.^86^ This secretion can be enhanced by iron treatment and inhibited by autophagy inhibitors 3-MA or chloroquine.

Microglia are the major immune cells in the CNS, constantly moving and removing pathogens and damaged cells, thus protecting neurons from damage. However, when microglia are activated, they can release pro-inflammatory factors that promote neuroinflammation and trigger neuronal damage. Microglia also function as the most efficient iron-absorbing glia cell, which plays an essential role in iron homeostasis.^87,88^ In a tri-culture system consisting of astrocytes, microglia, and neurons from both primary cultured cells and human-induced pluripotent stem cells, microglia were observed to be highly responsive to iron and more susceptible to ferroptosis compared to astrocyte and neurons, which may be due to the different regulation of iron metabolism and the ability to handle iron.^89,90^ The cells reveals more resistance to ferroptosis in tri-culture system compared to that in the monoculture.^89^ Through genome-wide CRISPR screening, *SEC24B* was identified as a regulator of ferroptosis in microglia in addition to *ACSL4*, and knockout cells lacking *SEC24B* showed high resistance to ferroptosis.^90^ Although ferritinophagy, a selective degradation of ferritin through autophagy, has been implicated in promoting ferroptosis, and SEC24B has been indicated to regulate ferroptosis by altering the labile iron pool rather than affecting ferritinophagy, it is also involved in autophagosome formation by binding SEC24A and SEC23B in response to starvation.^90^ Specifically, only activated microglia are capable of synthesizing lactoferrin (Lf).^91^ Although Lf was observed in both human nigral dopaminergic neurons and microglia, only the activated microglia contained the messenger of Lf.^91^ The release of Lf by activated microglia could be enhanced under the treatment with tumor necrosis factor alpha (TNF-α), 1-methyl-4-phenylpyridinium (MPP^+^) or iron overload.^91,92^ Lf could bind with the lactoferrin receptor (LfR) and enter into the nigral dopaminergic neurons through an endocytosis mechanism. In the cellular, there are two forms of Lf: iron-free Lf (apo-Lf) and iron-saturated Lf (holo-Lf). In addition to chelating cellular iron by apo-Lf, both apo-Lf and holo-Lf exhibit neuroprotective benefits by improving Cu/Zn-superoxide dismutase activity, enhancing the mitochondrial transmembrane potential, and increasing the level of Bcl-2.^92^

Oligodendrocytes, which play a critical role in myelination and iron-dependent metabolic enzyme activities, have been found to harbor the highest concentration of iron within the CNS. Cell type-specific iron detection using micro particle-induced X-ray emission (µPIXE) coupled with nickel-enhanced immunocytochemical methods has confirmed that both oligodendrocytes and astrocytes hold the highest level of iron in the SN of non-neurodegenerative control individuals.^93^ Ferritin heavy chain is considered as the major source of iron in oligodendrocytes, which can bind to its receptor, Tim-1(in human) or Tim-2 (in mice), on the membrane of oligodendrocytes and then be imported into cytosol through clathrin-dependent endocytosis.^94–96^ In mice, oligodendrocytes have been identified as expressing a high level of ferritin heavy chain, which can also secrete ferritin heavy chain through extracellular vesicles. Once the secretion or expression of ferritin heavy chain were disrupted in oligodendrocytes, it would cause oxidative damage and neuronal loss, suggesting an antioxidant effect of oligodendrocytes.^97^ DMT1 has been identified as necessary for iron import and development in oligodendrocyte progenitor cells.^98^ Although TfR1 has been found on the membrane of cultured oligodendrocytes, both TfR1 and DMT1 were absent in oligodendrocytes in the adult brain of mice and rats, indicating that the Tf-TfR1 system and DMT1 may only participate in iron import during the immature age of oligodendrocytes.^80,99,100^ The iron export of oligodendrocytes is also facilitated by FPN1, with the assistance of the ferroxidase hephaestin.^67,101^ Although the level and requirement of iron are higher in oligodendrocytes, they are also susceptible to oxidative stress.^102,103^ It has been found that mobilization of iron from ferritin through copper chelation in oligodendrocytes induces demyelination and leads to loss of oligodendrocytes through ferroptosis.^103^

#### Cellular iron metabolism in neurons

Neurons are most vulnerable to iron dysregulation in the CNS. As we summarized before,^104,105^ metabolism of iron in neurons includes the uptake, storage, and export. Briefly, the Tf-TfR system mediates the uptake of ferric iron and DMT1 mediates the uptake of ferrous iron are two major pathways for neuronal iron influx. Ferritin serves as the primary cellular iron storage protein, which can store excess cytoplasmic iron or release it for the synthesis of iron-containing structures. FPN1 is currently known as the only responsible protein for exporting ferrous iron.^106^ With the assistance of ferroxidases, such as Cp, hephaestin, and APP, ferrous iron exported by FPN1 is oxidized to ferric iron and recycled by the Tf-TfR system. As we mentioned previously,^104^ iron regulatory proteins (IRPs) can post-transcriptionally regulate the mRNAs of iron-related proteins (including TfR1, DMT1, ferritin, and FPN1) that contain IREs in the 3′-or 5′-UTRs, and then maintain the cellular iron homeostasis. Lysosomes are critical organelles for intracellular iron storage and have gained increasing recognition.^105,107^ As we summarized previously, lysosomal iron mainly comes from two sources: the degradation of iron-containing substrates through the autophagy-lysosome pathway and endocytosis.^105^ In the acidic and reducing environment of the lysosome, ferric iron is reduced to ferrous iron and then released into cytosolic through DMT1, TRPML1, natural resistance-associated macrophage protein 1 (Nramp1), or two-pore channels (TPCNs).^105^ DMT1, which is localized in the early or late endosome, is mainly responsible for the release of lysosomal iron from Tf-TfR1 recycling. When the lysosomal iron comes from the Tf-Fe_2_ complex or iron-containing cargos, they will be released by TRPML1, which is localized on the late endosome and lysosome membranes, or Nramp1. The selective degradation of ferritin through autophagy-lysosome pathway is known as ferritinophagy, which serves as the exclusive identified mechanism for releasing iron bound to ferritin. Transcription factor EB (TFEB) acts as a master regulator of both lysosomal biogenesis and autophagy. Our recent study has reported that overexpression of TFEB can upregulate TfR1 synthesis through the FBXL5-IRP2 pathway and increase the localization of TfR1 in lysosomes, which facilitates lysosomal iron import and transient lysosomal iron storage.^108^ TRPML1 also serves as a lysosomal calcium release channel, which process is involved in the clearance of α-synuclein. In cultured cells expressing TRPML1/2, iron overload triggers an increase in cytosolic ferrous iron and cytotoxicity, and overexpression of TFEB increases the number of iron-positive endolysosomes and promotes lysosomal exocytosis, a process that depends on TRPML1/2 mediated calcium release and can rescue apoptosis induced by iron overload.^109,110^ Mitochondria are another important organelle involved in iron metabolism. Under normal conditions, voltage-dependent anion channel (VDAC), Tf-TfR2, and DMT1, all located on the outer mitochondrial membrane, are responsible for the iron cross outer mitochondrial membrane. The transport of iron across the inner mitochondrial membrane is mediated by mitoferrin 1/2 (Mfrn1/2). In the mitochondrial matrix, iron can be utilized for the biogenesis of Fe/S cluster and heme or stored in mitochondrial ferritin (FtMt). The transport of both Fe/s cluster and iron from mitochondria into cytosol is mediated by the ATP-binding cassette subfamily B member 7 (ABCB7/8).^111^

### Other metal ions

#### Manganese

As a crucial micronutrient, manganese functions as an essential cofactor for various proteins, especially for manganese metalloenzymes, such as arginase, pyruvate carboxylase, glutamate synthetase and manganese superoxide dismutase (MnSOD, also called SOD2), and plays a significant role in maintaining the normal physiological function of CNS.^112^ Glutamine synthetase is considered the most prominent Mn-rich protein, predominantly expressed in astrocytes and catalyzing the conversion of glutamate to glutamine.^113^ And pyruvate carboxylase is necessary for interacting with manganese to produce oxaloacetate, which then undergoes the TCA cycle.^114^ In the reactive catalytic center, SOD2 can prevent cells from oxidative stress by mitigating the generation of ROS within mitochondria. Additionally, manganese has the capacity to activate ATM protein kinase and tumor suppressor p53, which regulate cell cycles and reduce DNA damage.^115^ To achieve a delicate balance between its indispensability and neurotoxic implications, the uptake of manganese in neurons and glial cells is rigorously regulated, including both receptor-mediated endocytosis and non-transferrin-mediated uptake. Trivalent manganese can bind with Tf, however, the transfer velocity is relatively slow. Once manganese is internalized through TfR, it is converted into its divalent form and subsequently transported to the cytosol via DMT1. After absorption, manganese primarily accumulates in the basal ganglia region, particularly in the striatum and SN. The majority of manganese binds to manganese metalloproteins, particularly glutamine synthetase within astrocytes. manganese was also found adjacent to the nucleus of dopaminergic neurons in SN.^116^ SLC30A10 is a cell surface protein involved in the efflux of manganese, and deficiency in manganese efflux transporter SLC30A10 causes ~20–60-fold higher level of manganese level in the brain, indicating a protection role of SLC30A10 against neurotoxicity.^117,118^ Patients with a mutation in SLC30A10 exhibit significantly elevated levels of manganese in their blood and all of them present with Dystonia or Parkinsonism. However, an effective treatment option is oral iron supplementation, which may enhance the competition between iron and manganese for transporters SLC30A10.^119^ The iron exporter FPN is also involved in the efflux of manganese, which could reduce manganese cytotoxicity and accumulation.^120^ However, this process can be inhibited by low extracellular pH and high K(+) in the medium.^121^

#### Copper

The brain has the second highest amount of copper (Cu), which is essential for respiratory functions and defense itself by generating radicals, as well as generating neuroendocrine peptides and hormones. Additionally, due to the variable oxidation form of Cu^+^ and Cu^2+^, copper also serves as a cofactor for several enzymes, such as copper/zinc superoxide dismutase (CnZnSOD, also called SOD1), Cp, and so on. SOD1 serves as the first protector against damage from reactive oxygen species (ROS) and superoxide anion (O2•−) radicals in both the cytosol and mitochondrial intermembrane space.^122^ Cp belongs to the multi-copper oxidase family of enzymes and primarily functions as ferroxidase in vivo, which can convert toxic ferrous iron into nontoxic ferric form while playing an important role in iron homeostasis.^123^ Both BBB and CSF also function as barrier systems for the transport of copper and restrict the permeability of copper into the brain strictly, which has been summarized recently.^124^ The cellular uptake, efflux, and distribution of copper are regulated by membrane-integrated copper transporters. Copper transporter 1 (CTR1) is the primary mechanism for copper uptake, which occurs independently of ATP hydrolysis and can be stimulated by acidic extracellular pH and elevated concentrations of K^+^ ions.^125,126^ CTR1 heterozygous and homozygous deletion mice exhibit defects in the activities of copper-dependent enzymes, as well as growth and development.^127^ In mice with knockdown of CTR2, significant copper accumulation has been observed, which may depend on the cleavage function of CTR2 to CTR1.^128^ This is because a truncated form of CTR1 protein can facilitate the export of copper from the endosome. DMT1 also participates in the transportation of bivalent copper, and there is a competitive relationship between the uptake of copper and iron by DMT1. In cells with knockdown of DMT1, increased uptake of Cu^1+^ and reduced uptake of Cu^2+^ were observed.^129^ Meanwhile, Cu^1+^ inhibits the transport of Fe^2+^, while Fe^2+^ can inhibit the uptake of Cu^1+^. The ATPase proteins ATP7A and ATP7B play an important role in copper export mediated by copper chaperone antioxidant protein 1 (Atox1).^130^ The absence of ATP7B could cause hepatic copper accumulation and subsequently increase the concentration of copper in the brain.^131–133^

Under normal conditions, cells contain extremely low levels of free copper due to the sequestration by GSH, storage in metallothioneins complex, and transportation by copper chaperones such as Atox1 and copper chaperone for superoxide dismutase (CCS). Firstly, the most important intracellular copper chelator is GSH, which forms complexes and serves as a reservoir of copper. Cu (I)–GSH can deliver copper to metallothioneins, SOD1, and Atox1 while preventing damage to host cells caused by free copper-induced ROS. However, the Cu (I)–GSH complex is redox-active and also has the ability to generate Fe^2+^ that is redox-active and facilitate a superoxide-driven Fenton reaction.^134^ Moreover, GSH could affect the metabolism of copper. Although overexpression of GSH upregulated both the level of CTR1 and the rate of copper transport, it also decreased the bioavailable copper pool.^135^ In addition, GSH could also regulate the activity of both ATP7A and ATP7B.^136^ Metallothioneins also function as a detoxification and neuroprotective substance by binding with copper or zinc, which plays a crucial role in the safe storage of copper.^137^ As a copper chaperone, Atox1 functions as a carrier for copper and facilitates its transfer to the ATPase-mediated secretory pathway.^130^ Atox1 also regulates the activity of ATP7B through modulating domain dynamics.^138^ Recently, evidence has shown that Atox1 plays a pro-inflammatory role in a mouse model of intestinal inflammation, with increased levels of pro-inflammatory cytokines and regulation of the ROS-NLRP3 pathway.^139^ CCS, another copper chaperone serves as a transporter and activator of copper for superoxide dismutase, also functions as a target of the X-linked inhibitor of apoptosis (XIAP), which could be ubiquitinated by XIAP and enhances its chaperone activity to SOD1.^140^The acquisition and distribution of CCS-dependent copper were found to largely occur at membrane interfaces.^141^

#### Zinc

Zinc (Zn), the second most abundant essential transition metal, plays a fundamental role in the immune system, acts as a cofactors for enzymes, functions as intracellular second messengers, and has a neuroprotective role in the brain. The metabolism and signaling pathway of zinc have been summarized by Chen et al. recently,^142^ mainly focusing on peripheral and cancer. The concentration of zinc in the brain is also strictly regulated by three substances: metallothioneins, zinc transporters (ZnTs), and Zrt- and Irt-like proteins (ZIPs). In addition to copper, metallothioneins can also bind and release zinc. However, abnormal release of zinc from metallothioneins-3 could cause neuronal death in the thalamus and CA1 region of hippocampus.^143^ The ZnT family comprises ten distinct members (ZnT1-10), characterized by six transmembrane domains and intracellular termini of NH_2_ and COOH. These proteins play a distinct role in removing intracellular zinc by transporting it into various intracellular compartments and effluxing it from the cytosol. In the monkey spinal cord, ZnT1-10 can be observed in both motor and sensory neurons, but not in glial cells, indicating its potential role in motor and sensory functions.^144^ ZnT1 is responsible for transporting zinc out of the neurons,^145,146^ and also functions as a neuronal Zn^2+^/Ca^2+^ exchanger, playing an important role in neuronal signaling.^147^ ZnT3 is an indispensable component of the activity-dependent release of zinc from neurons, residing on synaptic vesicles and serving as the sole pathway for transporting zinc into these vesicles. Mice lacking ZnT3 exhibit increased vulnerability to epileptic episodes and cognitive loss, indicating the potential role of ZnT3 in regulating changes in zinc levels.^148–150^ ZIP family consists of four subfamilies, namely ZIP I, ZIP II, gufA, and the LIV-1 subfamily. These subfamilies are responsible for importing zinc from the extracellular into the cytoplasm. FPN1 is also involved in zinc transport, and zinc can induce binding between transcription factor-1 (MTF-1) and the FPN1 promoter, thereby affecting its expression.^151^

### Crosstalk and downstream effectors of iron and other metal ions

**Fig. 2:**
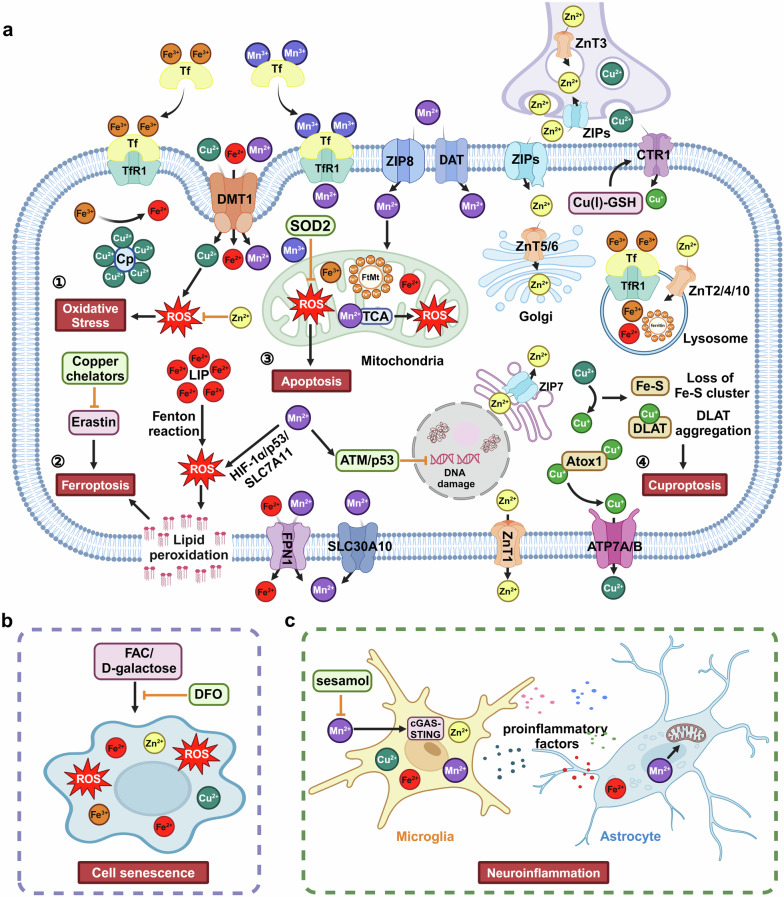
Downstream effectors of iron and other metal ions. **a** The import of iron mainly depends on TfR1-mediated endocytosis or DMT1, while FPN1 is the sole known exporter of iron. The import of copper mainly depends on CTR1, which can be upregulated by Cu(I)-GSH. DMT1 also participates in the import of copper. The export of copper is mediated by ATP7A/7B with the assistance of Atox1. As a copper-binding protein, Cp also functions as a ferroxidase to convert toxic ferrous iron into nontoxic ferric iron. Excessive copper leads to the aggregation of lipoylated proteins and the loss of iron-sulfur cluster proteins, resulting in cuproptosis. The import of manganese relies on the DAT, ZIP8, Tf/TfR system, or DMT1, while the export of manganese depends on SLC30A70 and FPN1. Manganese can activate ATM/ p53, which regulates cell cycles and reduces DNA damage. In the mitochondria, Mn^2+^ can bind to intermediate products of the TCA cycle and promote the generation of ROS, while Mn^3+^ can help SOD2 to mitigate the generation of ROS and prevent cells from undergoing apoptosis. ZIPs are the main channels for transporting zinc into the cytoplasm from extracellular or ER, while ZnTs are responsible for transporting zinc out of the cytoplasm or into synaptic vesicles, lysosomes, and Golgi. Both ferrous iron and copper can promote the Fenton reaction, leading to the generation of ROS and initiating oxidative stress. However, zinc can compete with copper or iron, thereby preventing the generation of ROS. Both excessive Fe^2+^ and Mn^2+^ iron can lead to the accumulation of lipid peroxidation and trigger ferroptosis, which can be inhibited by copper chelators. **b** High levels of metal ions, including iron, manganese, copper, and zinc have been found to be involved in cell senescence. This process can be rescued by the iron chelator DFO. **c** Excessive metal ions such as iron, manganese, copper, and zinc can also activate microglia and astrocytes to release pro-inflammatory cytokines, thereby triggering neuroinflammation. This figure was created with BioRender.com/j25f189

The most common downstream effector of metal ions accumulation is oxidative stress (Fig. 2). Both ferric iron and copper can promote the process of Fenton reaction, resulting in generation of damaging ROS and initiating oxidative stress, which leads to damage cell membrane, proteins, as well as nuclei acids.^152^ Due to the similar structure between iron and manganese, there is a competitive relationship between iron and manganese in DMT1. After treating with 500 µM manganese in Caco2 cells, a conspicuously reduction in the absorption of iron by DMT1 was observed.^153^ Therefore, iron significantly impacts manganese homeostasis. Expanding into clinical application, oral iron therapy could attenuate symptoms of excessive manganese through competition for transporters.^119^ On the other hand, exposure to manganese translational inhibited the 5′-untranslated regions of H-ferritin and APP in a dose- and time-dependent manner, thereby resulting in iron accumulation and neurotoxic oxidative stress.^154^ The exposure to a high level of manganese also induces oxidative stress by oxidizing dopamine or interfering with normal mitochondrial respiration.^155^ Copper can also engage in redox reactions, which is a double-faced sword. The advantage lies in its participation in biochemical reactions through its redox activity. On the contrary, excessive copper can trigger the production of free radicals through the Fenton and Haber–Weiss reactions, leading to oxidative stress and neuronal death.^156^ Although there is no direct connection between zinc and ROS, a complex composed of zinc, ROS, and protein thiols has been linked zinc to the redox-signaling pathway.^157^ Additionally, zinc has been found to reduce the overproduction of ROS and improve oxidative stress.^158^

As an iron-dependent cell death form, ferroptosis is first described in 2012 and characterized by iron accumulation, depletion of GSH, and lipid peroxidation.^159^ Recently, the signaling pathways and involvement of iron in ferroptosis has been detailed summarized by Carsten Berndt et al.^160^ The selective degradation of ferritin through autophagy-lysosome pathway is known as ferritinophagy, which is mediated by the selective cargo receptor NCOA4.^161^ As ferritinophagy could release iron from ferritin, it has been reported that autophagy promotes the process of ferroptosis, which is also known as an autophagy-dependent cell death form.^162,163^ However, autophagy has two sides, and self-protective autophagy has been demonstrated to be beneficial for neurodegenerative diseases. Iron overload can disrupt the fusion of autophagosomes and lysosomes by decreasing Rab7, which causes autophagosome accumulation and blocks autophagic flux in microglia.^164^ In the L6 skeletal muscle cells, iron overload promotes the production of ROS, inhibits the self-protective autophagy, ultimately leading to apoptotic cell death.^165^ Our recent study has reported that TFEB-mediated autophagy maintains cellular labile iron at a low level and prevents ferroptosis in a TfR1-dependent manner.^108^ In addition, cell density can modulate iron regulatory protein 1 (IRP1) to affect the levels of FPN and TfR1, resulting in changes in cellular iron levels that are critical for susceptibility to ferroptosis.^166^ Zinc is also involved in ferroptosis, and zinc addition can promote ferroptosis even when iron chelation is present.^167^ With genome-wide RNAi screening, SLC39A7 (ZIP7), which is responsible for transporting zinc from the endoplasmic reticulum (ER) to the cytosol, has been identified as a genetic determinant of ferroptosis.^167^ In the vascular endothelial cells, zinc oxide nanoparticles induce ferroptosis through NCOA4-mediated ferritinophagy.^168^ Recently, mitophagy was also found to be involved in zinc-induced ferroptosis in porcine testis cells.^169^ Manganese was found to induce ferroptosis by inhibiting the mitochondrial dihydroorotate dehydrogenase in tumor cells.^170^ Both in vivo and in vitro results support that exposure to manganese causes increases in lipid peroxidation, ferrous iron, as well as ROS, which triggers ferroptosis and neurotoxicity.^171^ Additionally, manganese-induced ferroptosis in dopaminergic neurons is also mediated through the pathway of HIF-1α/p53/SLC7A11.^171^

Described for the first time in 2022, cuproptosis is a novel form of regulated cell death that is distinct from known cell death mechanisms and relies on copper and mitochondrial respiration.^44,45^ During the process of cuproptosis, copper directly binds to lipoylated components of the tricarboxylic acid (TCA) cycle, resulting in aggregation of lipoylated proteins and loss of iron-sulfur cluster proteins.^44^ Copper exposure was also found to cause cognitive impairment in mice, which was related to the modulation of cuproptosis, damaged synaptic plasticity, and inhibition of the CREB/BDNF pathway.^172^ Copper has been found to bind with GPX4 at the cysteines C107 and C148 and promote the degradation of GPX4 through Tax1 binding protein 1 mediated autophagy, thereby driving ferroptosis.^173^ Additionally, copper chelators tetrathiomolybdate and tetraethylenepentamine could block ferroptosis induced by erastin but not other types of cell death.^173^ Furthermore, there is a profound interplay between iron and copper that can influence their transport under abnormal concentrations. In an iron-deficient rat model, a significant increase (+55%) in the level of copper in the CSF and brain parenchyma is observed. Under the ventriculo-cisternal perfusion, the clearance of copper from the CSF is remarkably augmented in iron-deficient rats. This may be attributed to an upregulation of DMT1 expression due to the deficiency of iron rather than CTR1.^174^

Cell senescence, a process characterized by gradual declines in cell proliferation, differentiation, or physiological function, was first proposed by Hayflick and Moorhead in 1961.^175^ The main characteristics of cell senescence include (1) increased activity of senescence-related β-galactosidase (SA-β-gal), lipofuscin accumulation caused by lysosomal dysfunction, and mitochondrial dysfunction; (2) activation of senescence-related signal pathways in p53-p21-pRB or p16-pRB; (3) the appearance of senescence-related secretory phenotype; (4) macromolecular damages in DNA, protein or lipid, and so on. Although cell senescence is a mechanism of mitotic cell cycle arrest, it also occurs in post-mitotic cells, such as terminally differentiated neurons.^176^ As a hallmark of aging, cell senescence occurs in many types of cells in the CNS, including neurons, astrocytes, microglia, oligodendrocytes, and neural stem cells.^177,178^ Recently, iron, whether in its free form or released by lysed erythrocytes, has been found to induce ROS-mediated cell senescence.^179^ Furthermore, iron accumulation in the senescent cells could drive the development of senescence-related secretory phenotype. Additionally, iron overload induces cell senescence in both the brain vasculature and brain tissue itself, which phenomenon is associated with the downregulation of Robo4 in the cerebral endothelial cells derived from aged female mice.^180^ Iron overload resulting from NCOA4-mediated ferritinophagy could cause mitochondrial dysfunction and trigger mitochondrial DNA release, leading to cell senescence through the cGAS-STING pathway.^181^ Meanwhile, the iron chelator DFO could significantly rescue retinal pigment epithelial senescence induced by ferric ammonium citrate or D-galactose in mice.^181^ Notably, iron accumulation in senescent cells has been found to be coupled with impaired ferritinophagy and inhibition of ferroptosis.^182^ Elevated copper was also observed in senescent MEF cells, which may be caused by an increase in CTR1 and a decrease in ATP7A accompanied by enhanced antioxidant defense.^183^

In addition, neuroinflammation also acts as a downstream effector of metal ions, mainly mediated by the activation of microglia or astrocytes in the CNS, which promotes the release of pro-inflammatory cytokines. As microglia are the most efficient iron-absorbing glial cells in the CNS, it is well known that iron can activate microglia and promote the secretion of pro-inflammatory cytokines, resulting in neuroinflammation. Other metal ions, such as manganese, copper, and zinc, also exert a regulatory effect on neuroinflammation. Exposure to copper increases the microglial secretion of pro-inflammatory cytokines, including IL-1β, TNF-α, and IL-6, thereby elevating neuroinflammation both in vitro and in vivo.^184^ Copper-induced neuroinflammation is mediated through the ROS/NF-κB pathway and autophagy impairment.^185^ Although zinc, in a state of homeostasis, inhibits microglia-mediated neuroinflammation, both zinc depletion and zinc accumulation can promote neuroinflammation, which has been detailed and summarized.^186^ Manganese exposure induces neuroinflammation not only in microglia,^187^ but also in astrocytes through impairing mitochondrial dynamics.^188^ Manganese dose-dependently increases the levels of pro-inflammatory cytokines and chemokines, such as IL-6, TNF, CCL2, and CCL5, in microglia, and the pro-inflammatory cytokines released by microglia could dramatically enhance the mRNA levels of TNF, IL-1β, IL-6 in astrocytes, while inhibiting the NF-κB pathway in the microglia could block microglial-induced astrocyte activation.^187^ This indicates that manganese induces inflammatory responses in microglia, which amplifies the inflammatory activation of astrocytes through the NF-κB pathway. In addition, manganese could activate the cGAS-STING pathway in microglia, increase the expression of proinflammatory mediators, and induce neuroinflammation, which could be reduced by sesamol.^189^ Intranasal exposure to a high dose of manganese induces neuroinflammation, which is accompanied by disruptions in dopamine metabolism in both the striatum and hippocampus of rat.^190^ Autophagy is also involved in the manganese-induced neuroinflammation, including SIRT1/FOXO3-mediated autophagy signaling,^191^ glycogen synthase kinase-3β (GSK-3β) signaling,^192^ and NLRP2-CASP1 signaling.^193^ Manganese could increase the level and activity of LRRK2, a kinase that has recently been found to be involved in manganese-induced neuroinflammation in microglia.^194^ The activation of RAB10 by manganese-LRRK2, which is exacerbated by the LRRK2 mutation G2019S, dysregulates the microglial autophagy-lysosome pathway and NLRP3 inflammasome.^194^

## The role and mechanism of iron and other metal ion dysregulation in neurodegenerative diseases

### Brain regional redistribution of iron and other metal ions

#### Methods of detecting metal ions in the brain

MRI is a non-invasive and sensitive method that has been widely used to detect the distribution and very low concentration of metal ions in the brain, especially in neurodegenerative diseases.^9,28^ T2*-weighted MRI is a specific sequence in MRI techniques, and QSM is a sensitive MRI technique. Both T2*-weighted MRI and QSM are widely used to evaluate the levels of metal ions in the brain of individuals with neurodegenerative diseases.^18,19^ Furthermore, T2*-weighted magnetic resonance imaging confirmed a positive correlation between nigral iron deposition and the progression of the disease, as well as motor and cognitive dysfunction in PD patients.^19^ Transcranial sonography (TCS) is also employed as a non-invasive method for detecting iron deposition in the midbrain. It has been suggested that TCS and MRI parameters should be considered complementary in the detection of iron deposition in PD.^195,196^ Magnetic Sensitivity weighted imaging (SWI) is a special MRI technique that is highly sensitive to magnetic differences in tissues. By combining the high spatial resolution and phase information of the gradient echo sequence, SWI can detect subtle changes in magnetic susceptibility more effectively, enabling better visualization of vascular structures, bleeding, and iron deposition.^197^ X-ray fluorescence microscopy (XFM) is a technique used to detect and image the distribution of elements in a sample, specifically metal ions. Due to its ability to provide high spatial resolution information about the types and concentrations of elements in a sample, XFM has been employed for detecting the distribution of metal ions in the brain, which is crucial for understanding their role in neurodegenerative diseases.^198^ Furthermore, the combination of synchrotron X-ray fluorescence (SXRF) microprobe and synchrotron Fourier transform infrared micro-spectroscopy (FTIRM) allows for the assessment of metal ions co-localization with aggregated proteins.^43^

Mass spectrometry (MS) is a technique that can be used for the quantitative analysis of metal ions and the detection of their concentration in biological samples, such as CSF and brain tissue. ICP-MS is a technique developed based on MS, which has high sensitivity and a wide dynamic range. It is capable of detecting elements from ultra-trace to major levels. ICP-MS operates by introducing the sample into an inductively coupled plasma, ionizing the elements in the sample into ions, which are then separated and detected by MS.^3–5^ Laser ablation inductively coupled plasma mass spectrometry (LA-ICP-MS) combines laser ablation technology with ICP-MS. In this technique, a laser beam is used to remove tiny materials from the surface of a solid sample, and the resulting aerosols are directly transmitted to the ICP source for ionization and analysis. LA-ICP-MS enables in situ microzone analysis of solid samples, allowing for element and isotope imaging as well as quantitative analysis at the micron or even nanoscale.^199^ Atomic absorption spectroscopy (AAS) is another technique used to detect the concentration of metal ions. It is based on the principle that atoms absorb specific wavelengths of light and can be utilized for quantifying the metal ions present in a brain sample.^200–202^ Inductively coupled plasma atomic emission spectrometry (ICP-AES) has been used to detect the levels of iron in the blood and serum,.^203^

Histopathological methods, such as Perls’ and Turnbull staining,^1^ allow for the observation of metal ion deposition in postmortem brain tissue through microscopic examination of tissue sections. Recently, fluorescent turn-on sensors based on DNAzymes have been developed that are selective for either ferrous or ferric iron and enable the monitoring of different redox states of iron in living cells.^34^

#### Brain regional metal ions redistribution in PD

ICP-MS results have shown that although there were a significant increase in iron level and a decrease in ferritin level in the CSF samples from PD patients (Table 1), the ratio of iron/ferritin was significantly increased, indicating that iron-ferritin ratio in the CSF may serve as a potential progression marker for PD.^20^ In a postmortem study, significantly increased permeability of BBB has been confirmed in the striatum of patients through several methods, including erythrocyte extravasation, perivascular hemosiderin, and leakage of various serum proteins outside UEA-staining vessel walls.^204^ In the SN of 6-hydroxydopamine (6-OHDA) induced PD rat model, increased permeability of BBB was observed by both gadolinium-enhanced MRI and immunohistochemistry after injection of 6-OHDA into the medial forebrain bundle for 2 days.^205^ However, this increased permeability was restored after 1 week of injection. At the same time, decreased immunoreactivity of tyrosine hydroxylase was observed in the SN of 6-OHDA rats at both 2 days and 4 weeks, while iron deposition was observed at 1 and 4 weeks,^205^ suggesting that alteration of BBB might contribute to nigral iron deposition in the PD rat model. In the high-iron and PD mice models, knockout of the transcription factor NF-E2-related factor 2 (Nrf2) prevented iron deposition in the SN and striatum.^206^ The mechanism is likely achieved by decreasing the level of FPN1 on microvascular endothelial cells, which hinders the process of iron entry into the brain.

**Table 1:** Brain regional metal ions distribution in neurodegenerative diseases

Disease	Brain regions	Metal ions level	Methods	Source and references
PD	SN, RN, CPu, GP, PFC frontal, posterior parietal and insular cortices, CSF	Iron ↑	QSM, SWI, MRI, Perl’s staining	PD patients^20,197,215,216^
	mPFC, ACC, Hip, precuneus, AG, SMA, and MOG	Iron ↑	QSM	PD patients with anxiety^213^
	SN, CN, GP, OC, and TC	Iron ↓	MRI, ICP-MS	Drug-naive PD patients^197^ PD patients^217^
	SN, GP, Putamen, AMG, ventricles, Hip, MPO, LS, and VMH	Manganese ↑	ICP-MS, LA-ICP-MS Mn-enhanced MRI	6-OHDA-induced rat model^403^ DJ-1 knockout PD mice with MnCl_2_ treatment^406^
	CSF, GP, Putamen, and AMG	Copper ↑	ICP-MS	PD patients,^442^ 6-OHDA-induced rat model^403^
	SN, LC, MCX, CG, PVC, Hip, MED, and MTG	Copper ↓	SRXFM, ICP-MS	PD patients^445,446^
	CSF	Zinc ↓	ICP-MS, AAS, ICP-AES	PD patients^513^
	GP, Putamen, AMG, and SNpc	Zinc ↑	ICP-MS ZnAF-2DA	6-OHDA-induced rat model^403^ Paraquat-treated rat^521^
AD	cerebral cortex, Hip, basal ganglia, neocortical regions, deep gray matter, putamen, and temporal lobe	Iron ↑	3-T MRI, LA-ICP-MS	AD patients^218,222,223^
	GP	Iron ↓	AAS	AD patients^221^
	CSF	Manganese ↓	ICP-MS	AD patients^418^
	Cortical tissue, brain stem	Copper ↑	ICP-MS	AD patients^464^ APP^NL-G-F^ knock-in mice^463^
	Cortex, Hip, AMG	Zinc ↑	multi-element PIXE	AD patients^526,527^
ALS	left precentral gyrus, thalamus, MC, SN, GP, and RN, CSF	Iron ↑	QSM, ICP-MS	ALS patients^47,238^
	CSF	Manganese ↑	ICP-MS	ALS patients with disease duration less than 19 months^243^
	CSF	Copper ↑	ICP-MS	ALS patients with disease duration less than 19 months^243^
	CSF	Zinc ↑	ICP-MS	ALS patients with disease duration less than 19 months^243^
HD	CPu, GP, CSF	Iron ↑	ICP-MS, QSM, AAS	HD patients^3,46,256,258^
	GP	Iron ↓	ICP-MS	HD patients^259^
	CSF	Manganese ↑	AAS	HD patients^256^
	SN	Manganese ↓	ICP-MS	HD patients^259^
	Putamen, SN, CSF	Copper ↑	ICP-MS, AAS	HD patients^3,256^
	cerebellum	Copper↓	ICP-MS	HD patients^259^
	Putamen, GP and middle frontal gyrus, CSF	Zinc ↑	ICP-MS AAS	HD patients^3,256,259^

The iron distribution in the brain regions is heterogeneous, and selective iron accumulation occurs in several brain regions with aging, such as the SN, caudate putamen, and GP. However, the degree of iron accumulation is particularly severe in the corresponding brain regions of patients with PD. With the development of imaging technology, an increased number of brain regions have been identified to contain abnormal iron levels in PD patients or PD animal models. Furthermore, abnormal accumulation of iron in specific brain regions may not only contribute to motor dysfunction, but also be associated with the non-motor symptoms in PD. In addition to a significant increase of nigral iron levels in all Hoehn and Yahr (H&Y) stages of PD patients without significant difference within stages, compared to healthy, age-matched controls, there was also an observed increase in iron level QSM in the red nucleus in stage II and combined stages III and IV, whereas no significant change of iron levels in caudate putamen, and GP between all stages of PD and controls.^207^ However, another report employing susceptibility-weighted imaging (SWI) has revealed iron depositions in the putamen and GP in idiopathic PD patients, suggesting an association with mitochondrial impairment.^197^ Accumulation of misfolded α-synuclein is one of the most important factors in the pathological development of PD. Intranasal administration of human α-synuclein preformed fibrils (PFFs) was found to cause iron deposition in the SN and GP in a time-dependent manner from 1 to 17 months in the male *Macaca fascicularis*.^208^ Susceptibility MRI data has reported dynamics changes of nigral iron in PD, which are lower before dopaminergic medication and then increase throughout the disease, eventually plateauing at the late stages.^209^ In drug-naive PD patients, lower iron levels were identified in the SN, caudate nucleus, and GP compared to controls, but not in the red nucleus or putamen, however, higher nigral iron were found in drug-treated PD patients compared to controls or drug-naive PD patients.^209^ Notably, PD medications may result in differential association with nigral iron deposition. Higher nigral iron was found to be associated with levodopa usage, while lower nigral iron was correlated with selegiline usage.^209^

Anxiety is a common neuropsychiatric manifestation of PD, and the prevalence of anxiety disorders in PD is higher than that in other chronic neurodegenerative diseases.^210,211^ Anxiety and fear are considered to share the fear circuit, which is composed of the amygdala (AMG), medial prefrontal cortex (mPFC), anterior cingulate cortex, hippocampus, insula, and striatum.^212^ QSM data has revealed that, compared to the healthy controls, increased brain iron accumulation was observed in the fear circuit (including mPFC and anterior cingulate cortex), supplementary motor area, precuneus, angular gyrus, and middle occipital gyrus of PD patients with anxiety, however, increased brain iron accumulation was observed in the parahippocampal gyrus and superior temporal gyrus of PD patients without anxiety.^213^ Specifically, compared to PD patients without anxiety, significant iron deposition was observed in the hippocampus of PD patients with anxiety. Pain is another common non-motor symptom of PD, however, it is still unclear whether iron is involved in PD-related pain. A recent clinical report has found increased iron accumulation in the putamen, caudate, and nucleus accumbens (NAC) of migraineurs compared to the controls, and the degree of iron deposition in NAC could be employed to distinguish the patients with chronic migraine from episodic migraine,^214^ indicating that abnormal iron accumulation in related nuclei may account for the pain of PD. In addition, QSM data has shown increased iron content in the prefrontal cortex and putamen (*p* < 0.05 corrected for multiple comparisons) of individuals with PD compared to controls.^215^ Additionally, within the PD group, higher levels of iron were associated with (1) lower cognitive performance in the hippocampus and thalamus; (2) poorer visual function and higher dementia risk scores in parietal, frontal, and medial occipital cortices; (3) worse motor performance in the putamen. Furthermore, compared to the healthy controls, increased magnetic susceptibility was observed in the frontal, posterior parietal, and insular cortices of PD patients when analyzed by QSM, whereas slightly decreased susceptibility values were observed in the occipital cortex of PD patients.^216^ In addition to iron accumulation in the aforementioned brain regions, our group has previously reported decreased levels of iron in the temporal cortex of postmortem PD patients brains compared to the age-matched healthy controls, which were accompanied by decreased levels of iron-related proteins, including DMT1 (+IRE), TfR1, FPN1, and IRP1.^217^ Conversely, no significant changes in iron levels were observed in the temporal cortex of AD patients. These findings suggest that abnormal distribution of iron may exist in different brain regions in PD and contribute to PD-related symptoms. In addition to the imaging methods mentioned above, transcranial sonography (TCS) is also employed as a non-invasive method for detecting iron deposition in the midbrain. It has been suggested that TCS and MRI parameters should be considered complementary in the detection of iron deposition in PD.^195,196^

#### Brain regional metal ions redistribution in AD

The redistribution of iron also exists in the brain of AD patients. Several brain regions associated with the pathogenesis of AD (Table 1), such as the cerebral cortex, hippocampus, and basal ganglia, exhibit higher levels of iron in AD patients.^28,218–220^ Within the CA1 region of the hippocampus, iron deposition was observed in the stratum molecular-radial and stratum oriens of AD patients, as indicated by LA-ICP-MS results.^199^ However, a reduced iron concentration was observed in the GP of AD patients compared to that in age-matched control participants, which differed from the results in PD patients.^221^ Additionally, higher levels of iron were also observed in the neocortical regions, deep gray matter, and putamen of AD patients compared to healthy controls.^222,223^ Due to the complexity of the cerebral cortex, there was a diverse iron content in AD patients. In comparison to the controls, there were significantly higher correlations between iron concentrations and Aβ plaques as well as tau pathology in the frontal cortex and temporal cortex of AD group,^224–226^ while iron levels remained unchanged in the cingulate cortex, parietal cortex, and entorhinal cortex of AD patients.^226,227^ Importantly, iron deposition in the temporal lobe was closely associated with cognitive decline in AD patients.^222^ It was a common phenomenon that there were significant spatial differences in the distribution of cortical iron in AD patients, which may indicate a redistribution of iron within the cerebral cortex.^224,225,228^ QSM results has shown that cortical iron accumulation is associated with both cognitive decline and cerebral atrophy in AD.^229^ Both ICP-MS and atomic absorption spectrometry (AAS) results showed no significant changes in the level of iron in the CSF of AD patients.^200–202^ However, the iron level in the blood of AD patients varied depending on the methods used. Inductively coupled plasma atomic emission spectrometry (ICP-AES) results showed that serum iron levels were higher in control subjects compared to patients with AD,^203^ which was consistent with the ICP-MS results.^230–232^ AAS results indicated no significant change in serum iron content between the control group and AD patients.^202,233,234^ However, higher serum iron levels were also reported in AD patients.^235,236^

#### Brain regional metal ions redistribution in ALS

As a fatal neurodegenerative disease, ALS is characterized by the loss of motor neurons and muscular atrophy.^237^ Significant iron deposition was observed in the left precentral gyrus and the thalamus, with iron deposition in the thalamus being associated with disease severity in ALS patients (Table 1).^47^ QSM results revealed increased iron levels in the motor cortex, SN, GP, and red nucleus, while decreased iron levels were observed in the white matter of corticospinal tract in ALS patients.^238^ Elevated levels of serum ferritin was also found in ALS patients.^239^ Meta-analysis revealed that ALS patients had lower total iron-binding capacity, and elevated serum ferritin levels were associated with reduced survival as indicated by pooled hazard ratios.^240^ Increased levels of iron and copper were also observed in the blood ALS patients, and disease severity was positively correlated with levels of copper, calcium, cadmium, and lead.^241^ In the cortical region, which is first affected in the ALS, although significantly increased iron was observed in ALS patients by ultra-high field (7 T) MRI, calcium was selectively accumulated at the low myelin borders, indicating the role of calcium in monitoring demyelination in ALS patients.^242^ Increased levels of iron, manganese, copper and zinc were detected in the CSF of ALS patients with a disease duration less than 19 months using ICP-MS.^243^ Furthermore, higher levels of copper, iron, manganese, zinc were observed in the CSF of ALS with spinal onset compared to those with bulbar onset.^243^ Ion chromatography-inductively coupled plasma mass spectrometry has also revealed a potential positive correlation between increased copper in the CSF and genetic ALS.^244^ However, another set of ICP-MS results showed lower levels of copper in the CSF of ALS patients.^245^ Additionally, elevated levels of copper, iron and zinc were observed in the muscle tissue of SOD1^G93A^ mice.^246^ Although an increased expression level of Cp was observed in the CSF of ALS patients, the ferroxidase activity of Cp was comparable between ALS patients and controls, indicating impaired function of Cp in ALS.^247^ With high-resolution ICP-MS, significantly higher levels of metals, including manganese, copper, zinc et al., were observed in the CSF of ALS patients, and the levels of these metal ions were also found to be higher in the CSF of ALS patients than in their blood,^248^ which may be attributed to the disruption of BBB permeability.^249^

The spinal cords of ALS patients were also observed to have increased levels of manganese through radiochemical neutron activation analysis.^250^ Metabolomic analysis of the CSF revealed that copper and manganese were the most significant redox metals for ALS patients.^251^ Increased levels of zinc and decreased levels of magnesium were observed in the brain of ALS transgenic SOD1^G93A^ mice, particularly in the motor cortex, the prelimbic, and infralimbic areas of the frontal cortex, and nucleus of the vertical limb of the diagonal band.^252^ After analyzing the metal levels in the erythrocyte using ICP-MS, it was found that zinc was associated with a decreased risk of ALS, while cadmium and lead were associated with an increased risk of ALS.^50^ Increased levels of zinc were also observed in the white matter of several mutant SOD1 mice.^253^ Dysregulation and exposure to metal ions, including manganese and zinc, among others, in early life have been shown to contribute to ALS.^252^ A recent ecological study conducted in the province of Ferrara, northern Italy found a strong and direct correlation between ALS density and copper concentrations in air pollutants, which correlation was higher in the urban sector, particularly among women in the overall population and urban population, indicating a potential toxic effect of copper on ALS.^254^

#### Brain regional metal ions redistribution in HD

As an autosomal-dominant neurological condition, HD is characterized by a gradual deterioration of psychiatric, cognitive, and motor functions. The pathogenesis of HD is caused by an abnormal expansion of the polyglutamine repeat sequence at the N-terminus of the mutant Huntingtin (HTT) protein, which ultimately leads to atrophy in key brain regions, particularly the striatum and cerebral cortex.^255^ After analyzing frozen postmortem brain tissue using inductively coupled plasma spectroscopy, increased levels of total iron were observed in the striatum (putamen and/or caudate nucleus), as well as elevated copper levels in the putamen and SN of HD patients in 1991 (Table 1).^3^ QSM results showed that iron accumulation in the basal ganglia, including the pallidum, putamen, and caudate of both premanifest and symptomatic HD patients, and iron accumulation in both the putamen and caudate was significantly associated with the severity of HD.^46^ Elevated levels of copper, manganese and zinc were observed in the CSF prior to alterations in canonical biomarkers of HD, and elevated iron was also observed in the CSF of manifest HD patients.^256^ Additionaly, increased levels of iron and zinc were detected in the blood of individuals with HD.^257^ QSM revealed increased iron concentration in the striatum and GP of patients who were closer to onset or had early HD, which were directly correlated with their HD CAG-age product score and brain atrophy.^258^ However, decreased iron levels in the GP were reported in the brain tissues from nine HD cases using ICP-MS.^259^ Meanwhile, decreased copper levels were observed in the cerebellum, while decreased manganese levels were found in the SN. Additionally, increased zinc concentrations were detected in the putamen, globus pallidus, and middle frontal gyrus by ICP-MS. Furthermore, elevated levels of Cp, a copper transport protein, were identified in the hippocampus, parietal cortex, and SN of HD cases.^260^

### Cellular iron dysregulation

#### Cellular iron dysregulation in PD

**Fig. 3:**
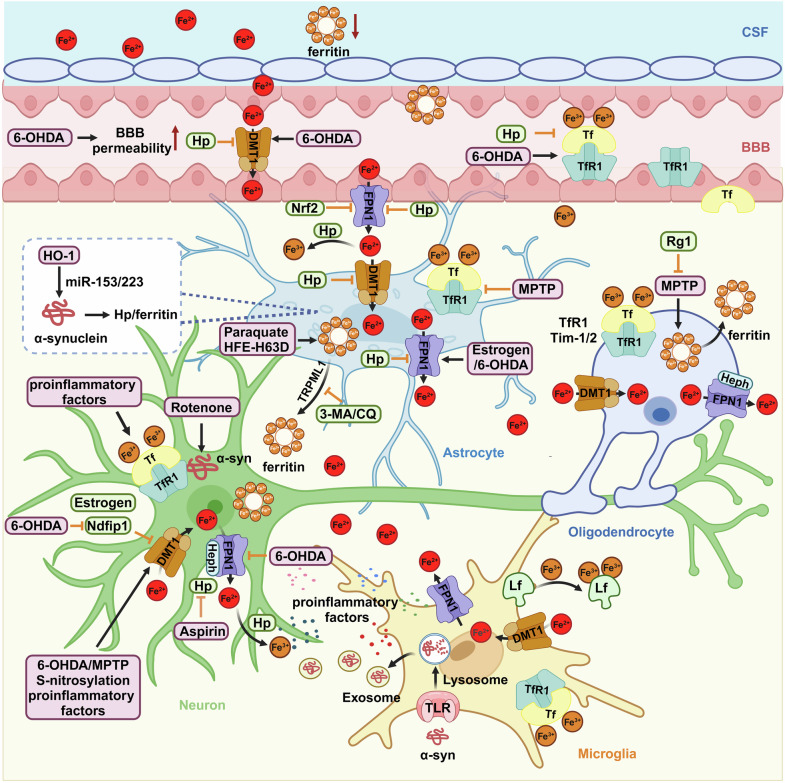
Iron dysregulation among different cells occurs in the state of PD. The increased iron level and decreased ferritin level have been found in the CSF under the state of PD. The increased permeability of BBB facilitates the import of iron through TfR1 by microvascular endothelial cells, which is then released into the brain through FPN1. Once iron crosses the BBB, astrocytes absorb it and mediate its transfer to neurons. Astrocytes have shown a neuroprotective role in PD through the secretion of hepcidin and ferritin, which not only decreases brain iron load by regulating FPN1 on microvascular endothelial cells but also controls iron import by regulating iron-related proteins. With exposure to α-synuclein, iron, or neurotoxins, astrocytes also exhibit an iron-releasing phenotype that is detrimental to neighboring neurons. Microglia are the most susceptible neuronal cells to ferroptosis. Upon stimulation, activated microglia release proinflammatory factors, iron, as well as α-synuclein, which interact and exacerbate dopaminergic neurotoxicity. On the other hand, activated microglia can synthesize lactoferrin and protect vulnerable dopaminergic neurons. Oligodendrocytes harbor the highest concentration of iron in the CNS, which can secrete a ferritin heavy chain and protect dopaminergic neurons from oxidative stress. Abnormal import and export of iron-mediated by iron-related proteins cause iron deposition in nigral dopaminergic neurons, resulting in the loss of dopaminergic neurons in PD. This figure was created with BioRender.com/f23j195

Enrichment analyses for genes associated with cortical iron deposition in PD patients and expression-weighted cell-type enrichment analysis have shown that the top 20% of up-weighted genes (324 genes) related to brain iron were significantly enriched in astrocytes, glutamatergic and GABAergic neurons, as well as oligodendrocyte precursor cells, whereas the top 20% of down-weighted genes (214 genes) showed significantly increased expression in GABAergic and glutamatergic neurons.^216^ Although PD is also associated with an increase in NTBI concentration in the brain,^261^ in the cultured cells exposed to ferric ammonium citrate, astrocytes, microglia, and neurons are all able to safely store NTBI without significant changes in viability, suggesting that dysregulation of iron transport or storage may mainly contribute to iron-induced toxicity in PD (Fig. 3).^87^

##### Neuron

**Fig. 4:**
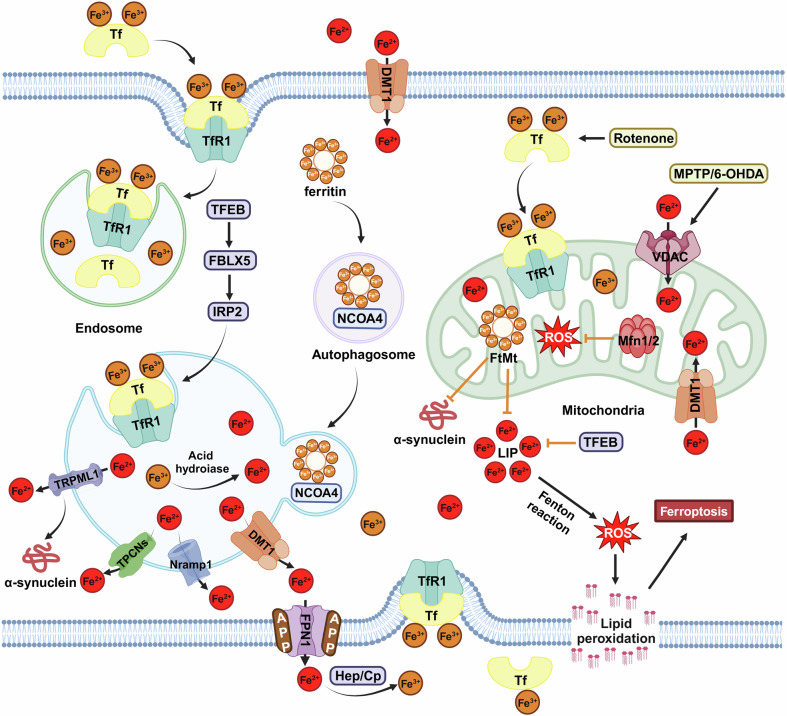
Iron dysregulation in the lysosome and mitochondria of PD. The Tf-TfR system mediates the uptake of Fe^3+^ and DMT1 mediates the uptake of Fe^2+^, which are the two major pathways for the neuronal iron influx. FPN1 is responsible for exporting Fe^2+^ with the help of APP, Cp, or hephaestin (Hep). Endosome contains abundant Fe^3+^ through Tf-TfR1 uptake, while autophagosome contains ferritin bound to NCOA4, which are the main source of iron in lysosomes. In the acidic and reducing environment of the lysosome, Fe^3+^ is reduced to Fe^2+^ and released into the cytosolic through DMT1, TRPML1, Nramp1, or TPCNs. The overexpression of TFEB can upregulate the synthesis of TfR1 through the FBXL5-IRP2 pathway and increase the localization of TfR1 in lysosomes, thereby facilitating the import and temporary storage of iron in lysosomes. VDAC, Tf-TfR2, and DMT1, all of which are located on the outer mitochondrial membrane, are responsible for the iron transport across the outer mitochondrial membrane. The transport of iron across the inner mitochondrial membrane is mediated by Mfrn1/2. In the mitochondrial matrix, iron is stored in FtMt. Rotenone induces an increased level of Tf, while MPTP or 6-OHDA induces increased levels of VDAC in mitochondria. Additionally, excessive iron induces the generation of ROS and lipid peroxidation through the Fenton reaction, further leading to ferroptosis. However, overexpression of TFEB or FtMt has the ability to suppress α-synuclein aggregation and decrease the cellular labile iron pool. All these processes contribute to the nigral dopaminergic neuronal death. This figure was created with BioRender.com/u48z218

**Fig. 5:**
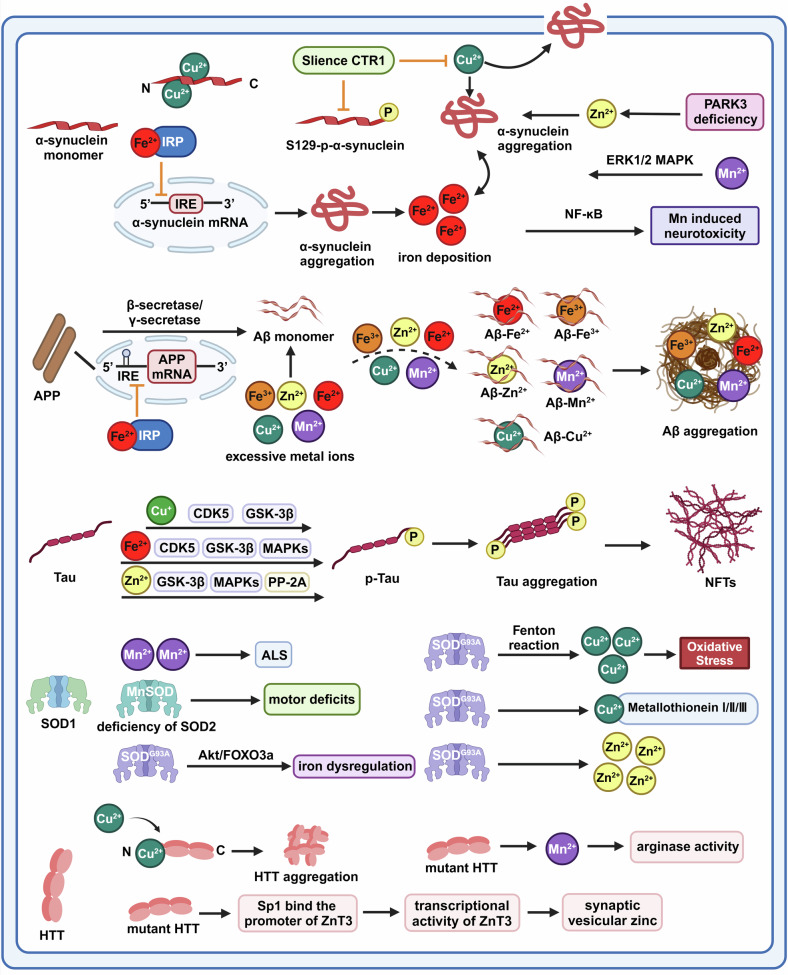
Crosstalk between dysregulation of metal ions and pathological proteins in neurodegenerative diseases. Excessive iron affects α-synuclein aggregation, while misfolded α-synuclein accelerates iron deposition. There are two copper-binding sites in the N-terminus of α-synuclein that increase its aggregation. Silencing CTR1 can decrease the phosphorylation and aggregation of α-synuclein. PARK9 deficiency induces increased zinc levels and α-synuclein aggregation. Manganese can indirectly stimulate the expression of α-synuclein through activating the ERK1/2 MAPK pathway. On the other hand, α-synuclein aggregation could enhance manganese-induced neurotoxicity through the NF-κB pathway. The binding of IRP to iron inhibits the IRE, which increases the expression of APP. APP can be cleaved by β- and γ-secretase in early endosomes to form Aβ in the amyloidogenic pathway. On one hand, excessive metal ions induce increased levels of Aβ monomers; on the other hand, metal ions can bind to Aβ and promote its aggregation. Iron, copper, and zinc enhance tau phosphorylation by activating CDK5, GSK-3β, or MAPKs and inactivating PP-2A activity to accelerate the formation of NFTs. Manganese exposure promotes the development of ALS. Additionally, partial deficiency of SOD2 significantly exacerbates motor deficits. Iron dysregulation induced by SOD1^G93A^ is mediated by impairment of the Akt/FOXO3a signaling pathway. Meanwhile, increased levels of copper, zinc, and metallothioneins-I/II/III are observed in SOD1^G93A^ mice. Copper has the ability to bind with the N-terminus of HTT proteins, thereby promoting their aggregation. Mutant HTT can lead to a deficiency in neuronal manganese, which affects arginase activity. Additionally, mutant HTT inhibits the binding of Sp1 to the promoter of the ZnT3 gene, resulting in decreased levels of synaptic vesicular zinc in the hippocampus, cortex, and striatum. This figure was created with BioRender.com/q53d457

Selective loss of nigral dopaminergic neurons is one of the most common pathological hallmarks in PD, and increasing evidences have proven that iron deposition induced by dysregulated iron-related proteins contributes to the aforementioned process by inducing iron deposition. DMT1 is the most well-investigated iron transport involved in neuronal iron dysregulation in PD. In the substantia nigra pars compacta (SNpc) of postmortem tissue from PD patients, both increased DMT1 labeling and iron accumulation were observed in the remaining neuromelanin-positive dopaminergic neurons compared with age-matched control.^262^ As DMT1 labeling also appeared in activated microglia, the increased expression of DMT1 (+IRE) and iron deposition in the SNpc of postmortem tissue from PD patients may be the results of elevated levels of DMT1 in the remaining dopaminergic neurons and activated microglia.^262^ Furthermore, increased DMT1 in the SN has also been observed in animal models of PD, including MPTP-induced mice model,^262^ 6-OHDA induced rat model,^263^ rotenone-induced mice model,^264^ PD mice with overexpression of human α-synuclein-A53T in the SNpc, PD transgenic mice with overexpression of human α-synuclein-A53T,^265^ and related PD cell models. Nedd4 family–interacting protein 1 (Ndfip1) is an adapter for the Nedd4 family of E3 ligases, which can bind to DMT1 in response to iron exposure and facilitate the degradation of DMT1 through the ubiquitin-proteasome system.^266^ In our previous study, decreased Ndfip1 was observed in 6-OHDA-induced PD rat and cell models, and overexpression of Ndfip1 downregulated the level of DMT1(+IRE), reduced iron accumulation, and attenuated neurotoxicity induced by 6-OHDA.^267^ The iron import ability of DMT1 could be enhanced by its *S*-nitrosylation, which may contribute to the loss of dopaminergic neurons, and *S*-nitrosylated DMT1 has also been observed in the SN of postmortem tissue from individuals with PD, as well as in PD mice induced by lipopolysaccharide.^268^ Recently, it has been found that increased DMT1 is induced by HMGB1-mediated inflammation, which contributes to dopaminergic neurodegeneration in the early stage of PD.^269^ Notably, mutations in DMT1, which impaired the iron transport, have shown neuroprotection for nigral dopaminergic neurons in both MPTP- and 6-OHDA-induced PD mice models.^262^ Also, the inhibition of *S*-nitrosylation of DMT1 by either the NO synthase inhibitor l-NAME or the DMT1-selective blocker ebselen was able to prevent lipopolysaccharide-induced loss of dopaminergic neuron.^268^ All these evidences suggest that regulating DMT1 through genic or pharmacologic methods may be an effective strategy for PD prevention or treatment. The Tf-TfR1 system is the major pathway for importing ferric iron from the extracellular environment. In cultured neurons, treatment with Tf was found to correct iron accumulation, and in the MPTP-induced PD mice model, Tf treatment ameliorated iron accumulation and improved iron deficits.^270^ Our recent report showed that α-synuclein-A53T and iron function as a toxic couple, inducing cell senescence in both mice and cell models of PD, which precedes the loss of nigral dopaminergic neuron.^23^ Additionally, reducing the iron load through DFO or knockdown of TfR1 significantly improves the phenotypes of cell senescence induced by α-synuclein-A53T (Figs. 4, 5).

As the sole known iron exporter, decreased FPN1 also contributes to the accumulation of iron in nigral dopaminergic neurons in PD.^106^ Recently, significant decreases in both mRNA and protein expression of FPN1 have been observed in the brains of PD patients compared with age-matched controls.^271^ In primary cultured ventral mesencephalic neurons and MES23.5 dopaminergic cells with 6-OHDA treatment, decreased FPN1 but not hephaestin contributed to the iron accumulation.^272^ Both hepcidin and Cp function as ferroxidase, which converts the highly toxic ferrous iron into nontoxic ferric form and collaborates with FPN1 to facilitate iron export.^273^ In the N27 dopaminergic neuronal cells, the knockdown of hepcidin significantly upregulates FPN1 and decreases intracellular iron, which also attenuates 6-OHDA-induced apoptosis.^274^ In the primary cultured cortical neurons, increased hepcidin by adenovirus resulted in inhibited iron uptake and release, as well as decreased levels of iron transport proteins including TfR1, DMT1 (+IRE), DMT1 (−IRE), and FPN1.^62^ Conversely, knockdown of hepcidin yielded opposite results. In the 6-OHDA-induced PD rat model, acupuncture was found to reduce iron accumulation in the SN by balancing the ratio of DMT1/FPN1, thereby protecting nigral dopaminergic neurons.^275^ In PC12 cells, aspirin has been reported to increase the expression of FPN1 by inhibiting hepcidin through the IL-6/JAK/STAT3 pathway, which promotes FPN1-mediated iron release and reduces neuronal iron levels.^276^ All these evidences suggest that modulating FPN1 is promising manner to reduce iron level in nigral dopaminergic neurons. The level of Cp in the serum is suggested to be a potential biomarker for PD, but it appears to vary across different reports. A significantly lower level of Cp in the serum was observed in PD patients using I-FP-CIT-SPECT in 2017^277^; however, no significant change of Cp in the serum was reported in PD patients in 2018.^278^ Interestingly, a study conducted in 2020 found higher serum Cp levels specifically among females with PD, which were associated with increased impulsivity.^279^ Therefore, it may be necessary to consider using more sensitive detection methods and increasing the number of samples from PD patients to confirm changes in Cp levels. In the Cp knockout mice, a loss of approximately 30% nigral dopaminergic neurons was observed at the age of 5–6 months, accompanied by nigral iron deposition, and peripheral administration of Cp could attenuate both the nigral iron deposition and neurodegeneration in MPTP-induced PD mice model.^280,281^ In a clinical trial, PD patients with higher levels of Cp-ferroxidase activity in their serum and CSF were found to be more sensitive to iron chelator deferiprone treatment, which showed greater reduction in UPDRS scores and nigral iron levels after 6 to 12 months of therapy,^282^ suggesting that targeting Cp may have the potential to improve iron accumulation in nigral dopaminergic neurons.

##### Microglia

Microglia, as the most efficient iron-absorbing glial cells, play an essential role in maintaining iron homeostasis.^87,88^ Additionally, microglia are susceptible to ferroptosis, which is dependent on the vesicle trafficking gene *SEC24B*; however, their transcriptional state changes significantly after iron overload.^90,283^ Compared to astrocytes and neurons, human-induced pluripotent stem cell-derived microglia have been recognized as highly responsive to iron and susceptible to ferroptosis. Additionally, iron overload resulted in a PD-like shift in the transcriptional state of microglia.^90^ Microglia activation has been proposed to be an early event in PD. Idiopathic rapid eye movement sleep behavior disorder (iRBD) is considered a prodromal stage of α-synucleinopathies, such as PD, with a conversion rate to α-synucleinopathies reaching 41% within five years.^284^ Positron emission computed tomography has shown increased microglia activation in the SN of patients with iRBD, which is accompanied by reduced dopaminergic function in the putamen.^285^ In the SN of rotenone-induced PD mice model, microglial-mediated neuroinflammation was found to be triggered by gut microbiota-induced elevation of CXCL1.^264^ In the rat neuron-microglia-astrocyte, iron induces selective toxicity in dopaminergic neurons, and microglia activation exacerbates the neurotoxicity of dopaminergic neurons.^286^ Proinflammatory factors released by activated microglia, such as interleukin (IL)-6, IL-1β, and TNF-α, have been found to upregulate the expression of iron import-related proteins, including DMT1, IRP1, and TfR1, while downregulating the expression of the iron export protein FPN1, which leads to neuronal iron accumulation.^286–289^ Conversely, iron overload further enhances microglial activation and proinflammatory factor release, creating a vicious circle. LRRk2 is indicated to be downstream of cellular proinflammatory signals, and it has been found to be activated in the microglia of postmortem PD tissue. In iPSC-derived microglia from PD patients carrying LRRK2-G2019S, Tf recycling was found to be dysregulated under proinflammatory conditions, and this dysregulation was observed in the lysosomes located near the nucleus.^290^ Additionally, both iron and ferritin accumulation were found in the inflammatory microglia of LRRK2-G2019S knock-in mice.^290^ Microglia can interact directly with α-synuclein, thereby promoting the occurrence and development of PD. Microglia can engulf α-synuclein released by neurons and degrade it through autophagy. When microglia are unable to degrade intracellular α-synuclein, they can release α-synuclein through exosomes, promoting the transmission of α-synuclein.^291–293^ Furthermore, aggregated α-synuclein can inhibit microglial phagocytosis and autophagy, while impaired microglial autophagy can promote inflammation, aggravating the accumulation of α-synuclein and loss of nigral dopaminergic neurons.^292,294,295^ In male *Macaca fascicularis*, nigral iron deposition induced by nasal mucosal delivery of human PFFs was specifically localized in microglia rather than dopaminergic neurons and other types of glial cells.^208^ However, increased levels of Tf, TfR1, TfR2, and FPN1 were observed in the nigral dopaminergic neurons. In the 6-OHDA induced PD rat model, microglia activation, L-ferritin and iron deposits were found to be co-localized in the SN.^205^ Specifically, only activated microglia can synthesize Lf, which is able to bind with iron and protect vulnerable dopaminergic neurons, and an increase in iron overload could enhance the release of Lf by activated microglia.^92,296,297^

##### Astrocytes

Astrocytes have attracted increasing attention in PD.^298^ In both rotenone- and 6-OHDA-induced PD mice, overexpression of hepcidin using a virus-based strategy has been found to suppress major pathologies of parkinsonism and motor deficits,^299^ suggesting that further studies could explore the protective role of astrocyte-derived hepcidin in PD. In a co-cultured system of primary astrocytes and MES23.5 cells, it was observed that ferritin released by astrocytes entered MES23.5 cells and protected them against MPP^+^-induced ferroptosis and neurotoxicity.^85^ Estrogen is considered to play a neuroprotective role in women with a lower risk of PD, which displays a different regulatory role in iron metabolism between astrocytes and neurons.^300^ Estrogen increases the levels of FPN1 and DMT1 via HIF-1α in astrocytes, while decreasing DMT1 and increasing FPN1 in the neurons, suggesting that estrogen promotes the iron transport in astrocyte, which may transfer iron to other tissues such as blood vessels, thereby reducing neuronal iron accumulation.^300^ The homeostatic iron regulator (HFE) gene variant (H63D in humans, H67D in mice) acts as a disease-modifier in PD, while also playing an important role in regulating cellular iron uptake. With the treatment of paraquat to induce PD-like phenotype, H67D HFE mice showed an increased level of ferritin light chain and exhibited resistance to paraquat-induced nigral neuronal toxicity.^301^ Furthermore, increased levels of ferritin light chain and activation of Nrf2 were observed in the H67D-HEF astrocytes following paraquat treatment, which provided protection against paraquat-induced neurotoxicity for both astrocytes and neighboring neurons.^302^ On the other hand, astrocytes also have a negative effect on neuronal iron accumulation in PD. When exposed to both α-synuclein and iron, the ratio of hepcidin to ferritin decreased in primary culture astrocytes, indicating an iron-releasing phenotype that may be detrimental to neighboring neurons.^303^ Both DMT1 and TfR1 are responsible for the iron uptake in astrocytes.^304^ In primary cultured astrocytes, increased iron transporters, such as DMT1( + IRE) and FPN1, IRP1 and ferritin light chain were observed after 6-OHDA treatment, indicating that 6-OHDA promotes the trafficking of iron in astrocytes, which may contribute to the accumulation of iron in neurons.^263,305^ In primary cultured astrocytes from IRP2 knockout mice, MPP^+^ increased the levels of ferritin and DMT1(-IRE) while decreasing the level of TfR1, which resulted in an elevated iron level in the astrocytes and may reduce their ability to buffer iron from neuron, thereby contributing to nigral iron deposition and neuronal apoptosis.^306^ The accumulation of iron gradually increases in astrocytes with age, which may lead to the development of neurodegenerative diseases.^81,307^ Heme oxygenase-1 (HO-1) is a 32 kDa enzyme that controls the cellular heme catabolism, leading to the production of carbon monoxide, iron, and biliverdin, which process is involved in gliopathy and contributes to neuronal vulnerable to oxidative injury in PD.^308^ In the GFAP.HMOX1 transgenic mice aged between 8.5 and 19 months, overexpression of heme oxygenase-1 (HO-1) in astrocytes induced parkinsonism-like phenotypes, such as nigrostriatal hypodopaminergia, altered gait, locomotor incoordination, reduced olfaction, increased α-synuclein by downregulating miR-153 and/or miR-223, iron deposition in the basal ganglia, mitochondrial damage, and oxidative stress.^309–311^

##### Oligodendrocytes

Iron dyshomeostasis in the oligodendrocytes is also involved in PD; however, the understanding of this process is limited. In the SN of PD patients, although there is no significant change in iron levels in ferritin-positive oligodendrocytes, the level of ferritin in these cells was reduced by almost half compared to that of non-neurodegenerative control individuals.^93^ In the striatum of MPTP-induced PD mice model, ginsenoside Rg1 has been reported to regulate iron balance by increasing the level of ferritin heavy chain and decreasing the level of ferritin light chain, which plays a positive role in protecting oligodendrocytes against lipid peroxidation stress.^312^ Meanwhile, ginsenoside Rg1 also revealed a neuroprotective effect on nigral dopaminergic neurons in the MPTP-induced PD mice model.^312^ Considering the highest concentration of iron in oligodendrocytes within the CNS, it is worth exploring the contribution of iron dysregulation in oligodendrocytes to PD in further studies.

##### Subcellular

Lysosome damage or dysfunction has been implicated in the pathology of PD, mainly focusing on an autophagy-lysosome pathway. However, as a major cellular iron storage organelle, there is a large amount of ferrous iron in the lysosome (Fig. 4). Nevertheless, an overload of ferrous iron could trigger oxidative stress in the cytosolic and cytotoxicity. Genetic variations in LRRK2 have been implicated in their association with PD, which also participates in lysosomal functions. LRRK2 is capable of phosphorylating a specific group of Rab GTPases, such as Rab8a and Rab29, which are responsible for the spatiotemporal regulation and correct transportation of vesicles.^313,314^ In iPSC-derived inflammatory microglia with mutant LRRK2-G2019S, Rab8a was sequestered, leading to lysosome damage and mistrafficking of Tf into lysosome closer to the nucleus, resulting in iron accumulation.^290^ Ferritinophagy functions as the exclusively identified mechanism for the liberation of iron bound to ferritin. Aggregated α-synuclein can bind and inhibit the v-SNAREs, which blocks fusion between lysosomes and autophagosomes, inhibiting the release of iron from ferritin in PD.^315^ Visual symptoms related to retina degeneration are prominent in PD, and iron dyshomeostasis has been indicated to be involved in the degeneration of the retina.^316,317^ Both in vivo and in vitro evidences has shown that α-synuclein suppresses the process of ferritinophagy and causes accumulation of iron-rich ferritin in the retinal pigment epithelium, which may be responsible for retinal iron dyshomeostasis and PD-associated retinal degeneration.^317^ As the autophagy-lysosome pathway is the primary pathway for clearing abnormal aggregated α-synuclein, and has been implicated as a promising strategy for PD prevention or treatment,^318^ we have attempted to identify a selective autophagy, which can not only degrade α-synuclein but also inhibit ferroptosis in our recent study.^108^ TFEB-mediated autophagy not only promotes the clearance of aggregated α-synuclein but also maintains cellular labile iron at a low level and prevents ferroptosis.^108^ Iron treatment was also found to promote the aggregation and transmission of α-synuclein through TFEB-mediated autophagosome-lysosome fusion.^319^ TRPML1 is a lysosomal release channel for both calcium and iron. It has been reported that activation of TRPML1 could facilitate the maturation of autophagosomes containing α-synuclein, increase the lysosomal exocytosis, and protect dopaminergic neurons from α-synuclein toxicity.^320,321^ Therefore, the activator of TRPML1, such as Artemisia argyi Levl. et Vant., has been suggested to be neuroprotective in PD.^322^ In the postmortem striatum of sporadic PD patients, the level of microglial Nramp1 was found to be significantly increased, accompanied by iron deposition and α-synuclein aggregation.^323^ Under conditions of iron overload, functional Nramp1 is involved in the degradation of α-synuclein aggregates with lysosomal cathepsin D in microglia.^323^

As ROS is mainly produced in mitochondria, which induces oxidative stress and damages dopaminergic neurons, mitochondria dysfunction is considered a causative factor of PD.^324^ Mitochondria are also an important organelle involved in iron metabolism, and dysregulation of mitochondrial iron has attracted increased attention in PD (Fig. 4).^111^ Disruption of mitochondrial iron in PD is mainly caused by the dysregulation of proteins related to mitochondrial iron. Although the mRNA levels of three forms of VDAC (VDAC1/2/3) and TH were significantly decreased, the mRNA ratios between the three forms of VDAC to TH were increased in the SN of PD patients.^21^ Meanwhile, increased VDAC was observed in MPTP- or 6-OHDA-induced PD models.^325,326^ Although VDAC-mediated mitochondrial iron transport has not been directly detected in PD, increased VDAC levels may lead to mitochondrial iron overload, thereby inducing mitochondrial dysfunction in PD. In the rotenone-induced PD cell and rat models, increased Tf levels were found in the nigral dopaminergic neurons, particularly accumulating in the mitochondria.^327^ In the SN of PD patients, increased levels of Tf and oxidized Tf were observed, which proteins were found to co-localize with TfR2 on the mitochondrial of remaining nigral dopaminergic neurons, suggesting that the Tf-TfR2 mediated mitochondrial iron transport system is disrupted in PD, which may be involved in the development of this disease.^327^ Recently, DMT1 has been identified as maintaining the mitochondrial membrane potential. Knockdown of DMT1 was found to enhance the activity of mitochondrial complex while decreasing the activity of complex III, thereby reducing erastin-induced ferroptosis.^328^ In the *C. elegans* model of AD, the knockdown of mitochondrial iron importer mitoferrin was found to decrease the concentration of mitochondrial iron and levels of ROS.^329^ In *Drosophila*, overexpression of the mitochondrial iron importer mitoferrin, or knockdown of the iron binder Fer2HCH, changes iron homeostasis in the mitochondrial, which rescues phenotypes associated with PINK1 loss-of-function, including mitochondrial morphology defects, reduced mitochondrial aconitase activity, and flight deficits.^330^ These findings indicate that targeting mitoferrin or Fer2HCH and modulating mitochondrial iron may be a potential strategy to improve PD. As an important iron storage protein in the mitochondrial, FtMt is expressed in nigral dopaminergic neurons, which has been proven to protect mitochondria from oxidative stress. Overexpression of FtMt was found to suppress α-synuclein expression, sequester iron in the mitochondrial, decrease the cellular labile iron pool, and combat oxidative stress induced by H_2_O_2_ or 6-OHDA.^331,332^ Furthermore, overexpression of FtMt was found to suppress the increase in labile iron pool and inhibit mitochondrial damage, thereby attenuating the Parkinsonian phenotype induced by MPTP in mice.^333^

#### Cellular iron dysregulation in AD

AD is characterized by two pathognomonic protein aggregates, including extracellular Aβ plaques and intracellular tau neurofibrillary tangles. Abnormal iron deposition is involved in the pathological progress of AD, leading to excessive iron accumulation in cells and plaques, such as neurons, glia, as well as Aβ plaques and neurofibrillary tangles.^29,334,335^ It has been reported that redox-active iron is co-localized with senile plaques and neurofibrillary tangles in the hippocampal tissue of AD, and redox-active iron not only participates in on-site oxidation but also catalyzes H_2_O_2_-dependent oxidation.^29^ Chelated removal of metal can abolish the H_2_O_2_-dependent oxidation catalyzed by senile plaques and neurofibrillary tangles, while incubating of iron or copper after the above process can re-induce the catalytic redox reactivity by the lesions.^336^ Recently, with a label-free and nanoscale chemical imaging by synchrotron X-ray spectromicroscopy, higher levels of iron was observed in the amyloid plaques of human AD brain tissue, and this iron appeared to be chemically reduced and in a low-oxidation state.^35^ Cp plays a crucial role in converting ferrous iron to ferric iron. In the AD brains, although the level of Cp was significantly increased in the neuropil, the immunoreactivity of Cp in the neurons was similar to that in the age-matched controls, implying the important role of Cp in the accumulation of redox-active iron in neurons of AD.^337^

APP, a major source of Aβ in AD, can be cleaved by α- and γ-secretase at the plasma membrane to form soluble APP α (sAPPα) through non-amyloidogenic pathway.^338^ After endocytosis, APP can be cleaved by β- and γ-secretase at the early endosomes to form Aβ through an amyloidogenic pathway. It has been reported that IRP can bind to the IRE-mRNA of APP, which not only regulates iron homeostasis,^166,339^ but also downregulates the levels of APP, Aβ, and protein aggregation.^340^ However, in the presence of iron accumulation, the levels of APP, Aβ, and protein aggregation can be upregulated. These pieces of evidence establish a link between IRP-mediated iron homeostasis and AD. APP has been reported to function as an analogous iron-exporting chaperone for neurons and other types of cells, playing an essential role in stabilizing FPN on the cell surface and supporting the export of iron from neurons.^341,342^ In the APP knockout mice, decreased FPN was observed in the brain, and age-dependent iron elevation was observed in both the brain and liver, which correlated with increased ferritin levels and decreased TfR1 expression.^343^ Both genetic and pharmacological methods have demonstrated that the endocytotic amyloidogenic processing of APP reduces iron export by disrupting FPN stabilization on the cell surface, leading to cellular iron retention.^344^ In contrast, the preferential non-amyloidogenic processing of APP on the cell surface enhances FPN stabilization and decreases neuronal iron levels. In the rat primary cortical neurons, iron overload significantly increased the non-amyloidogenic carboxy-terminal fragment α (CTFα) derived from α-secretase cleavage of APP, while decreasing the amyloidogenic products sAPPβ and Aβ. This may be attributed to direct inhibition of β-secretase activity by iron overload.^345^ Additionally, iron overload caused significant alterations in the distribution of neuronal secreted sAPPα, with evidence indicating an increase in cellular levels of sAPPα.^345^ Ferric iron can interact with Aβ and promote the aggregation of both Aβ40 and Aβ42.^346,347^ During the process of fibril formation, Aβ42 can act as a ferritin-specific metallochaperone-like molecule and reduce ferric iron from the ferrihydrite core of ferritin, resulting in a concentration of ferrous iron that is two times higher than that produced by ferritin itself.^348^ X-ray spectromicroscopy and electron microscopy revealed that co-aggregation of Aβ and ferritin leads to the conversion of ferritin into reactive low-oxidation states, which may contribute to increased oxidative stress in AD.^349^ Furthermore, Aβ42 can induce the accumulation of ferric iron within the amyloid aggregates and lead to the reduction of ferric iron into ferrous iron, which process can be enhanced by aluminum.^350^ Increased labile ferrous iron produced during the aforementioned process may induce ferroptosis or enhance oxidative stress, thereby contributing to the development of AD. Tau protein has been suggested as the downstream effector of Aβ toxicity in AD,^351^ which could also promote the transport of APP to the neuronal surface and facilitate FPN-mediated iron export. In the tau-knockout mice injected with Aβ oligomers into the hippocampus, tau ablation was found to reduce the iron accumulation induced by Aβ in the hippocampus.^352^ Iron also has the ability to bind to tau protein, which triggers both tau phosphorylation and aggregation.^353,354^ A positive association between iron deposition and insoluble tau aggregates in the inferior temporal gyrus of AD patients has been confirmed through MR-based QSM and tau-PET.^33^ In the APP/presenilin 1 (APP/PS1) double transgenic mice treated with deionized water containing ferric iron, tau phosphorylation induced by iron overload can be abolished by DFO through cyclin-dependent kinase 5 (CDK5) and GSK-3β pathway.^355^ Meanwhile, high dietary iron could also induce the expression of Aβ and phospho-τ in the hippocampus of both wild-type and APP/PS1 transgenic mice.^356^ The upregulation of HO-1 and its co-localization with tau in the brain is another phenomenon in AD, which can lead to cognitive decline.^357^ In transgenic mice and N2a cells with overexpressing HO-1, long-term overexpression of HO-1 significantly increases tau aggregation, tau phosphorylation, as well as iron accumulation in the brain, and the induction of tau phosphorylation by HO-1 is mediated by iron accumulation.^358^ Insulin resistance is an important hallmark in the brain of AD. In addition to causing aberrant phosphorylation of tau, iron overload also disrupts insulin signaling, as evidenced by decreased levels of tyrosine phosphorylation in insulin receptor β (IRβ), insulin signal substrate 1 (IRS-1), and phosphoinositide 3-kinase p85α (PI3K p85α) in primary cultured neurons, as well as in the brains of iron-overload mice accompanied by impaired learning and memory.^359^

As the inherent immune cells in the CNS, microglia also play an essential role in iron dyshomeostasis in AD, and activated microglia are characterized by both iron accumulation and infiltration of Aβ plaques in AD.^360^ Immunohistochemical analysis revealed that the activation and proliferation of microglia were distributed in Aβ plaques, which were also co-localized with iron, in both AD patients and animal models.^361^ According to the report, excessive iron was found deposited in the activated microglia and plaques, as well as in the mid-cortical layers along myelinated fibers of AD patients using Perl’s histochemical procedure.^225^ Microglia were identified as the main cells containing ferritin, which was associated with senile plaques and blood vessels.^334^ Meanwhile, MRI results showed a higher presence of iron-positive microglia in the hippocampus of AD patients compared to healthy control subjects.^362^ Aβ can upregulate the level of DMT1 and enhance the uptake of NTBI by microglia.^363^ Iron accumulation in microglia induces a rapid transformation from an M2 to a harmful M1 phenotype, exacerbating the process of Aβ-induced microglial IL-1β secretion.^364,365^ In the cingulate cortex of AD patients, Aβ plaques can upregulate hepcidin by increasing microglial secretion of IL-6, which subsequently downregulates FPN and leads to iron deposition in the brain of AD.^366^ Both apo-Tf and holo-Tf regulate the release of iron from endothelial cells at the BBB.^64^ In iPSC-derived astrocytes and endothelial cells, Aβ has been reported to promote iron uptake by astrocytes and increase the level of apo-Tf in the media, which further stimulates iron transport from endothelial cells.^367^ In APP/PS1 mice, the overexpression of Hp in astrocytes significantly decreased iron levels and reduced the formation of Aβ plaques in the cortex and hippocampus, which alleviated oxidative stress and neuroinflammation and enhanced cognitive decline.^368^ Neuroimaging researches have demonstrated that micro- and macrostructural abnormalities in white matter are closely related to the progression of AD.^369–371^ The changes in myelin and oligodendrocytes mainly feature in the abnormal white matter of AD.^372,373^ As the glial cell with the highest iron content, it is worth investigating the role of iron in the aforementioned oligodendrocyte-mediated changes in AD.

#### Cellular iron dysregulation in ALS

In SH-SY5Y cells overexpressing SOD1^G93A^, the mRNA levels of TfR1 and DMT1, as well as the expression levels of mitoferrin 1 and 2, frataxin, and iron-sulfur cluster scaffold protein were all increased, suggesting that dysregulation of iron-related proteins may contribute to the iron imbalance induced by SOD1^G93A^ in ALS.^374^ Abnormal iron is deposited in the motor neurons and glia, accompanied by increased levels of DMT1, FPN, and Cp in the cervical cord,^375^ as well as increased ferritin in glial cells, suggesting the contribution of iron the disease progression of SOD1^G93A^ mice.^375^ The iron dysregulation induced by SOD1^G93A^ is mediated through impairment of Akt/FOXO3a signaling pathway,^376^ and may also be related to damage of iron-sulfur cluster.^377^ Iron accumulation induced by SOD1^G93A^ increases the activity of TNF-α converting enzyme (TACE), which promotes the secretion of TNF-α and induces oxidative stress.^378^ Tf was identified as being localized in Bunina bodies and some of the basophilic inclusions in ALS patients.^379^ The disruption of the blood–spinal cord barrier leads to the accumulation of iron derived from blood and neurotoxic hemoglobin in the spinal cord, which contributes to early motor-neuron degeneration in SOD1^G93A^ mice.^380^ GPX4 depletion and ferroptosis were observed in the spinal cords and brains of transgenic mice models of ALS, including mutant superoxide dismutase 1 (SOD1^G93A^), TDP-43^Q331K^, and C9orf72^500^ mice models, which observations were associated with impairment of Nrf2 signaling pathway and decreased H-ferritin.^381^ Overexpression of GPX4 in SOD1^G93A^ mice can improve locomotor dysfunction and delay disease onset, indicating the involvement of ferroptosis in motor neuronal degeneration in ALS. Additionally, activating Nrf2 could also improve neurodegeneration in the SOD1^G93A^ mice model of ALS.^382^ Decreased Speedy/RINGO cell cycle regulator family member A (SPY1), which was caused by MDM2-mediated ubiquitination degradation, was also involved in ferroptosis in the SOD1^G93A^ mice, and overexpression of SPY1 could inhibit ferroptosis by regulating GCH1/BH4 axis and TfR1, thereby delaying the occurrence and prolonging the survival SOD1^G93A^ mice.^383^

#### Cellular iron dysregulation in HD

In order to investigate the iron changes in the white matter of HD, myelin breakdown was observed with no significant change in iron level in the PreHD stage, and decreased iron was observed in the isthmus in early HD stage using MRI.^384^ Increased iron was found in the putamen, GP and external capsule of premanifest HD individuals from the HD Young Adult Study, indicating that iron accumulation in subcortical structures and the surrounding white matter is an early feature of HD.^385^ Although decreased ferritin levels in the serum of HD patients was reported in 1991,^386^ increased iron was also observed in the striatum and cortex of N171-82Q HD transgenic mice, accompanied by elevated levels of IRP1, Tf, ferritin and TfR.^387^ Furthermore, it was reported that increased nuclear levels of signal transducer and activator of transcription 5 (STAT5) in the brains of N171-82Q mice and 160Q HEK293 cells enhanced the expression of IRP1 and initiated iron deposition.^388^ QSM results showed that iron accumulation in the basal ganglia, including the pallidum, putamen and caudate of both premanifest and symptomatic HD patients, and iron accumulation in both putamen and caudate was significantly associated with the severity of disease.^388^ Iron levels were reported to be inversely correlated with the volume of putamen, globus pallidus and the anterior cingulate; and directly correlated with the volume of cortical structures.^389^ Quantitative susceptibility MRI results revealed higher iron levels in the striatum and GP in individuals with closer-to-onset and early HD, which were directly correlated with the HD CAG-age product score and brain atrophy.^258^ After a longitudinal 1-year follow-up test, a higher rate of iron deposition was found in the caudate and GP, indicating that brain iron might server as a maker for monitoring the progression of HD.^258^ In neonatal R6/2 HD mice, elevated iron uptake caused oxidative stress, energetic dysfunction, and potentiated the disease phenotype.^390^ Additionally, in female YAC128 HD mice, neonatal-iron supplement led to increased striatal degeneration at 1 year old.^391^ Iron accumulation was observed in the perinuclear cytoplasm of striatal neurons in R6/2 HD mice along with decreased IRP1/2 and TfR1 levels and increased FPN expression.^392^ Furthermore, increased ferritin levels were primarily observed in microglia of R6/2 HD mice, suggesting it as an early event of HD.^393^ In the N171-82Q mice model of HD, iron was found to activate microglia and directly stimulate the activity of indoleamine-2, 3-dioxygenase, which catalyzes the initial step of kynurenine pathway.^394^ Additionally, accumulated iron was also observed in the astrocytes of the striatum in the postmortem tissue from HD patients.^395^ In the brain of both 12-week R6/2 and 12-month YAC128 HD mouse models, accumulated mitochondrial iron, increased iron uptake protein mitoferrin 2, and decreased iron-sulfur cluster synthesis protein frataxin were observed, which changes were accompanied by increased lipid peroxidation and mitochondrial dysfunction.^396^ It has been reported that APP is involved in the iron accumulation in HD, with evidence showing that knockdown of APP significantly increases cerebral and striatal iron levels in the YAC128 HD mouse model.^397^ HTT has been reported as an iron-regulated protein,^398^ and the elimination of HTT expression alters brain iron homeostasis in adult mice,^399^ suggesting that HTT may play a crucial role in the dysregulation of iron in HD.

### Dysregulation of other metal ions

#### Dysregulation of manganese

##### PD

Exposure to excess manganese could increase the risk of PD, which was first observed in 1967 among miners who exhibited symptoms of an increasing incidence of dystonia and non-resting tremor.^36^ Additionally, a study in 2003 reported a combined effect of dietary intake of iron and manganese in increasing the risk of PD.^37^ The increased manganese in the whole blood was observed in PD patients without depression compared to both PD patients with depression and the controls.^400^ Recently, it was found that intraperitoneal injection of manganese can lead to Parkinson-like symptoms in C57BL/6J mice, accompanied by disorders in lipid metabolism, oxidative stress, and damage to nigral dopaminergic neurons.^401^ In the MitoPark PD mice model, exposure to manganese was found to impair mitochondrial function and worsen progressive motor deficits.^402^ In 6-OHDA-induced rat models, a significantly increased level of manganese was observed in the SN, GP, putamen, and amygdala over time following 6-OHDA treatment,^403^ which may contribute to neurodegeneration in the basal ganglia of PD. Additionally, manganese could enhance the effects of 6-OHDA on histamine in the brain.^404^ However, in an MPTP-treated PD monkey model, the level of manganese in the brain was not affected by MPTP.^405^ In the DJ-1 knockout PD model mice with MnCl_2_ saline treatment, manganese mainly deposits in subcortical regions, such as ventricles, hippocampus, medial preoptic nucleus (MPO), lateral septal nucleus (LS), and ventromedial hypothalamic nucleus (VMH) as observed by manganese-enhanced MRI and LA-ICP-MS.^406^ Meanwhile, manganese treatment disrupted the homeostasis of iron, zinc, copper, and calcium in the DJ-1 knockout PD model mice, resulting in more severe symptoms of PD. In developing rats, exposure to manganese also induced motor deficits and striatal oxidative stress.^407^ Chronic exposure to MnCl_2_ also establishes a PD zebrafish model involving oxidative stress, neuroinflammation, and apoptosis pathway.^408^ Although no significant changes were observed in markers of dopamine terminal integrity or dopamine receptors in the striatum, manganese exposure caused a decrease in in vivo dopamine release, which may account for manganese-induced motor deficits.^40^ Increased dopamine transporter (DAT) levels were observed in the living non-human primate brain after acute manganese administration using positron emission tomography (PET), which may indicate a compensatory response to its inhibitory action on DAT.^409^ Additionally, manganese can dysregulate the activity of dopamine neurons^410^ and indirectly stimulate the expression of α-synuclein in PC12 cells through activating ERK1/2 MAPK; on the other hand, expression of α-synuclein could enhance neurotoxicity induced by manganese through NF-κB pathway.^411–413^

##### AD

Although some evidence indicates that abnormal manganese levels can play a role in the development of AD, this connection is relatively limited. Despite its capability to bind to the N-terminal part of the Aβ (1–40) peptide, however, manganese exhibits weak binding affinity within the millimolar to micromolar range.^414^ After the subcutaneous injection of MnCl_2_, manganese bound to plaques and enhanced the MRI signal in the 5xFAD mice, indicating a potential role of manganese in imaging amyloid plaques in AD.^415^ A meta-analysis revealed a significant reduction in serum manganese levels in AD patients, which may be associated with cognitive impairment.^416^ The level of plasma Aβ peptides was found to increase with elevated manganese, and high manganese was correlated with Aβ-related cognitive impairment.^417^ The concentrations of manganese were found to be higher in the plasma and lower in the CSF in AD patients compared to controls.^418^ An autopsy of AD patients showed decreased mRNA and protein levels of SOD2 in the hippocampus, which were restored by PKCε activation.^419^ In Cynomologous macaques after intravenous injection of manganese (3.3–5.0 mg/kg/w) for 10 months, increased manganese was found in frontal cortex, accompanied by increased iron, copper and zinc.^420^ Meanwhile, increased Aβ plaques were observed in the frontal cortex, along with degenerative cortical neurons, which may contribute to the cognitive deficits induced by manganese exposure.^420^ Drinking water containing manganese chloride (200 mg/L) for 5 weeks resulted in increased production of Aβ1-40 and Tau in the rat brain, companied by hippocampal degeneration, necrosis, as well as inflammation in peripheral blood and CNS.^421^ Transplantation of gut microbiota from normal rats could not only reduces the levels of Aβ and tau, but also attenuates the neuroinflammation by inhibiting cerebral NLRP3 inflammasomes induced by manganese exposure.^421^ Acute manganese exposure increased the levels of manganese in the cortex, hippocampus, and live in both wild-type control mice and APP/PS1 mice, and impaired glutamatergic function by increasing the level of cortical GLAST protein.^422^ In addition to inducing neuroinflammation in microglia in the hippocampal region, manganese exposure also led to impairment of learning and memory ability, which implicates its neurotoxicity in AD.^193^

##### ALS

A case report has indicated that exposure to manganese after traditional medicine procedures in Kenya promotes the development of ALS.^423^ SOD2, also known as MnSOD, is a homotetrameric enzyme that protects mitochondria against oxidative stress. Immunohistochemical results showed a higher ratio of SOD2-positive neurons to total neurons in the oculomotor nucleus and Onuf’s nucleus compared to normal controls, while a lower ratio was observed in the hypoglossal nucleus of sporadic ALS patients, indicating that sufficient expression of SOD2 could protect neurons from toxic superoxide radicals in the sporadic ALS.^424^ Increased immunoreactivities of both SOD1 and SOD2 were observed in the brain stems of ALS patients, particularly in the terminal phase, in motor neurons and glia.^425^ In a study with ten sporadic ALS patients, increased SOD2 was found in three patients, which also exhibited lower levels of superoxide and decreased Bcl-2.^426^ In the ALS transgenic SOD1^G93A^ mice, partial deficiency of SOD2 significantly reduces survival and exacerbates motor deficits.^427^ In a cell model of ALS, neuronal cell death caused by human SOD1^G37R^ could be attenuated by overexpression of SOD2.^428^ Elevated levels of nitrated SOD2 were also observed in the CSF of ALS patients, which may serve as a biomarker for peroxynitrite-mediated oxidative stress.^429^

##### HD

In comparison to wild-type mice, the HD model mice of YAC128Q exhibited a decreased level of manganese in the striatum, which is the brain region most affected by HD and is highly vulnerable, after exposure to manganese.^430^ XK is responsible for the import of manganese, which is trafficked together with Rab11. It has been reported that impaired XK recycling by Rab11 onto cell surfaces contributes to the decreased vulnerability of the striatum to manganese-induced damage in HD.^431^ The decreased accumulation of manganese was found in both HD human neuroprogenitors and HD mouse striatal cells, which was also associated with an altered manganese-dependent ATM-p53 pathway.^432^ The resistance to manganese sensitivity in HD is different between lineages and developmental stages. It was observed that the HD genotype increased the sensitivity of early post-mitotic midbrain neurons to manganese, but it had no significant influence to post-mitotic cortical neurons and HD human-induced pluripotent stem cells-derived neuroprogenitor cells.^433^ After exposure to manganese, premanifest YAC128 mice exhibited suppressed transcriptional and protein changes, while manifest YAC128 mice showed a diminished response to metabolic changes.^434^ In wild-type mice exposed to subtle manganese, behavioral changes were induced and neuron density in the striatum was reduced; however, YAC128 mice were able to protect against these aforementioned changes.^435^ Manganese exposure also induces hyperactivity and dopaminergic dysfunction, with the effects being dependent on sex, age, and YAC128 genotype.^436^ Manganese also functions as an insulin/IGF receptor to phosphorylate Akt and participate in glucose uptake, and decreased manganese levels may contribute to impaired IGF signaling and glucose uptake in HD.^437^ Acute manganese treatment could restore the reduced autophagic cargo loading in HD cells, indicating that manganese deficiency may contribute to the impaired autophagy flux in HD.^438^ At the early stage of HD, mutant HTT could cause a deficiency of neuronal manganese, which affects arginase activity and contributes to the pathophysiology of urea cycle dysfunction in the striatum of HD.^439^

#### Dysregulation of copper

##### PD

Occupational exposure to copper also increases the risk of PD. It has been found that combining chronic copper exposure with aging induces PD features, including altered motor function, dopaminergic neuronal loss, increased α-synuclein accumulation, and aggregation, as well as alterations in proteasome and autophagy.^440^ Recently, dietary intake of copper has also been reported to be associated with the risk of PD.^441^ In PD patients, an increase in concentration of copper in CSF has been observed, and this copper concentration has been found to be correlated with severity and progression of PD.^442,443^ Although a significantly increased level of copper was observed in the GP, putamen, and amygdala of rats over time after 6-OHDA treatment,^403^ a significant decrease in copper was observed in the SN and locus coeruleus of PD patients using synchrotron-based x-ray fluorescence microscopy (SRXFM), as well as in their blood concentration of copper and Cp.^443–445^ Widespread copper decreases in copper levels were observed in the primary motor cortex (MCX), cingulate gyrus (CG), primary visual cortex (PVC), hippocampus, SN, medulla oblongata (MED), and middle temporal gyrus (MTG) of individuals with PD dementia, as determined by ICP-MS.^446^ All these pieces of evidences indicate a potential relationship between decreased copper levels and PD.^447,448^ The bridge linking copper deficiency with PD may be the aberrant iron, as copper predominately binds with Cp, triggering its ferroxidase activity to convert toxic ferrous iron into nontoxic ferric form and maintain iron homeostasis; however, insufficient copper can lead to iron deposition and neuronal death.^449^ Copper can directly bind to α-synuclein and promote the aggregation of α-synuclein.^450–452^ Additionally, both copper and iron have been suggested to accelerate the prion-like propagation of α-synuclein fibrils in PD because α-synuclein fibrils formed in the presence of copper or iron were more cytotoxic.^453^ The folded conformation is stabilized by the combination of copper, which alleviates the electrostatic repulsion among the negative charges.^454^ However, acetylation at the N-terminus or pathological H50Q mutation of α-synuclein can disturb its binding with copper and inhibit copper-induced aggregation.^455^ After aggregation, copper also facilitates the release of mature fibrils of α-synuclein into the extracellular environment.^453^ The combination of copper leads to an increased neurotoxicity caused by α-synuclein.^456^ While the overexpression of α-synuclein for a short time had no toxic effect on dopaminergic neurons, cell death occurs after exposure to copper due to increased CTR1 and depleted GSH, and the oxidative stress induced by copper is mediated through modulation of autophagy or the ubiquitin-proteasome system.^457^ In transgenic human α-synuclein (A53T) mice and SH-SY5Y cells overexpressing of human α-synuclein (A53T), low-dose copper exposure was found to enhance the accumulation of α-synuclein, which was associated with mitochondrial impairments, including excessive ROS production and reduced mitochondrial ATP production.^458^ In addition, defects in copper transportation may be involved in PD, as evidenced by a reduction of CTR1 in neurons of low-copper concentration encephalic regions, which is crucial for copper uptake.^445,458,459^ Furthermore, the aggregation of α-synuclein caused by copper stimulation can be inhibited by silencing CTR1, the primary pathway for copper uptake.^460^ This finding is consistent with the results showing a reduction in α-synuclein aggregates after knockdown of CTR1 in yeast and mammalian cell models expressing human α-synuclein.^461^ Furthermore, deficiency of CTR1 could significantly reduce the phosphorylation of α-synuclein at S129 and alleviate nigrostriatal degeneration in an AAV-based mouse model of PD.^461^

##### AD

Replication studies and meta-analysis have shown that serum copper levels are higher in AD patients that in healthy controls.^462^ In the APP^NL-G-F^ knock-in mice, both age and AD development were found to affect copper level in the blood plasma; however, only AD was found to have an impact on the blood plasma copper isotope ratio, and a significant increase in copper was also observed in the brain stem of both young and aged AD mice compared with healthy controls.^463^ Elevated labile copper was also observed in the postmortem cortical tissue from AD patients, which was associated with oxidative pathology of AD.^464^ APP, which can produce neurotoxic Aβ through sequential protease cleavage and contribute to the pathology of AD, has a high affinity with copper at its N-terminal and facilitates its reduction from Cu^2+^ to Cu^1+^, which process could promote the production of hydroxyl radicals and induce AD-related toxicity.^465^ On the other hand, this binding between APP and copper also affects copper levels, as evidenced by studies on transgenic mouse brains. Overexpression of AAP containing Aβ significantly reduces the levels of copper and iron, while overexpression of APP in Tg2567 transgenic mice only reduces the level of copper.^466^ Increased copper was observed in the cerebral cortex and liver of APP and APLP2 knockout mice.^467^ Additionally, copper can enhance the localization of APP on the cell surface by increasing exocytosis and reducing endocytosis.^468^ Transthyretin can directly bind to Aβ and modulate its accumulation, which was lower in the CSF of AD patients, and it has been reported that copper regulates the binding between Aβ and transthyretin.^469,470^ Copper can enhance the proportion of beta-sheet and alpha-helix compositions within the Aβ proteins, thereby facilitating the formation and accumulation of Aβ in the brain plaques.^471^ Additionally, the Cu-Aβ complex not only activates microglia and reduces the release of TNF-α and nitric oxide through NF-κB mediation,^472^ but also downregulates the expression of LRP1, impeding the clearance of neurotoxic Aβ and triggering the buildup of brain deposits.^184^ Recently, different impacts of copper on the assembly of Aβ40 and Aβ42 isoforms have been reported.^473^ Copper increases primary nucleation and promotes the fibril formation of Aβ40, resulting in enhanced generation of neurotoxic oligomers; on the other hand, copper causes the disassembly of Aβ42 fibrils and induces protofibrils and oligomers.^473,474^ Oxidative stress can influence the process of Aβ assembling into β-sheet rich fibrils in AD by causing oxidation of Aβ. In the presence of copper, oxidized Aβ was found to assemble into weakly-structured and untangled Cu-Aβ fibrils.^475^ Copper can also bind with tau peptide and modulate the aggregation of tau.^476,477^ In addition, excess copper was found to enhance the phosphorylation of tau proteins in human neuroblastoma cells, and lowering the level of copper by oral zinc could significantly decrease tau phosphorylation in the transgenic mice model expressing wild-type human tau protein^478^ and attenuate spatial memory impairment in female PS19 mice.^479^ Although there is ample evidence of copper’s involvement in Aβ accumulation and tau phosphorylation, compelling evidence also indicates that increasing copper levels in the brain has the potential to mitigate amyloid pathology and offer positive effects for AD. For example, increasing the bioavailability of copper with compound Cu(II)(gtsm), which can remain at high level in the brain after uptake, could inhibit Aβ oligomers and tau phosphorylation while restoring cognitive function in APP/PS1 transgenic AD model mice.^480,481^

##### ALS

Increased copper in the ALS cells co-localized with total lipids, leading to an increase in oxidized lipids and potentially directly inducing oxidative stress.^482,483^ The increased influx of copper, upregulation of copper chaperones, and decreased efflux of copper resulted in the accumulation of copper in spinal motor neurons, which was associated with age-dependent increases in copper in the spinal cord.^484^ As a copper-regulating protein, metallothioneins can bind copper ions and decrease oxidative toxicity. SOD1, also known as copper/zinc SOD, can protect mitochondria from oxidative damage.^485^ Mutations in SOD1, including D90A, A4V, G93A, and G37R, have been associated with familial ALS.^486^ In the SOD1^G93A^ mice, increased levels of metallothioneins-I and metallothioneins-II were observed within astrocytes in both white and gray matter as mice age, and increased levels of metallothioneins-III were observed in neurons, while elevated metallothioneins-III levels were observed in glial cells at the later stages of disease.^487^ In the SOD1 Tg mice, increased levels of copper and lipid peroxides were found in the spinal cord at the age of 8 and 16 weeks, while zinc levels were decreased.^488^ However, motor paralysis and increased metallothionein-III were only observed at 16 weeks of age, indicating that metallothioneins-I and metallothioneins-II function at the early stage of ALS, whereas metallothioneins-III function during the late stage. The reduction of metallothioneins promotes disease expression,^489^ while the overexpression of metallothionein-I can significantly improve copper dyshomeostasis and extend the lifespan of SOD1^G93A^ mice.^490^ Mutation in the copper transporter ATP7A^M1311V^, which was found in ALS patients, also caused intracellular copper accumulation and impaired motor neurons.^491^ Increased hydroxyl radical-generating activity was found in the G93A mutant SOD1, which facilitates copper release and induces oxidative stress through the Fenton reaction.^48,492^ Furthermore, mutant SOD1 loses its specificity in metal ion binding, which may play a role in ALS.^493^ Mutant SOD1 aggregates have the ability to bind and aggregate with anti-apoptotic protein Bcl-2 in spinal cord mitochondria,^494^ and misfolded mutant SOD1 has been found to deposit onto the cytoplasmic face of the outer mitochondrial membrane.^495^ Mutant SOD1 could activate caspase-1 and caspase-3, which were observed in the ALS transgenic mice and cell model, triggering apoptosis,^496,497^ suggesting that inhibition of caspase is protective in the ALS.^498^ In the primary cultured astrocytes overexpressing wild-type SOD1 or mutant SOD1^G93A^, both wild-type and G93A mutant SOD1 can be secreted by astrocyte through exosome in varying amount.^499^ Moreover, the secreted SOD1^G93A^ through exosome is transferred from astrocyte to spinal neurons, resulting in selective motor neuron death. Increasing the total and mitochondrial NAD (+) content in astrocytes expressing mutant SOD1 can protect co-cultured motor neurons from death.^500^ Motor neurons can secrete active endogenous SOD1; however, this secretion is impaired by mutant SOD1, which causes cytoplasmic inclusions and neuronal toxicity.^501^ The interaction between mutant SOD1 and dynein-dynactin complex has been found to contribute to inclusion formation.^502,503^ Additionally, oxidative modification of Tryptophan 32, occurring in both wild-type and mutant SOD1, can promote the aggregation and toxicity of SOD1^G93A^.^504^ As the disease progression and endpoint were significantly delayed following chronic intraspinal injection of exogenous wild-type hSOD1 in the SOD1^G93A^ rat, it was suggested that extracellular SOD1 has neuroprotective effects in ALS.^501^ Mutant TAR DNA binding protein-43 (TDP-43) is also associated with the development of frontotemporal lobar degeneration and ALS. Increased levels of copper, zinc, and manganese in the spinal cords, but not in the brain, may explain the decline in locomotion observed in TDP-43 (A315T) mice.^505^

##### HD

Copper has the ability to bind with the N-terminus of HTT proteins and further stimulate their aggregation.^506^ In a *Drosophila* model of HD with HTT exon 1-polyQ, knockdown of *Ctr1B* and *DmATP7* genes, which are involved in copper metabolism, can affect the progression of HD, and the level of HTT aggregation significantly decreases after reducing copper.^507^ Meanwhile, the copper-enhanced toxicity of HTT dissipated after substituting Met8 and His82, which are potential copper-binding residues in HTT.^507^ In a Drosophila model of HD, copper dose-dependently increased the aggregation of mutant HTT, promoted the accumulation of Thioflavin S positive β-amyloid structures in HTT aggregates, and altered the autophagy-lysosome pathway.^508^ Furthermore, the progression of HD may be exacerbated by copper’s inhibitory effect on mitochondrial dehydrogenases, particularly succinate dehydrogenase (SDH) and lactate dehydrogenase (LDH), which are sensitive to copper and become inactivated. LDH plays a pivotal role in the astrocyte-neuron lactate shuttle mechanism, utilizing lactate and providing energy for neurons.^509,510^ The blockage of lactate metabolism in the brain can lead to an insufficient energy supply, which contributes to HD.^506,510^ Although elevated copper levels have been found in HD patients and models, in a QUIN-induced HD rat model, supplement of copper in drinking water (90 ppm Cu, 28 days) reduced lipid peroxidation and ROS, possibly due to increased SOD1 activity.^511^

#### Dysregulation of zinc

##### PD

The mainstream view suggests a reduction in zinc concentration among PD patients, and a comprehensive meta-analysis has demonstrated a significant downward trend in zinc levels in plasma, serum, and CSF.^512–515^ Serum zinc deficiency has been proposed as a risk factor for the development of PD dementia.^516^ Zinc deficiency also exacerbated movement disorders and dopaminergic neurodegeneration in MPTP-induced PD mice.^517^ In a *Drosophila* model of PD, providing sufficient zinc significantly enhances both lifespan and motor functions.^518^ As we know, oxidative stress plays a significant role in the development of PD, including lipid peroxidation and nucleic acid oxidation. It’s worth noting that zinc possesses antioxidative properties. Therefore, the decrease in zinc in PD patients may contribute to excessive antioxidative reactions. In a rotenone-induced PD rat model, adequate zinc supplementation alleviated neuronal damage by inhibiting lipid peroxidation, suggesting a protective role of zinc against oxidative stress.^519^ However, zinc also plays a toxic role in promoting the degeneration of dopaminergic neurons within the SN.^520^ In the 6-OHDA-induced PD rat model, a significantly increased level of zinc was observed in the GP, putamen, and amygdala over time after 6-OHDA treatment.^403^ Additionally, increased zinc levels were observed in the SNpc of paraquat-treated rats.^521^ Mutations within the ATP13A2 gene (PARK9) are the underlying cause of Kufor-Rakeb syndrome (KRS), which leads to juvenile-onset Parkinsonism. PARK9, encoded by the *ATP13A2* gene, is abundantly expressed in nigral dopaminergic neurons, and mutations within *ATP13A2* can cause hereditary Parkinsonism with dementia.^522^ Deficiency in *ATP13A2* results in dysregulation of lysosomes and aggregation of α-synuclein,^523^ possibly due to zinc dyshomeostasis.^524,525^

##### AD

Due to its ability to combine with amyloid plaques and accelerate the aggregation of Aβ peptides and tau protein, zinc is also implicated in the progress of AD. In clinical detection of AD patients, elevated levels of zinc have been observed in the cortex, hippocampus, and amygdala, which are all highly vulnerable brain regions in AD.^526,527^ In AD animal models such as *APP*/*PS1* mice and macaques, zinc was found to accumulate in the plaques.^528,529^ Certain zinc-bearing transcription factors, including NF-κB and the tumor suppressor protein p53, are involved in the synthesis of APP. Zinc can interact with Aβ nonspecifically, and the Aβ–zinc complex exhibits high resilience against proteolysis, thereby enhancing the persistence of Aβ aggregations.^530,531^ In addition to copper, oxidized Aβ was also found to assemble into long untangled zinc-Aβ fibrils in the presence of zinc.^475^ Furthermore, Aβ aggregation can stimulate the release of zinc from metallothionein, potentially disrupting mitochondrial function and stimulating the generation of ROS, which in turn promotes zinc release and triggers apoptosis.^532,533^ In relation to the tau protein, accumulating evidence indicates that elevated zinc may potentially contribute to the excessive phosphorylation of the tau protein by activating extracellular signal-regulated kinase such as GSK-3β and MAPKs, while inactivating the main serine/threonine phosphatase in brain tissues, protein-phosphatase 2A (PP-2A), thereby disrupting the homeostasis of tau protein.^534–537^ Excessive phosphorylation is more likely to accumulate in neurons, leading to the formation of non-degradable neurofibrillary tangles that resist proteolytic degradation. This accumulation subsequently disrupts axonal transport and microtubule synthesis, resulting in neurotoxicity.^538,539^ However, some literature reports a significant decrease in zinc level in AD patients and suggests that zinc deficiency is a potential risk factor of AD.^540,541^ The depletion of intracellular zinc destabilizes microtubules, triggering a chain reaction that includes the release of tau protein, hyperphosphorylation, and the formation of neurofibrillary tangles. Overall, a plethora of experiments support the improvement of cognitive performance after zinc supplementation.^542^ Since 1992, zinc has been permitted as a potential therapeutic approach for AD, administered both orally and parenterally. On the one hand, zinc supplementation restores the serum zinc level; however, it simultaneously decreases the level of serum-free copper by impeding its absorption in the intestine. This leads to an upregulation of metallothionein for binding copper. Nevertheless, the supplementation of zinc has side effects that exacerbate tauopathy in both behavioral and biochemical impairments, suggesting that zinc should be avoided unless absolutely necessary.^543^ Variations in intracellular zinc levels potentially arise from modifications of ZIP or ZnT proteins, which are respectively responsible for the influx of zinc from the extracellular environment and the redistribution of zinc to either the extracellular milieu or intracellular components. In neurons, the ZnT3 transporter is responsible for pumping free zinc into synaptic vesicles. Knocking down ZnT3 in mice leads to cognitive difficulties occurring earlier than expected.^149^ An interesting discovery revealed a decline in ZnT3 levels in the brains of mice and humans with aging; however, an even more significant reduction was observed in AD patients.^544^ Despite the downregulation of ZnT3, compensatory upregulations of ZnT4, ZnT6, and ZnT1 were observed in the hippocampus of AD.^545,546^

##### ALS

A high level of labile zinc was observed in the neurons and astrocytes in the spinal cords of SOD1^G93A^ mice, which may be due to increased HNE levels.^547^ The native SOD1 protein is expressed as a homodimer in the cytosol, capable of binding with either four copper or four zinc ions.^548^ However, mutations in SOD1 impair dimerization and promotes its aggregation in ALS.^549^ Zinc can stabilize the native structure of the SOD1 monomer and promote its homo-dimerization. Copper chaperone (Ccs) binds to SOD1 and facilitates its binding with zinc, thereby stabilizing the conformation of SOD1; however, this process is disrupted by mutant SOD1.^550^ The absence of zinc but not copper, significantly impacted the membrane attachment of SOD1 and facilitated its aggregation in an in vitro ALS model.^551^ However, initiation and seed growth in the fibrillation SOD1 were controlled by the disulfide bond rather than zinc or dimerization.^552^ The loss of zinc in the SOD1 could induce motor neuronal death, which plays a causal role in ALS.^553^ Under zinc-deficient conditions, SOD1 functions as a molecular switch to initiate homeostatic ER stress.^554^ Aberrant zinc binding to SOD1 also triggers amorphous aggregation.^555^ Decreased levels of zinc transporters ZnT3 and ZnT6 were observed in the spinal cords of sporadic ALS patients.^556^ Zinc can bind to RRM2 peptide of TDP-43.^557^ Additionally, zinc can bind to the RNA recognition motif of TDP-43, which induces the formation of amyloid-like aggregates.^558^ Zinc was found to specifically induce the depletion and aggregation of endogenous TDP-43, which was not observed in copper, iron or H_2_O_2_.^559^ Although the induction of apoptosis in motor neuronal death by human mutant SOD1^G93A^ depends on copper and the absence of zinc, chronic oral administration of zinc sulfate decreased the survival of SOD1^G93A^ mice, indicating that zinc amplifies the SOD1^G93A^-mediated toxicity.^560^ Zinc pre-treatment also enhanced the NMDAR-mediated excitotoxicity in cultured cortical neurons derived from SOD1^G93A^ mice.^561^ Additionally, zinc migration and subunit swapping in SOD1 may contribute to its neurotoxicity in ALS.^562^ Cytoplasmic accumulation and aggregation of SFPQ are hallmarks of ALS. Zinc was found to bind with SFPQ, a ubiquitous nuclear RNA-binding protein, and induce infinite polymerization of SFPQ in primary cortical neurons.^563^ Aggregation of FUS protein is another hallmark of ALS.^564^ Zinc could enhance liquid-liquid phase separation of FUS protein and promote its aggregation.^565^

##### HD

In R6/1 HD mice model, a significant reduced level of zinc was observed in the hippocampus and cortex, where typically have a high concentration of zinc.^566^ This deficiency exacerbated the deficit in hippocampal long-term potentiation (LTP) and diminished AMPA receptors. In the N171-82Q HD transgenic mice, decreased levels of synaptic vesicular zinc and declined transcriptional activity of ZnT3 were observed in the hippocampus, cortex, and striatum.^567^ These changes were caused by mutant HTT inhibiting Sp1 from binding to the promoter of the ZnT3 gene. In a *C. elegans*-based HD model, chronic exposure to copper, zinc, or their mixture can cause neurodegeneration by increasing the aggregation of polyQ protein in the muscles and neurons.^568^ Neuronal zinc finger protein (ZFP) transcriptional repressors can significantly reduce mutant HTT and rescue HD-associated behavioral and molecular phenotypes.^569^

### Crosstalk between metal ions dysregulation and pathological proteins

Lewy bodies are composed of a large amount of misfolded α-synuclein, which co-localizes with redox-active iron (Fig. 5). The toxic interaction between iron deposition and α-synuclein aggregation has been demonstrated to accelerate the progression of PD.^10,11^ We have also reported that the toxic combination of α-synuclein and iron induces cell senescence in a PD mice model, which occurs prior to the loss of nigral dopaminergic neurons.^23^ Reports from our lab and other labs have demonstrated that iron can induce α-synuclein aggregation. In addition to APP, IRP can also bind to the IRE-mRNA of α-synuclein and downregulate its protein level and aggregation.^340^ However, in the presence of iron overload, the protein levels of α-synuclein and its aggregation can be upregulated by IRP, establishing a link between IRP-mediated iron homeostasis and PD. Conversely, α-synuclein also affects iron in the process of PD. Nasal mucosal delivery of human PFFs caused time-dependent iron deposition in the SN and GP in male *Macaca fascicularis*.^208^ Aggregated α-synuclein could inhibit the iron release from ferritin by inhibiting v-SNAREs in PD.^315^ Manganese can indirectly stimulate the expression of α-synuclein, which contributes to the aggregation of α-synuclein in glial cells and neurons, and α-synuclein can also enhance the manganese-induced neurotoxicity.^411–413^ As a copper-binding protein, copper can directly bind to α-synuclein and promote its aggregation.^450–452^ Additionally, both copper and iron have been suggested to accelerate the prion-like propagation of α-synuclein fibrils in PD, because α-synuclein fibrils formed in the presence of copper or iron exhibit were more cytotoxic.^453^ Overexpressed α-synuclein was also found to exacerbate copper toxicity through the autophagy-lysosome pathway or ubiquitin-proteasome system.^457^ Furthermore, zinc deficiency is also implicated in the aggregation of α-synuclein.^523^

Dysregulation of cerebral transition metal ions, such as iron, copper, and zinc, has been implicated as a precursor event for Aβ aggregation in AD (Fig. 5). Ferric iron can interact with Aβ and promote the aggregation of both Aβ40 and Aβ42.^346,347^ Additionally, Aβ42 can induce the accumulation of ferric iron within amyloid aggregates and lead to the reduction of ferric iron into ferrous iron.^350^ Redox-active iron was co-localized with both senile plaques and neurofibrillary tangles in the hippocampal tissue of AD.^29^With the LA-ICP-MS, significantly higher concentrations of iron, copper and zinc have been observed in the hippocampus, cortex and retina of 9-month-old wild-type mice compared to the APP/PS1 mice.^570^ Analysis of the isotope ratios of iron, copper, and zinc in transgenic tau mice and 5×FAD mice showed a significant pathology-specific distribution of both iron and zinc in the mouse models of AD.^571^ Tau can traffic APP to facilitate iron efflux. Lowered levels of brain tau caused by lithium has been found to elevate nigral and cortical iron levels through attenuating iron efflux, which may be associated with the adverse effect of lithium such as PD-like hand tremor and limit its application for the treatments of neurodegenerative or neuropsychiatric disorders.^572^ Overexpression of the carboxyl-terminal fragment of APP, which contains Aβ, could significantly reduce the levels of both iron and copper in the transgenic mouse brain; however, overexpression of APP only reduced the level of copper in Tg2576 transgenic mice.^466^ Concomitant increased levels of manganese were observed in the brains of both transgenic mice.^466^ Although excessive copper and zinc may induce neocortical Aβ production in AD, both copper and zinc at physiological concentrations could promote the degradation of soluble Aβ.^573^ However, the mechanisms by which they reduce the secreted level of Aβ differ between copper and zinc.^574^ Zinc induces dimerization of APP-C99 and prevents its cleavage by γ-secretase, while copper directly targets the subunits presenilin and nicastrin in the γ-secretase complex, reducing the production of Aβ.^574^ In addition, oral zinc could lower the level of copper, significantly reducing tau phosphorylation in a transgenic mouse model expressing wild-type human tau protein^478^ and alleviating spatial memory impairment in female PS19 mice.^479^ By using synchrotron Fourier transform infrared micro-spectroscopy (FTIRM) to image the in situ secondary structure of amyloid plaques and synchrotron X-ray fluorescence (SXRF) microprobe to detect the accumulation of metal ions in the same brain tissue of AD patients, accumulations of both copper and zinc were first observed in Aβ deposits.^43^ Recently, after comparing the images of Aβ detection by synchrotron X-ray phase-contrast tomography (XPCT) among four transgenic AD animal models, including APPPS1 mice, ArcAβ mice, J20 mice (zinc and iron accumulation), and TgF344 rat (copper accumulation), which had similar β-sheet content but varying metal levels, hyper-density of Aβ plaques was observed in both J20 mice and TgF344 rats.^575^ In contrast, hypo-density with a hyperdense core was observed in APPPS1 and ArcAβ mice. These findings suggest that metal accumulation may be the primary factor enabling XPCT to map the distribution of Aβ plaques throughout the entire brain without labeling.

Cell membranes also play a crucial role in the metal ion-mediated amyloid aggregation in the brain environment.^576^ The effect of metal ions, such as copper and zinc, on Aβ toxicity, complicates the interaction between Aβ peptide and cell membrane.^577^ Studies from the lab of Pappalardo have investigated the Aβ amyloidosis and fibrillogenic properties of Tau/Aβ in the presence of copper and zinc.^578,579^ Using large unilamellar vesicles (LUVs) to mimic the lipid composition of neuronal membranes, the conformational changes of Tau_26-33_, but not Tau_9-16_, were found to be enhanced by copper and zinc in the presence of membrane surfaces. Notably, the aggregation rate of Tau_26-33_ was decreased by LUVs/copper and LUVs/zinc; however, the aggregation rate of Tau_9-16_ was increased by LUVs/copper and LUVs/zinc. Additionally, both copper and zinc were found to enhance the interaction between Aβ_40_ and membrane surfaces and cause the formation of amyloid aggregates. All these pieces of evidence indicate the interplay between metal ions and Tau/ Aβ on the cell membrane, which may play an important role in AD by affecting amyloid formation. Amyloid fibril aggregates, such as senile plaques, also provide large surface areas for metal ions. When combined with metal ions, these bio-interfaces not only induce misfolding of peptides but also modulate both the primary and secondary nucleation of peptide monomers from the microscopic step.^580^ The surface-chelated copper has been reported to dynamically interact with Aβ chains, restricting their two-dimensional diffusivity on the surface and slowing down their fibrillation.^581^ In contrast, surfaces without copper facilitate the two-dimensional diffusivity of Aβ chains, promoting improved interpeptide interaction and accelerating Aβ fibrillation. This highlights the critical role of surface-chelated copper in both Aβ fibrillation and AD progression.

Aggregation of mutant SOD1 is a common hallmark of ALS. In transgenic SOD1^G93A^ mice, the overexpression of Aβ promotes the onset of motor impairment and aggregation of SOD1^G93A^, accompanied by elevated levels of copper and zinc in the brain and spinal cord.^582^ Upregulated APP in muscle fibers coincides with symptom onset in both sporadic ALS patients and SOD1^G93A^ mice, while genetic ablation of APP significantly improves multiple disease parameters in the SOD1^G93A^ mice.^583^ Although high levels of SOD1 are also found in the nigrostriatal dopaminergic neurons, SOD1 mutations have a greater cytotoxic effect on motor and dopaminergic neurons.^584^ The absence of zinc, but not copper, significantly affects the membrane attachment of SOD1 and promotes its aggregation in an in vitro ALS model.^551^ Aberrant binding of zinc to SOD1 also triggers amorphous aggregation.^555^

HTT has been reported as an iron-regulated protein,^398^ and the elimination of HTT expression alters brain iron homeostasis in adult mice,^399^ indicating that HTT might play an important role in the dysregulation of iron in HD. In the early stages of HD, mutant HTT could cause a deficiency of neuronal manganese, which affects arginase activity and contributes to the pathophysiology of urea cycle in HD striatum.^439^ Copper has the ability to bind with the N-terminus of HTT proteins and further stimulate their aggregation.^506^ In a *Drosophila* model of HD, copper increases the aggregation of mutant HTT in a dose-dependent manner.^508^ In N171-82Q HD transgenic mice, mutant HTT was found to inhibit the binding of Sp1 to the promoter of ZnT3 gene, resulting in decreased levels of synaptic vesicular zinc in the hippocampus, cortex and striatum.^567^

## Treatment targeting mental ions in neurodegenerative diseases

Metal chelators, capable of sequestering metal ions and dissociating them from their target sites through the formation of multiple coordinate bonds, have emerged as promising therapeutic targets for modulating redox processes and mitigating abnormal protein aggregation. In this part, we summarize the treatment targeting mental ions for neurodegenerative diseases.

### PD

#### Metal ions chelation

##### DFO and CQ derivative

DFO, an outstanding molecule widely used to treat diseases caused by excess iron, has been approved by the Food and Drug Administration for many years.^585^ DFO has been shown to be strongly neuroprotective in several PD animal models, improving motor defects and enhancing the survival of dopaminergic neurons following treatment with MPTP,^586^ rAAV-α-synuclein,^587^ 6-OHDA,^588–590^ and rotenone^591^ (Table 2). In addition to chelating iron, increasing the expression levels of GPX4 and ferritin heavy chain, DFO has been shown to directly reduce the aggregation of α-synuclein.^592^ Furthermore, it can activate insulin signaling and glucose metabolism,^593^ accumulates HIF-1α to induce autophagy,^594^ exerting its roles through multiple targets. Intranasal delivery, a non-invasive method of bypassing the BBB to deliver therapeutics along the olfactory and trigeminal nerve pathways to the brain, improves the application and efficiency of DFO.^593^ Furthermore, a polymeric nanoparticle system capable of intracerebrally delivering DFO could also overcome the disadvantages of its short half-life in vivo and poor penetration through the BBB. You et al. report a nanoparticle system loaded RVG29 peptide through specific receptor-mediated endocytosis, resulting in significant decreases in iron content and oxidative stress levels in the SN of PD mice without any adverse effects.^595^ This DFO-based nanoformulation proposes another potential strategy for delivering DFO into the brain. CQ has been extensively utilized as both an antifungal and antiprotozoal agent, exhibiting emerging efficacy in the treatment of neurodegenerative disorders. One of its mechanisms involves its ability to interact with metals.^596^ It has been reported that oral administration of CQ provides protection against MPTP-induced damage in mice by inhibiting iron overload.^14,597^ Furthermore, CQ significantly improves motor and non-motor deficits in a stabilized monkey model of PD induced by MPTP, primarily targeting iron content and ROS levels in the SN.^598^ The Akt/mTOR pathway plays a crucial role in this process. Additionally, CQ attenuates zinc-mediated cytotoxicity in mice injected with LB-extracts derived from PD patients containing toxic α-synuclein aggregates, resulting in reduced lysosomal alterations and dopaminergic neurodegeneration.^599^ A novel quinazolinone compound, named PBT434, is being developed for the treatment of PD. In vitro studies have demonstrated that PBT434 can inhibit iron-mediated redox activity and α-synuclein aggregation.^600^ In vivo, PBT434 rescued motor performance and reduced nigral α-synuclein accumulation in 6-OHDA and MPTP-treated or transgenic mice (hA53T α-synuclein).^600^ Importantly, PBT434 did not deplete tissue iron stores; instead, it increased levels of FPN and DJ-1. Furthermore, PBT434 modulates the uptake of iron by human brain microvascular endothelial cells through chelation of interstitial iron and inhibition of iron re-uptake by endothelial cells of the BBB.^601^ Importantly, not only does PBT434 inhibit hyposmia and accumulation of iron and copper in the bulbar region in young tau-/-mice, but it also attenuates motor impairment and neurodegeneration in the SN in aged tau-/-mice, suggesting its potential as a therapeutic agent for both prodromal and clinical stages of PD.^602^

**Table 2:** Molecules or pathways of metal chelators in treating neurodegenerative diseases

Compounds	Ions	Disease	Model	Molecules/pathways	Ref
DFO	Iron	PD	MPTP, α-synuclein rAAV, 6-OHDA, and rotenone-induced mice	Chelate iron; Increased the expression level of GPX4 and FTH1; Induce autophagy; Activate insulin signaling and glucose metabolism	^586–591^
	Iron	AD	P301L tau transgenic mice; APP/PS1 mice	Suppress ferroptosis; Inhibitor GSK-3β; Targeted HIF-1α to activate GLUT1	^355,643–648^
	Iron	HD	R6/2 HD mice	Improve the motor phenotype	^392^
CQ	Iron Zinc Copper	PD	Zn-treated mice injected with α-synuclein; MPTP -treated monkey model	Target iron content and ROS levels; Activate AKT/mTOR pathway; Reduce lysosomal alterations	^14,597–599^
	Iron Zinc Copper	AD	An APP transgenic mouse tau-knockout mice	Non-specificly chelate copper-zinc; Improve cognitive behavior and decrease Aβ deposits; Resumpt Cu^2+^-suppressed fibril growth of Aβ (1-40)	^652–654^
	Iron Zinc Copper	HD	PC12 cells expressing Htt exon-1 R6/2 mice	Decrease mutant protein expressing polyglutamine-expanded huntingtin exon 1; Improve behavioral and pathologic phenotypes	^754^
PBT434	Iron Copper	PD	6-OHDA and MPTP treated or hA53T α-synuclein mice; Iron-induced M17 neuroblastoma cells	Inhibit iron-mediated redox activity; Inhibit re-uptake of iron by endothelial cells; Inhibit hyposmia and accumulation of iron and copper	^601,602^
DFP	Iron	PD/AD	H_2_O_2_/iron/Aβ1–40-induced primary cortical neuron; MPP^+^-induced SHSY-5Y cells	Iron chelator; Protects against neuronal cell death	^763^
	Iron	AD	Rabbits fed a cholesterol-enriched diet	Reduce Aβ and Tau phosphorylation levels; Reduce plasma iron and cholesterol levels	^764^
	Iron	HD	R6/2 HD mice	Remove mitochondrial iron to decrease lipid peroxidation; Improve motor endurance	^396^
DFP-chromonehybrids	Iron	AD	scopolamine-induced ICR mice	MAO-B inhibitors and iron chelators for the treatment of AD	^765^
DL-3-n-butylphthalide	Iron	PD	Rotenone-induced rats	Improve iron deposition within SN; Inhibit serum iron levels; Inhibit ferroptosis	^603^
Paeoniflorin	Iron	PD	MPP^+^-induced primary dopaminergic neurons	Activate the Akt/Nrf2/Gpx4 pathway; Inhibit ferroptosis	^604^
Quercetin	Iron Copper Manganese	PD	Erastin/RSL3 and MPP^+^-induced cells; MPTP-induced mice, Cu-induced SH-SY5Y cells; Mn-induced SK-N-MC cells and SD rats	Inhibit ferroptosis; Modulate autophagy; Activate iNOS/NF-κB and HO-1/Nrf2 Pathways	^605–607^
Quercetin	Copper	AD	Cu-induced SH-SY5Y cells	Regulate PI3K/Akt and ERK1/2 signaling	^606,676^
Sterubin fisetin	Iron	PD	Glutamate/iodoacetic acid treated HT22 cells; LPS-treated BV-2 cells	Induce Nrf2, neurotrophic factors, inflammation restriction, and iron chelating	^678,766^
		AD	RSL3 induced-HT22 cells	Restore mitochondrial homeostasis	
Epigallocatechin-3-gallate	Iron Copper Zinc	PD	Fe^3+^-induced AS-PC12 cells; MPTP-induced mice; PINK1 mutant Drosophila;	Inhibit ferroptosis; Bind to metal [Cu^2+^ and Zn^2+^]-Aβ monomers and dimers	^608–611^
Emodin	Zinc	PD	Zn-induced SH-SY5Y cells	Inhibit cell apoptosis; Reduce ROS and ER-stress levels	^612^
Thonningianin A	Iron	PD	6-OHDA in zebrafish and dopaminergic neurons	Inhibit ferroptosis; Inhibit Keap1-Nrf2 protein-protein interaction; Activate Atg7-dependent autophagy	^613^
(-)-Clausenamide Clau	Iron	PD	MPP^+^-induced SH-SY5Y cells; N2a and PC12 cells; MPTP-induced mice	Scavenge lipid peroxide products; Target Ser663 on ALOX5; Inhibit ferroptosis	^614^
*E. amoenum* extract	Manganese	PD	Mn^2+^-treated rat	Increased catecholamine content and improved depression-like behavior	^615^
*Euterpe oleracea Mart*.extract	Manganese	PD	Mn-induced rat primary astrocytes	Inhibit oxidative stress	^616^
*Euphorbia supina* extract	Manganese	PD	Mn-induced SKNMC cells and SD rats	Regulate ER stress and it mediated apoptosis	^617^
*Melissa officinalis aqueous* extract	Manganese	PD	Mn-induced mice	Inhibit oxidative stress	^618^
*Boldo Aqueous* extract	Manganese	PD	Mn-induced D. melanogaster	Improve movement disorders	^628^
M30	Iron	PD	Adult mice; MPTP-induced mice	Enhance phosphorylation of PKC, MEK, PKB/Akt, and GSK-3β	^620,621^
VAR10303	Iron	PD	6-OHDA-induced rat; MPTP treated mice	Selectively inhibit MAO-A and MAO-B affords iron chelating/iron-induced lipid peroxidation inhibitory	^622^
SK4	Iron	PD	MPP^+^ and 6-OHDA induced LUHMES cells	Transport iron to the brain via LAT1; Chelate brain iron	^623^
GW501516	Iron	PD	MPTP-induced mice; 6-OHDA-induced mice	PPARδ agonist; Prevent intracellular iron accumulation; Alleviate NLRP3 inflammasome-mediated neuroinflammation	^624,625^
α-Lipoic acid	Iron	PD	MPP^+^-induced PC12 cells	Activate PI3K/Akt/Nrf2 pathway	^626^
Para-aminosalicylic acid	Manganese	PD	Mn-induced Parkinsonism in humans	Reduce manganese levels	^627,628^
8a	Copper	AD	Cu^2+^-induced Aβ1–42 aggregation	Possess antioxidant activity; Inhibit Cu^2+^-induced Aβ1–42 aggregation	^657^
X1TMP X1Benz	Copper	AD	Cu^2+^-Aβ system	Diminish ROS generation	^658^
Curcumin	Iron Copper Zinc	AD	Aβ aggregation system	Chelate metal ions	^662^
CRANAD-17	Copper	AD	APP/PS1 transgenic mice	Inhibit Aβ 42 cross-linking induced by copper	^663^
(-)-epigallocatechin (EGC) (-)-epicatechin-3-gallate (ECG)	Copper Zinc	AD	Cu^2+^/Zn^2+^-Aβ40 induced N2a cells	Alleviate the toxicity of Aβ oligomers and fibrils	^664^
Eriodictyol,	Iron	AD	APP/PS1 mice; Aβ1–42 induced HT22 cells	Activate the Nrf2/HO-1 signaling pathway; Inhibit cell apoptosis	^666^
Amentoflavone,	Copper	AD	5xFAD mice	Diminish Cu^2+^-ascorbate redox cycling and ROS formation	^667^
Luteolin	Copper	AD	Copper-induced SH-SY5Y cells	Reduce Aβ secretion; Maintain mitochondrial function; Depress caspase-mediated apoptosis	^668^
apigenin	Copper	AD	APPsw cells	Attenuate β-amyloid neurotoxicity through antioxidation, mitochondrion protection and MAPK signal inactivation	^669^
vitegnoside	Copper	AD	Copper-treated SH-SY5Y cells	Inhibit p38 MAPK/JNK pathway	^670^
Silibinin	Iron Copper Zinc	AD	Intracerebral streptozotocin administration in mice; Mn-induced adult rats; Copper-induced rats; Zinc-induced rats;	Improve brain energy metabolism and cholinergic function; Reduce oxidative stress and inflammation	^672–675^
Berberine	Iron	AD	3 × Tg-AD mice	Inhibit iron levels and ferroptosis; Activate Nrf2 signaling	^682^
Coumarin	Copper Zinc	AD	Cu^2+^ and Zn^2+^ complexes system	Chelate metal ions; Inhibit cholinesterases or MAO-B	^683,684^
Naringin	Iron	AD	Iron-overloaded mice	Decrease nonheme iron; Reduce the formation of amyloid plaques	^685^
Tetrahydroxy stilbene glycoside	Iron	AD	APP/PS1 mice APP695V717I transgenic model mice	Activate GSH/GPX4/ROS and Keap1/Nrf2/ARE signaling pathway; Restore mitochondrial function; Inhibit ferroptosis;	^686,687^
1,6-*O*,*O*-diacetylbritannilactone (OABL)	Iron	AD	5xFAD mice; oxidative stress-induced PC12 cells	Inhibit oxytosis and ferroptosis; Suppress inflammatory	^688^
Ginkgolide B	Iron	AD	SAMP8 mice	Mitigate oxidative stress, neuroinflammation and ferroptosis	^689^
Hinokitiol	Iron	AD	RSL3-induced PC12 cells 6-OHDA-induced zebrafish	As a ferroptosis inhibitor	^690^
Senegenin	Iron	AD	Aβ25-35 induced PC12 cells	Reverse mitochondrial Depolarization; Inhibit ferroptosis	^691^
alkaloid extract from *African Jointfir* (*Gnetum africanum*)	Manganese	AD	Mn-induced Drosophila melanogaster	Inhibit AChE activity and ROS levels	^693^
Squaramide dipeptides	Copper Iron Zinc	AD	Aβ peptide system	Selectively chelate metal ions (Cu^2+^, Zn^2+^, and Fe^3+^) Exhibit antioxidant properties	^694^
Gly-His-Lys	Copper	AD	Cu^2+^-Aβ complex system	Suppress ROS production	^695^
7-*O*-cinnamoyltaxifolin; 7-*O*-feruloyltaxifolin	Iron	AD	RSL3/Glutamate/iodoacetic acid -induced HT22 cells; LPS-treated BV-2 microglia cells; Aβ25-35-induced mice	Inhibit oxytosis, ferroptosis and ATP depletion; Reduce neuroinflammation; Modulate Nrf2/GSH signaling pathway	^697^
Chromone-lipoic acid conjugate	Copper	AD	H_2_O_2_-induced cell damage in PC12 cells	Inhibit butyrylcholinesterase; Possess antioxidant and copper-chelation properties	^700^
SNH6	Iron Copper	AD	H_2_O_2_/copper-mediated SK-N-MC cells *C. elegans*	Enhance cellular NAD+/NADH ratios; Chelate iron; Inhibit copper-mediated Aβ aggregation	^701^
bis(7)-tacrine	Copper Iron	AD	AChE-induced amyloid-beta aggregation	Inhibit AChE; Chelate metals	^704^
Triethylene tetramine dihydrochloride	Copper	AD	APP/PS1 mice	Inhibit RAGE/NF-κB/BACE1 pathway	^705^
melatonin-trientine	Iron Copper	AD	APP/PS1 transgenic mice	Inhibit metal ion dyshomeostasis; Decrease Aβ deposition	^706^
Edaravone	Iron	AD	Aβ1–42-induced apoptosis of HT22 cells	Prevent TLR4/NF-κB /NLRP3 signaling pathway and ferroptosis	^718^
GIF-0726-r	Iron	AD	Glutamate/ erastin-induced HT22 cell	Inhibit ROS accumulation and Ca^2+^ influx	^720^
M30	Iron	AD	APP/PS1 Tg mice	Reduce cerebral iron accumulation; Decrease AD-like phenotypes	^721^
H2dqpyca and H2bqch	Copper	AD	Aβ peptide/H_2_O_2_-induced SH-SY5Y cells	Interact with AChE	^723^
salidroside	Iron	AD	SAMP8 mice	Activate Nrf2/GPX4 axis; Inhibit neuronal ferroptosis	^724^
β-hydroxybutyrate	Iron	AD	MPP^+^-induced SN4741 cells; MPTP-induced C57BL/6 mice	Alleviate oxidative stress and ferroptosis; Target ZFP36	^725^
IOE 12i	Copper Iron	AD	H_2_O_2_-induced PC12 cells	Chelate metal; Inhibit AChE; Inhibit ferroptosis, With BBB permeability	^728^
VU0063088 VU0026921	Manganese	PD/AD	pdat-1: GFP worms	Modulate manganese levels independent of SMF-2	^729^
MESM	Manganese	HD	MnCl_2-_treated murine striatal neuron lineage	Reduce excessive cellular manganese levels	^730^
Cu II(atsm)	Copper Zinc	ALS	SOD1^G37R^ mice; SOD1^G93A^ mice	Improve the metal content of mutant SOD1; Improve the lifespan and preserve motor neurons	^633,741–744^
ZnII(atsm)	Copper Zinc	ALS	SOD1^G37R^ mice	Increase the levels of copper in SOD1 and overall	^746^
ammonium tetrathiomolybdate	Copper	ALS	SOD1^G93A^ mice	Remove copper ions from the copper thiolate cluster of SOD1, leading to the degeneration of mutant SOD1	^747–749^
DP-109 and DP-460	Copper Zinc	ALS	SOD1^G93A^ mice	Chelate calcium, copper, and zinc; Improve motor performance	^751^
VK28, M30	Iron	ALS	SOD1^G93A^ mice	Chelate iron and prevent degeneration of motor neurons	^752^
PBT2	Copper Zinc	HD	R6/2 mice; C. elegans model of polyQ aggregation	Reduce toxicity; Extend lifespan; Reduce striatal atrophy;	^755^

##### Natural compounds and derivatives

Numerous natural compounds and their corresponding synthetic derivatives, which possess multifunctional neuroprotective properties, also exhibit an affinity towards metal ions. DL-3-n-butylphthalide, isolated from A*pium graveolens* seeds, has been approved for the treatment of acute ischemic stroke. A recent study demonstrated its ability to alleviate rotenone-induced motor impairment and dopaminergic neuron loss in rats. Notably, there was an improvement in both iron deposition within SN and serum iron levels, which is consistent with the observed alterations in the expression of the iron metabolism-related proteins, suggesting that DL-3-*n*-butylphthalide may have potential as a ferroptosis inhibitor.^603^ Paeoniflorin, a water-soluble monoterpene glycoside extracted from *Paeonia lactiflora Pall*, can prevent ferroptosis by activating the Akt/Nrf2/Gpx4 pathway in MPP^+^-induced primary dopaminergic neurons.^604^ Quercetin (QCT), a natural flavonoid with diverse pharmacological activities, exhibits potential anti-ferroptotic effects in erastin/RSL3 and MPP^+^-induced cells as well as MPTP-induced PD mouse model, primarily through the activation of Nrf2.^605^ Furthermore, QCT can attenuate copper-induced apoptotic cell death and ER stress in SH-SY5Y cells through autophagic modulation.^606^ Moreover, QCT effectively inhibited manganese-induced apoptosis and inflammatory response in SK-N-MC cells and SD rats, potentially involving the iNOS/NF-κB and HO-1/Nrf2 pathways.^607^ Epigallocatechin-3-gallate (EGCG) possesses a number of pharmacological activities and demonstrates potential neuroprotective properties in PD models. By acting as a metal chelator, EGCG attenuates the Fe^3+^-induced conformational transition of α-synuclein and protects AS-PC12 cells against Fe^3+^-induced death.^608^ It also exhibits a neurorescue effect in both a mouse model and a *Drosophila* model of PD.^609,610^ Inhibition of ferroptosis was identified as the underlying mechanism of EGCG-mediated protection.^610^ Furthermore, EGCG can bind to metal ions [Cu^2+^ and Zn^2+^]-Aβ monomers and dimers, resulting in compact peptide conformations and the formation of ternary EGCG-metal-Aβ complexes.^611^ Emodin, a natural anthraquinone derivative, also inhibits zinc-induced neurotoxicity in SH-SY5Y cells.^612^ Thonningianin A, a polyphenolic compound found in natural plant foods, attenuated ferroptosis induced by 6-OHDA in zebrafish and dopaminergic neurons. It exerts its effects by targeting the Kelch domain of Keap1 and promoting its degradation in an Atg7-dependent manner, leading to the translocation of Nrf2 into nucleus, suggesting it as a Keap1-Nrf2 PPI inhibitor to inhibit ferroptosis.^613^ (-)-Clausenamide Clau, an alkaloid isolated from the plant *Clausena lansium* (Lour.), exhibits both in vivo and in vitro neuroprotective activities as a scavenger of lipid peroxide products. Data analysis reveals that (-)-Clausenamide directly targets Ser663 on ALOX5, which is the PKCα-phosphorylation site.^614^ The results propose a new idea that targeting ALOX5 and preventing ferroptosis in dopaminergic neurons may be a strategy for PD therapy. Some extracts have also shown protective roles against metal-induced toxicity. Oral administration of *E. amoenum* extract increased catecholamine content and improved depression-like behavior in the Mn^2+^-treated hippocampus of rats.^615^ *Anthocyanin-rich açaí* (*Euterpe oleracea Mart*.) extract attenuates manganese-induced oxidative stress in primary astrocyte cultures of rats.^616^ The polyphenolic extract of *Euphorbia supina* could enhance antioxidant activity by regulating ER stress and mediating apoptosis to attenuate manganese-induced neurotoxicity.^617^ The similar effects were also observed in the extract of *Melissa officinalis aqueous* and *Peumus boldus* (Boldo) *Aqueous* e in manganese-injured mice or D. melanogaster.^618,619^

##### Multifunctional iron chelators

In recent years, a series of multifunctional iron chelators has been reported. One such example is the multimodal iron chelating drug M30, which has been shown to facilitate numerous neuroprotective-adaptive mechanisms and pro-survival signaling pathways. It has also been reported to increase striatal dopamine levels and enhance dopaminergic and transferrin receptor cell populations in the SNpc.^620,621^ Recently, Bar-Am et al. synthesized a novel multipotent compound called VAR10303, which possesses brain-permeable iron-chelation properties. This compound demonstrated significant improvement in PD symptoms in rat models induced by 6-OHDA as well as mouse models treated with MPTP.^622^ Gutbier et al. designed a series of small molecules capable of transporting iron to the brain *via* the neutral amino acid transporter, LAT1 (SLC7A5), which was found to effectively block neurite loss and cell death induced by MPP^+^ and 6-OHDA in LUHMES cells.^623^ In an MPTP-induced PD mouse model, GW501516, known as a PPARδ agonist, showed potential for alleviating motor impairment and reducing NLRP3 inflammasomes-mediated neuroinflammation.^624^ Furthermore, it was discovered that it regulates the expression of FPN1 and ferritin, while modulating cellular iron homeostasis without affecting TfR1 in the context of 6-OHDA.^625^ Additionally, α-Lipoic acid has been reported to decrease the occurrence of neuropathy, and it is possible that the PI3K/Akt/Nrf2 pathway could serve as a potential target.^626^ In the context of severe manganese-induced Parkinsonism in humans, Para-aminosalicylic acid (PAS), an FDA-approved anti-tuberculosis drug, has demonstrated neuroprotective effects. PAS effectively reduced manganese levels in the striatum, hippocampus, and frontal cortex of Sprague-Dawley rats, along with iron and copper levels.^627,628^

#### Clinical trials targeting metal ions

In certain clinical practices for the treatment of PD, chelators have demonstrated favorable efficacy. DFP, as a membrane-permeant bidentate chelator, has shown neuroprotective effects in both experimental models and clinical trials of PD (Table 3). DFP effectively reduces labile iron levels and attenuates oxidation products of lipids and DNA in cells and animals subjected to oxidative stress, leading to improvements in motor functions while mitigating dopamine depletion.^15^ A case report revealed that DFP treatment (30 mg/kg/day for 32 months) resulted in decreased iron accumulation in the bilateral dentate nuclei and SN of a PD patient with dysarthria and orofacial dystonia, as well as iron accumulation across various brain regions including the dentate nuclei, SN, and red nuclei.^629^ Despite significant improvement in iron overload, the patient’s best T2* values had not fully recovered. A double-blind placebo clinical trial involving 40 patients was conducted to evaluate the efficacy and safety of DFP (30 mg/kg/day).^15^ The results indicated that DFP significantly reduced iron content in the SN, with slight improvement observed in motor signs after 1 year of treatment. Although three cases of neutropenia occurred, symptoms rapidly disappeared upon discontinuation of oral therapy (ClinicalTrials.gov reference: NCT00943748). Another study also demonstrated that DFP therapy effectively suppressed iron accumulation in specific brain regions in PD patients for 6 months (either 20 or 30 mg/kg), with the exception of two patients who developed neutropenia (ClinicalTrials.gov reference: NCT01539837).^630^ Iron chelation may serve as a potential alternative treatment option for individuals suffering from neurodegeneration associated with brain iron accumulation. However, it is important to consider the significant decrease in plasma ferritin levels following long-term treatment, as this could potentially lead to body iron depletion and subsequent iron deficiency. Therefore, when evaluating the positive effects of chelators, one must carefully assess the risk of agranulocytosis and determine an optimal dosage.^629,630^ Additionally, Grolez et al. demonstrated that patients with lower Cp activity displayed a more favorable response to iron chelation therapy (ClinicalTrials.gov reference: NCT00943748).^282^ However, it is crucial to conduct large-scale trials in order to establish the relationship between Cp activity and PD. A FAIRPARK-II clinical trial of DFP investigated the use of DFP in early PD patients and revealed that no clinical benefit from this therapy; instead, it resulted in a significant decrease in MDS-UPDRS scores at 9 months and worse scores on measures of Parkinsonism compared to those receiving placebo over a 36-week period (ClinicalTrials.gov reference: NCT02655315).^631^ These findings suggest that caution should be exercised when considering iron-chelating agents as potential therapeutic interventions for PD.

**Table 3:** Clinical trials targeting metal ions in neurodegenerative diseases

Agents	Trial identifier	Action mechanism	Output	Disease	Ref
DFO (intramuscular)	/	Iron chelation	Reduction in the rate of decline of daily living skills with 125 mg twice daily, 5 days per week	AD	^26^
DFP (oral)	NCT00943748	Iron chelation	Three points on the UPDRS part III scale compared with placebo at 30 mg/kg/day	PD	^15^
	NCT02655315	Iron chelation	Worse scores in measures of parkinsonism than those with a placebo over a period of 36 weeks.	PD	^631^
	NCT01539837	Iron chelation	A trend for improvement in motor-UPDRS scores and quality of life; Cognitive function and mood were not improved.	PD	^767^
	NCT03234686	Iron chelation	Underway, outcomes include performance on a neuropsychological test battery and brain iron levels as measured by MRI.	AD	
	NCT 00868166	Iron chelation	Two points on the ALSFRS-R scale at 30 mg/kg/day	ALS	^740^
Curcumin	NCT04149639 NCT01716637 NCT01001637 NCT00164749 NCT00099710	Iron chelation	No significant differences in changes in cognitive function was found between the placebo and curcumin groups	AD	^731,733,734^
PBT2	NCT01590888	Copper/zinc/ iron chelation	The Trail Making Test Part B score was improved in the PBT2 250 mg group; No significantly improved cognition on the other tests	HD	^756^
	NCT00471211	Copper/zinc/iron chelation	Marked improvement on putative biomarkers for AD in CSF but not in plasma; Executive function	AD	^735^
	NCT00471211	Copper/zinc/iron chelation	An improver from the PBT2 250 mg group for Composite z-scores, Executive Factor z-scores, and near-significant for the ADAS-cog	AD	^736^
GSH (intranasally)	NCT02424708	GSH Increase	The improvement in the placebo arm was more robust than has been observed in previous PD studies	PD	^636^
NAc (oral)	NCT02445651	GSH Increase	UPDRS scores were also significantly improved	PD	^768^
CuII(atsm)	NCT04082832	Copper chelation	No significant alleviation in neuronal pathology or astrogliosis in patients	ALS	^745^
	NCT02870634	Copper chelation	Disease severity and cognitive function relative to historical controls were improved in both phase 1 and phase 2a cohorts.	ALS	

CuII(atsm) exhibited neuroprotective effects in four distinct animal models of PD by enhancing dopamine metabolism and restoring cognitive and motor function. In vitro, it demonstrated the inhibition of peroxynitrite-mediated formation of α-synuclein oligomers.^632^ Encouraging results from separate Phase 1 studies in patients with ALS and PD indicate that CuII(atsm) inhibits Fe^2+^-induced lipid peroxidation, suggesting its potential as a disease-modifying mechanism against ferroptosis.^633^ With comparable potency to liproxstatin-1 in vitro, CuII(atsm) possesses favorable properties such as oral bioavailability and brain penetration, which makes it an attractive investigational product for clinical trials targeting ferroptosis-related diseases.^634^ Additionally, *N*-acetylcysteine (NAC) and GSH have shown improved clinical symptoms in PD patients during clinical trials (ClinicalTrials.gov reference: NCT02424708; NCT02445651).^635,636^ Omega fatty acids and CoQ_10_ have demonstrated some efficacy in treating PD patients^637,638^; however, the therapeutic response depends on baseline levels of ubiquinol and the dosage of CoQ_10_ (ClinicalTrials.gov reference: NCT00740714). Furthermore, vitamin E co-supplemented with omega-3 fatty acids has also been found to have favorable effects on UPDRS and markers of insulin metabolism in individuals with PD.^639^ Limited treatment options are available for manganese-induced neurotoxicity; chelation therapy is preferred. Calcium disodium ethylenediaminetetraacetate (CaNa_2_EDTA), which replaces toxic metals with calcium within the EDTA core, thereby inhibiting dopaminergic auto-oxidation, has been reported to improve seven workers affected by manganese-induced Parkinsonism.^640,641^ However, controversial results were observed due to poor BBB permeability leading to low bioavailability and efficacy.^628^

### AD

#### Metal ions chelation

##### DFO and CQ derivative

The dysregulation of metal homeostasis contributes to the pathogenesis of AD. Therefore, metal chelators that can reduce extracellular metal bioavailability or compete with endogenous ligands for metal ions may be a beneficial choice in the treatment of AD^642^ (Table 2). Intranasal DFO has been reported to reduce cognitive decline and inhibit amyloid beta deposits in P301L tau transgenic mice^643^ and APP/PS1 mice.^355,644^ Intranasal DFO can act on multiple processes to slow disease progression, with the most significant improvements observed in reduced Aβ aggregation and hyperphosphorylation of tau.^355^ DFO is not only a metal-chelator that suppress ferroptosis, including iron-associated oxidative stress,^645^ but also a potent inhibitor of GSK-3β, which may connect Aβ accumulation and subsequent tau hyperphosphorylation in AD.^643,644^ DFO also targets HIF-1α to activate glucose transporters, including glucose transporter 1 (GLUT1), and plays a role in the activation of insulin signaling pathway.^646–648^ The *N*-acylhydrazones group is a precursor for many compounds with chelating properties. An example of such a compound is 8-hydroxyquinoline (8-HQ), which is a natural lipophilic molecule that exhibits strong anti-AD effects by chelating metal ions.^649^ Based on the structural framework of 8-HQ, a series of nitroxoline-based analogs were designed, including 8- H2QH, 8-H2QT, and 8-H2QS. These compounds exhibited inhibitory effects on self-induced aggregation of the amyloid beta peptide (Aβ1–42) and selectively formed complexes with Cu^2+^.^597,650,651^ CQ is a nonspecific copper-zinc chelator derived from 8-HQ, which not only improves cognitive behavior and decreases Aβ deposits in an APP transgenic mouse model of AD,^652^ but also prevents age-dependent brain atrophy, iron accumulation, and concomitant cognitive deficits in tau-knockout mice.^653^ Another mechanism of CQ against AD involves the resumption of Cu^2+^-suppressed fibril growth of Aβ (1–40). Interestingly, when combined with Zn^2+^ ions, it synergistically retards fibril growth.^654^ Furthermore, the combination therapy involving CQ and dihydropyrimidine-thiones demonstrates enhanced amelioration of Aβ toxicity by reducing Aβ levels and restoring functional vesicle trafficking, highlighting the potential value of synergistic compounds for enhancing targeted activities.^655^ Novel polymeric derivatives based on the structure of 8-HQ were designed with N and O donor atoms similar to those present in CQ, and these derivatives exhibit a superior capacity for disaggregating Aβ plaques due to their strong affinity for copper ions.^656^ The structure of CQ was used as a basis for synthesizing a series of selenium-containing derivatives. Importantly, compound 8a exhibited predominant antioxidant activity and effectively inhibited Cu^2+^-induced Aβ1–42 aggregation, suggesting its potential as a multifunctional agent for treating AD.^657^ The compounds X1TMP and X1Benz, which possess the ability to diminish ROS generation by the copper^2+^-Aβ system, exhibit promising potential against Aβ fibril formation in AD.^658^ Additionally, a series of adamantane-based semicarbazones and hydrazones compounds were designed and demonstrated as novel therapeutic agents by inhibiting Cu^2+^-mediated Aβ aggregation and effectively chelating iron.^659^ Furthermore, certain synthetic hydrazone ligands presented efficient inhibition of Aβ fragments aggregation and efficiently chelated copper and zinc.^660^

##### Polyphenols compounds

Besides, polyphenols have been proven to be effective in preventing the death of dopaminergic neurons. Curcumin has been reported to reduce the aggregation of amyloid and oxidized proteins, as well as improve cognitive deficits. As a metal chelating agent, its alternative mechanism may involve reducing metal ions to prevent Aβ amyloid fibril formation.^661^ Curcumin displayed different affinity for copper, zinc, and iron. At least two curcumin molecules can bind to Cu^2+^ or Fe^2+^ ion, and exhibited positive or negative cooperativity, with little bind to Zn^2+,^.^662^ A curcumin analog named CRANAD-17, which utilizes curcumin as an anchoring moiety to H13 and H14 of Aβ, has the capability to inhibit copper-induced cross-linking of Aβ42, suggesting that the curcumin scaffold holds potential as a tool for AD research.^663^ (-)-epigallocatechin (EGC) and (-)-epicatechin-3-gallate (ECG), the main polyphenolic compounds derived from tea, have been found to alleviate the toxicity of Aβ oligomers and fibrils induced by Cu^2+^ or Zn^2+^.^664^ Mandel et al. proposed the use of a cocktail of multimodal brain-permeable iron-chelating drugs that possess neuroprotective-neurorescue and APP-processing regulatory activities as therapeutic agents, with a specific focus on EGCG and curcumin.^665^

##### Flavonoids compounds

Flavonoids, a class of polyphenolic compounds, have been shown to independently chelate metal ions and interact with Aβ, indicating their potential bifunctionality toward metal–Aβ species. Eriodictyol, a natural flavonoid compound, effectively ameliorated cognitive deficits in APP/PS1 mice and suppressed Tau phosphorylation in Aβ1–42 induced HT22 cells and APP/PS1 mice. Moreover, eriodictyol exerted an anti-ferroptosis effect by activating the Nrf2/HO-1 signaling pathway.^666^ The biflavonoid compound mentoflavone, which possesses good metal-chelating properties, can regulate the formation of neurotoxic soluble Aβ42 oligomers and reduce the Cu^2+^-ascorbate redox cycling and ROS formation.^667^ Luteolin, derived from *Elsholtzia rugulosa*, exhibits inhibitory effects on copper-induced neurotoxicity in SH-SY5Y cells overexpressing APP by reducing Aβ secretion, maintaining mitochondrial function, and suppressing the caspase-mediated apoptosis.^668^ Similar effects were observed with apigenin and vitegnoside.^669,670^ Myricetin has been identified as another natural regulator of metal-induced Aβ aggregation and neurotoxicity.^671^ Silibinin, a clinically used hepatoprotectant isolated from *Silybum marinum*, attenuates memory impairment induced by Aβ by reducing oxidative stress and inflammation in the mouse brain.^672^ It has been reported that silymarin, delivered through silymarin-encapsulated liposome nanoparticles, can protect against zinc-induced dopaminergic neurodegeneration, improve spatial memory, and reduce depression induced by copper.^673,674^ Additionally, silymarin protects the cerebral cortex against manganese-induced neurotoxicity in adult rats.^675^ Due to three potential metal-binding sites mediated by the catechol moieties, quercetin possesses natural chelating activity, which can combat copper-induced toxicity in SH-SY5Y cells by regulating apoptotic cell death and ER stress involving PI3K/Akt and ERK1/2 signaling.^606,676^ Liu et al. developed a nanomedicine by modifying quercetin with triphenylphosphonium (TQCN), which efficiently chelates iron to ameliorate various neurodegenerative manifestations associated with brain iron deposition, thus rescuing severe cognitive decline in AD mice and demonstrating the promising potential of intelligent nanotherapeutics against AD progression.^677^ Sterubin and fisetin, two flavonoids derived from natural pharmacopeia, have shown strong inhibition on oxytosis/ferroptosis. Recently, it has been reported that sterubin and fisetin have diverse homeostatic impacts on mitochondrial physiology. Although they can enhance bioenergetic efficiency by restoring mitochondrial homeostasis in terms of redox regulation, calcium uptake, biogenesis, fusion/fission dynamics, and modulation of respiration, they do not require mitochondria to exert neuroprotective effects.^678^ The intricate interplay and targets should be evaluated when considering these compounds as agents. The assessment of structure-activity relationships revealed specific binding sites for metal ions, including the catechol moiety in the B-ring, the 3-hydroxy and 4-oxo groups in the C-ring, and the 4-oxo and 5-hydroxyl groups between the C- and A-rings.^679^ Genistein-*O*-alkylbenzylamine derivatives emerged as Cu^2+^ chelators and significantly inhibited copper-induced Aβ aggregation.^680^ Another noteworthy example based on flavonoids is FLV2 5, which exhibited copper chelating properties along with enhanced AChE inhibitory activity and inhibition of self-induced Aβ aggregation.^681^

##### Alkaloids and terpene compounds

Several other natural alkaloids, lactones and terpenes with neuroprotective effects also demonstrate the ability to chelate metal ions. Berberine, a natural alkaloid, exhibited neuroprotective value in triple transgenic AD mice by inhibiting iron levels and ferroptosis while activating Nrf2 signaling in RSL3-induced ferroptosis.^682^ Recent reports have found that coumarin and its analogs and derivatives possess many properties targeting AD, such as chelation of metal ions, inhibition of cholinesterases (ChEs) or monoamine oxidase B, proposing coumarin as a potential scaffold for improving the structure of anti-AD therapeutics.^683,684^ Naringin has been reported to efficiently decrease nonheme iron, thereby reducing the formation of amyloid plaques in iron-overloaded mice.^685^ Tetrahydroxy stilbene glycoside (TSG) dose-dependently mitigates Aβ-induced brain damage by activating GSH/GPX4/ROS and Keap1/Nrf2/ARE signaling pathways. Additionally, TSG restores mitochondrial function to reverse Aβ-induced injury, suggesting its potential as a multifunctional candidate against AD.^686^ Qian et al. synthesized a derivative of TSG, called Mito-TSG, and demonstrated its superior effects in mitigating mitochondrial free radical damage and apoptosis in APP695V717I transgenic model mice.^687^ 1, 6-*O*, *O*-diacetylbritannilactone (OABL), a 1,10-seco-eudesmane sesquiterpene lactone isolated from the herb *Inula britannica L*., attenuates the impairments in cognitive function and the overactivation of microglia and astrocytes in the brains of 5xFAD mice, which also inhibits oxytosis and ferroptosis in PC12 cells.^688^ Ginkgolide B, a terpene lactone derivative of *Ginkgo biloba*, also alleviates AD-induced cognitive defects by mitigating oxidative stress, neuroinflammation, and ferroptosis, and the inhibition of ferroptosis has been found to be essential for the beneficial effects of Ginkgolide B in AD using SAMP8 mice as models.^689^ Hinokitiol, a potent small molecule inhibitor of ferroptosis, dynamically transports iron from areas with high-iron concentration to those with low concentration in order to dissipate iron buildup. It has been reported to alleviate neurotoxicity and rescue the neurobehavioral impairment caused by paclitaxel or 6-OHDA in vitro and in vivo.^690^ Senegenin (Sen), the major component of *Radix Polygala*, exhibits diverse pharmacological activities in neurodegenerative disorders by enhancing cognitive function, exerting anti-aging effects, and acting as an antioxidant. It effectively reverses mitochondrial depolarization in Aβ25–35-induced PC12 cells, demonstrating potent neuroprotective properties against oxidative damage and lipid metabolite associated with ferroptosis.^691^ Considering the necessity for specific metal-ion binding affinity coupled with antioxidant functionalities, yclen (1) acts as a metal ion passivation and antioxidant agent to prevent and disrupt copper-induced aggregation of Aβ (1-40).^692^ Furthermore, the alkaloid extract from *African Jointfir* (*Gnetum africanum*) leaves counteracted manganese-induced elevation in acetylcholinesterase enzyme (AChE) activity, as well as levels of nitric oxide (NO) and ROS, in *Drosophila* melanogaster.^693^

##### Amino acids and polypeptides

The α-carbon of amino acids, such as methionine, tyrosine, histidine, and cysteine, can chelate metals to form metal complexes through their amine and carboxylate moieties. As a result, peptides composed of these amino acids also possess the ability to chelate metals. A notable example is the histidine-bearing dipeptide. Additionally, certain peptides have been identified as potent inhibitors of Aβ peptide aggregation, including tryptophantyrosine, glycine-arginine dipeptide, methioninetryptophan dipeptide, muramyl dipeptide, and hydroxyethylene-containing diphenyl alaninedipeptide.^694^ In a study by Suchita et al., bifunctional backbone-modified squaramide dipeptides were synthesized, which have the ability to inhibit Aβ aggregation and selectively chelate metal ions (Cu^2+^, Zn^2+^, and Fe^3+^).^694^ Additionally, these dipeptides exhibit antioxidant properties. Therefore, they hold potential as novel chemical entities for AD treatment. GHK (Gly-His-Lys), a bioactive matricryptin released from extracellular proteins *via* limited proteolysis, suppresses ROS production catalyzed by the copper-Aβ complex system.^695^ Its conjugate (BioGHK) exhibits similar effects while also displaying antiglycating activity toward carbonylated species Aβ. Asadbegi et al.’s research led to the discovery of a novel multifunctional ligand that can simultaneously inhibit Aβ42 and chelation zinc.^696^ The inhibitor domain binds to the C-terminal hydrophobic region of Aβ while another domain is responsible for zinc chelation, as confirmed by molecular docking and molecular dynamics evaluation.

##### Compounds based on lead compounds

The lead compounds were modified to enhance their metal chelating effect, leading to the development of numerous new compounds. Gunesch et al. synthesized a series of 7-O-Esters of taxifolin, named 7-*O*-cinnamoyltaxifolin and 7-*O*-feruloyltaxifolin, which exhibits significant synergistic neuroprotective effects in an HT22 cell model by effectively inhibiting ferroptosis.^697^ Additionally, administration of these compounds improved short-term memory in an AD mouse model induced by oligomerized Aβ25–35 peptides, potentially through modulation of Nrf2/GSH signaling as a therapeutic target. Three catechol-based ligands demonstrated selective chelation abilities towards redox-active metal ions, particularly Cu^2+^ and Fe^3+^, with a lower affinity for Zn^2+^.^698^ These ligands effectively inhibited the reduction of dioxygen of Cu-II-Aβ (1–16). To investigate the role of metal-Aβ interactions in AD pathogenesis, ligands were designed that incorporate a metal binding site into a framework known to interact with Aβ.^699^ These ligands have been shown to attenuate ROS generation and mitigate oxidative stress. Based on natural-origin compound scaffolds, several multitarget compounds have been designed and applied for the treatment of various neurodegenerative diseases. Among them, chromone-lipoic acid conjugate exhibits acceptable butyrylcholinesterase inhibition activity, as well as antioxidant and copper-chelation properties, making it a promising anti-AD agent.^700^ SNH6, a hydrazone of 6-methoxysalicylaldehyde nicotinoyl, exhibits significant inhibition of copper-mediated Aβ aggregation and effectively donated NAD^+^ to NAD-dependent metabolic processes.^701^ Additionally, it demonstrated efficacy in chelation iron, suggesting its potential as a multifunctional precursor for AD treatment. The multitarget-directed ligands (MTDLs) strategy was employed to synthesize a class of chrysin derivatives. These compounds demonstrated effective inhibition of self-, Cu^2+^-, and AChE-induced Aβ1–42 aggregation, with favorable BBB penetration and predicted drug-like properties in silico. Notably, compound 1 exhibited highly selective inhibition of butyrylcholinesterase (BuChE).^702,703^ Bolognesi et al. modified a lead compound bis(7)-tacrine and endowed it with triple function as a dual-binding site AChE inhibitor and chelating metals, which presented inhibited effects on the AChE-induced Aβ aggregation.^704^ Triethylene tetramine dihydrochloride (trientine), a selective copper chelator, was found to mitigate amyloidosis in the brain of APP/PS1 mice by inhibiting the RAGE/NF-κB/BACE1 pathway.^705^ Subsequently, Li et al. designed a novel compound named melatonin-trientine by covalently synthesizing melatonin with trientine. The compound effectively maximized the therapeutic efficacy by targeting multiple mechanisms associated with AD pathology by not only inhibiting metal ion dyshomeostasis but also decreasing Aβ deposition.^706^ Resveratrol, a multitarget compound, exerts an anti-apoptotic effect and reduces Aβ and SOD1 aggregation through its antioxidant activity and mitochondrial protection.^707,708^ By virtue of its stilbene structure, a series of imine resveratrol derivatives was synthesized with the ability to chelate copper and inhibit Aβ aggregation.^709^ Tenuazonic acid and its conjugates with donepezil were shown to be a potent chelator of Fe^3+^, Cu^2+^, and Zn^2+^ with the potential effects on AChE and Aβ aggregation inhibition, proving to be noncytotoxic against SH-SY5Y cells.^710^ Another group of mental chelators reported by Kilic et al. includes a series of thiourea and benzamide derivatives that exhibit ChEs inhibition.^711^ Results from a pilot placebo-controlled trial on AD patients revealed that although d-penicillamine, a copper-chelating agent, decreased oxidative stress, no difference was observed in the rate of cognitive decline.^712^ Further studies involving larger cohorts are required to elucidate the drug’s efficacy in relation to the clinical progression of AD. Synthesized hydroxylated chalcones with dual-functional inhibitors targeting Aβ aggregation and ferroptosis, along which chalcones 14a-c exhibited a more significant neuroprotection against Aβ1–42 induced toxicity.^713^ The compounds also demonstrated favorable ADMET properties and BBB penetration. Iminodiacetic acid-modified human serum albumin (I-HSA) effectively inhibits Zn^2+^ and Cu^2+^-associated Aβ42 aggregation, making it the first multifunctional macromolecule for reducing metal-induced Aβ42 aggregation.^714^ This finding provides valuable insights for the development of macromolecular drugs targeting AD. Furthermore, a series of DFP derivatives has been synthesized as dual-binding site AChE inhibitors, allowing simultaneous interaction with catalytic active site and peripheral anionic site of the enzyme, and these compounds chelate bio-metals co-localized in Aβ plaques, such as iron, in some cases with copper and zinc.^715^

##### Other compounds

Edaravone, a drug targeted mitochondria and recognized for its ability to scavenge hydroxyl radicals (∙OH) and inhibit lipid peroxidation, has emerged as a promising therapeutic agent against various neurological disorders.^716,717^ Recently, it has been demonstrated that it can reduce Aβ1–42-induced apoptosis of HT22 cells by inhibiting the TLR4/NF-κB /NLRP3 signaling pathway and ferroptosis.^718^ Additionally, the compound 1,1’-xylyl bis-1,4,8,11-tetraaza cyclotetradecane was reported as an effective drug for clearing Aβ by specifically reducing the transcription of APP and targeting copper concentration in the brain cortex region.^719^ In a search for neuroprotective agents, GIF-0726-r, an oxindole compound, presented neuroprotective roles against glutamate-induced oxytosis and erastin-induced ferroptosis in the hippocampal HT22 cell line. Additionally, GIF-0726-r was found to effectively inhibit the accumulation of ROS and Ca^2+^ influx. Several synthesized derivatives of GIF-0726-r, were identified as potent activators of the antioxidant response element.^720^ Many compounds with positive treated effects also exhibit anti-AD roles, such as M30, which can effectively reduce Aβ accumulation and tau phosphorylation, and alleviate memory deficits.^721,722^ The metal chelator H2dqpyca and H2bqch exhibited significant neuroprotection against both Aβ peptide- and H_2_O_2_-induced toxicities in SH-SY5Y cells, indicating a strong interaction with the catalytic active site of AChE.^723^ The administration of salidroside alleviated cognitive impairment and inhibited neuronal ferroptosis in SAMP8 mice, possibly through activating Nrf2/GPX4 axis and reducing the infiltration of CD8 + T cells.^724^ β-hydroxybutyrate (BHB) alleviated oxidative stress and ferroptosis induced by MPP^+^/MPTP in SN4741 cells and C57BL/6 mice by modulating zinc finger protein 36 (ZFP36)/ACSL4 axis, with ZFP36 being identified as a target of BHB.^725^ Virtual screening technology has been recognized and applied in the screening targeted compounds.^726^ Soriano-Castell et al. discovered a small set of anti-oxytotic/ferroptotic quinones from a comprehensive library of over 900 natural compounds, which protect against intracellular Aβ toxicity in nerve cells by modulating quinone oxidoreductase 1 (NQO1) and ferroptosis suppressor protein 1 (FSP1).^727^ An imidazolylacetophenone oxime ether (IOE) was discovered against neuroinflammation based on the screening (∼1400 compounds). Its derivatives, specifically compound 12i, exhibited prominent metal-chelating and inhibition of AChE properties.^728^ It inhibited H_2_O_2_-induced PC12 cells damage and ferroptosis, while also showing potential for chelating copper with BBB permeability. Two small molecules, VU0063088 and VU0026921, were selected from a high throughput screen of 40,167 compounds, which play protective roles against manganese toxicity in *C. elegans* by modulating manganese levels in the worms independently of SMF-2, a regulator of manganese transport.^729^ Another molecule, known as MESM, has been identified as a manganese-selective ionophore, which can effectively reduce excessive cellular manganese without altering levels of calcium, iron, cobalt, copper, zinc or molybdenum.^730^ This discovery holds promise for patients with elevated manganese levels.

#### Clinical trials targeting metal ions

A large number of metal chelator compounds have been developed to treat AD; however, only a few of them have progressed to clinical trials. The clinical trials involving metal chelated therapeutics are presented in Table 3.

Curcumin not only inhibits Aβ aggregation and the activities of β-secretase in copper-induced cells, but also decreases Aβ deposition and oligomer formation, reduces tau phosphorylation, and improves behavioral impairment in AD animal models.^731^ The potential mechanisms may be related to oxidative stress and mitochondrial apoptosis.^732^ Chelation with copper and iron suggests one possible mechanism of action in AD animal models.^662^ Currently, several clinical trials investigating the effects of curcumin on AD has been conducted (ClinicalTrials.gov reference: NCT04149639; NCT01716637; NCT01001637; NCT00164749; NCT00099710).^731,733,734^ However, no significant differences in changes in cognitive function were found between placebo and curcumin groups. Additional trials are necessary to evaluate the roles in AD, while considering its bioavailability and ability to penetrate the BBB. Based on the structure-activity relationship, many curcumin derivatives have been designed to potentially play a more effective role.^709^ A 2-year, single-blind study showed that 24 months of treatment with DFO (125 mg intramuscularly twice daily, 5 days per week) reduced the rate of decline in daily living skills of 48 patients with probable AD.^26^ Slight side effects, including weight (1/48) loss and appetite (4/48) were observed. An ongoing clinical trial (NCT03234686) on DFP in AD has yielded several outcomes, including performance on a neuropsychological test battery and measurement of brain iron levels using MRI. PBT2 (5, 7-dichloro-2-(dimethylamino)-methyl-8-hydroxyquinoline), a derivative of CQ, is in clinical trials for the treatment of AD and HD with well safety and tolerability (ClinicalTrials.gov reference: NCT00471211 and NCT01590888). It exerts a central effect on amyloid plaque metabolism by binding excesses of copper and zinc, possibly iron, to inhibit the formation of toxic oligomers. Patients treated with PBT2 at a dose of 250 mg showed significant improvement in two executive functions and a dose-dependent reduction in CSF Aβ concentration.^630,735,736^ Despite PBT2 appearing to improve cognitive function in animal models and in clinical trials, it did not have a significant effect on the burden of amyloid plaque in larger-scale clinical trials.^737^ Furthermore, CQ exhibited severe neurotoxic side effects, and PBT2 did not meet the predetermined endpoints; therefore, the clinical trial was terminated. The hydroxyl group and the nitrogen atom was postulated as the important mental binding.^738^ Subsequently, several other copper chelators derived from CQ have been developed based on the ion properties and structure-activity relationship. Among them, bis-8-hydroxyquinoline derivatives (PA1637, with undisclosed structure) showed superior therapeutic effects and significantly lower toxicity compared to CQ in an AD mouse model; further clinical trials are required to validate its effects.^597,739^

### ALS

Iron accumulation has been observed in animal models and in both sporadic and familial forms of ALS patients. DFP was reported to extend the mean lifespan of Sod1G86R mice compared with placebo. In a pilot clinical trial lasting 12 months (ClinicalTrials.gov reference: NCT 00868166), DFP has been proven to be good safety without anemia. Additionally, DFP treatment (30 mg/kg/day) for the first 3 months significantly improved the ALS Functional Rating Scale and body mass index compared to the initial treatment-free period. After DFP treatment, iron levels in multiple sites and oxidative stress in CSF decreased, suggesting that moderate iron chelation may be a novel therapeutic approach for neuroprotection in ALS.^740^ It was reported that oral treatment with CuII(atsm) improved locomotor function and the survival of the SOD1^G37R^ mice through improving the metal content of mutant SOD1 in vivo, rather than reducing levels of misfolded SOD1.^633,741,742^ Furthermore, administration of CuII(atsm) showed improvements on the lifespan and preservation of motor neurons in transgenic SOD1^G93A^ mice, and the activation of astrocyte and microglial was also been attenuated.^743,744^ A clinical trial (ClinicalTrials.gov reference: NCT02870634), completed in 2020, demonstrated that disease severity and cognitive function relative to historical controls were improved after treatment with CuII(atsm) in both phase 1 and phase 2a cohorts. However, another clinical trial (ClinicalTrials.gov reference: NCT04082832) found that CuII(atsm) has no significant effect on motor neuronal pathology in patients with ALS.^745^ Therefore, further studies are needed to evaluate the effects of CuII(atsm). In another study, the SOD1-G73R mice treated with ZnII(atsm) presented similar improvements in locomotor and survival. Interestingly, this treatment did not affect the content of zinc levels, but increased the levels of copper in SOD1 and overall, suggesting that the neuroprotective effects of ZnII(atsm) may in fact be due to subsequent delivery of copper.^746^ These findings suggest that improving copper content represents a promising therapeutic strategy for treating ALS. In contrast, many copper-chelating drugs have been reported to be beneficial in mutant SOD1 mouse models. For example, ammonium tetrathiomolybdate (TTM), a copper chelator, prolonged the survival of both presymptomatic and symptomatic SOD1-G93A mice by removing copper from the copper thiolate cluster of SOD1, which led to the degeneration of mutant SOD1.^747–749^ Tardiff et al. found that different 8-HQ protect models of TDP-43 protein, α-synuclein, and polyglutamine proteotoxicity through distinct mechanisms.^750^ HQ-415 and CQ exhibited stronger chelation activities than HQ-161. Each model can rely on at least two metal chelation pathways to be involved.^750^ The lipophilic metal chelators DP-109 and DP-460, which chelate calcium, copper, and zinc, significantly improved motor performance, significantly extended survival, and dramatically reduced cell loss in the lumbar spinal cord of G93A-transgenic ALS mouse model.^751^ Brain-permeable iron chelators VK28 and M30 have shown to prevent motor neuron degeneration in SOD1^G93A^ transgenic mice of ALS.^752^ Additionally, other chelators such as metformin and cyclodipeptides have been effectively applied to treat neurodegenerative diseases, including AD, despite being originally designed for other purposes.^683,753^

### HD

Many metal chelator compounds also exhibit neuroprotective effects and resistance against HD. CQ, a metal-binding compound, decreased the expression of mutant protein with olyglutamine-expanded huntingtin exon 1 in PC12 cells and improved behavioral and pathologic phenotypes in transgenic R6/2 HD mice.^754^ PBT2 was also reported to improve motor ability, increase body and brain weight, reduce striatal atrophy of R6/2 mouse, and ameliorate toxicity in a *C. elegans* model of polyQ aggregation.^755^ In 2014, a randomized, double-blind, placebo-controlled trial was conducted for 26 weeks (ClinicalTrials.gov reference: NCT01590888). The early/mid-stage HD patients were administered PBT2 (250 or 100 mg) or placebo once daily, however, neither dose significantly improved cognition on the cognitive tests until the end of the trail. PBT2 was generally safe and well tolerated, a larger study was needed to evaluate potential benefits.^756^ Chen et al. found that intra-ventricular administration of DFO results in an improvement in the motor phenotype in R6/2 HD mice.^392^ Additionally, oral DFP treatment for 10 days on 9-week R6/2 HD mice not only removed mitochondrial iron to decrease lipid peroxidation but also improved motor endurance.^396^

## Conclusion and perspective

Although the contribution of dysregulated metal ions, especially iron deposition, has been implicated in the pathological progression of neurodegenerative diseases and proposed as a promising target strategy for prevention or treatment, the exact distribution map of metal ions among different brain regions, cells, and organelles is still unclear. In patients with PD, AD, ALS, and HD, the iron levels in specific brain regions are significantly elevated, and these iron levels are closely associated to the severity of the disease.^46,47,757,758^ At the brain regional level, the identification of the axonal iron transport pathway from the vHip to the mPFC to the SN may support the hypothesis that dysregulated axonal iron transport results in abnormal iron distribution among different brain regions, particularly in the SN, thereby causing PD-related symptoms.^74,75,759^ It is worth investigating whether abnormal axonal iron transport contributes to iron deposition and subsequently results in motor or non-motor symptoms of neurodegenerative diseases. Further investigations focusing on this hypothesis may provide a new perspective and mechanism for understanding the pathogenesis at the regional level. As demands for iron and other metal ions vary among neurons, glial cells, and different organelles, further investigations focusing on the crosstalk of metal ion distribution among cells and organelles in the CNS may provide a comprehensive understanding of their role in the development of neurodegenerative diseases.

The early diagnosis of neurodegenerative diseases faces multiple challenges. Firstly, the pathological processes of these diseases often initiate many years before clinical symptoms manifest, making it difficult to diagnose them through traditional clinical assessments prior to symptom onset. Secondly, early symptoms are frequently nonspecific and can be mistaken for various other diseases, resulting in misdiagnosis or missed diagnoses. Furthermore, the identification of biomarkers for neurodegenerative diseases remains incomplete, thereby limiting the accuracy and reliability of early detection. Considering the crucial role of metal ions in the development of neurodegenerative diseases, the levels and distribution of these ions can serve as biomarkers, aiding in the early diagnosis of such conditions. Currently, researchers are exploring how these metal ions change in biological samples such as CSF and blood, and their relationship to the pathological features of neurodegenerative diseases. By developing more sensitive biochemical and molecular biology techniques, researchers can quantitatively analyze these metal ions and assess their potential as diagnostic markers. Additionally, higher sensitivity and specificity in MRI imaging, along with the development of specific probes, will also contribute to evaluating changes in metal ion levels in vivo, providing technical support for early disease diagnosis.

Due to complex causative factors, the precise pathophysiological mechanisms underlying neurodegenerative diseases have yet to be fully elucidated. This lack of understanding complicates drug design aimed at targeting specific molecules or pathways. Furthermore, limited specificity and sensitivity in biomarkers present challenges not only for early diagnosis and monitoring disease progression but also result in delays in timely treatment for these conditions. The slow progression of neurodegenerative diseases coupled with specific barriers within the brain makes it challenging to effectively distribute, metabolize, and evaluate new drugs within the body, consequently affecting the efficiency of drug screening and verification. The advantage of metal ion chelators in neurodegenerative diseases lies in their ability to regulate the bioavailability of metal ions, reduce oxidative stress and protein aggregation, thereby providing neuroprotective effects. These advantages make metal ions to be promising targets for the treatment of neurodegenerative diseases. Metal ions, such as iron, manganese, copper, and zinc, summarized in this review, play important roles in numerous biological processes in the brain. However, their accumulation can lead to ferroptosis, cuproptosis, oxidative stress, cell senescence, and neuroinflammation. Therefore, an imbalance of metal ion levels is detrimental. Imbalances in iron metabolism contribute to neuronal death through the ferroptosis pathway. Imbalances in iron metabolism contribute to neuronal death through the ferroptosis pathway, while imbalances in copper metabolism lead to neuronal death *via* the cuproptosis pathway. Therefore, interventions targeting the metabolism of iron or copper, particularly the ferroptosis and cuproptosis pathways, may become treatment strategies for neurodegenerative diseases.^22,152,760,761^ Genetic or pharmacological strategies targeting metal ion distribution at the levels of brain region, cell or organelle may be more effective in modulating motor or non-motor behaviors in neurodegenerative diseases. Chinese herbal medicine contains a large number of natural active compounds, which often exhibit unique pharmacological effects.^762^ Screening natural compounds from Chinese herbal medicine that chelate metal ions and structurally modifying them to improve their selectivity, reduce toxicity, enhance their ability to penetrate the BBB, and develop multimodal therapeutic strategies may be an effective approach for developing new drugs for neurodegenerative diseases.
